# Global Reprogramming of Host SUMOylation during Influenza Virus Infection

**DOI:** 10.1016/j.celrep.2015.10.001

**Published:** 2015-11-05

**Authors:** Patricia Domingues, Filip Golebiowski, Michael H. Tatham, Antonio M. Lopes, Aislynn Taggart, Ronald T. Hay, Benjamin G. Hale

**Affiliations:** 1 MRC-University of Glasgow Centre for Virus Research, Garscube Campus, 464 Bearsden Road, Glasgow G61 1QH, UK; 2 Centre for Gene Regulation and Expression, College of Life Sciences, University of Dundee, Dundee DD1 5EH, UK

## Abstract

Dynamic nuclear SUMO modifications play essential roles in orchestrating cellular responses to proteotoxic stress, DNA damage, and DNA virus infection. Here, we describe a non-canonical host SUMOylation response to the nuclear-replicating RNA pathogen, influenza virus, and identify viral RNA polymerase activity as a major contributor to SUMO proteome remodeling. Using quantitative proteomics to compare stress-induced SUMOylation responses, we reveal that influenza virus infection triggers unique re-targeting of SUMO to 63 host proteins involved in transcription, mRNA processing, RNA quality control, and DNA damage repair. This is paralleled by widespread host deSUMOylation. Depletion screening identified ten virus-induced SUMO targets as potential antiviral factors, including C18orf25 and the SMC5/6 and PAF1 complexes. Mechanistic studies further uncovered a role for SUMOylation of the PAF1 complex component, parafibromin (CDC73), in potentiating antiviral gene expression. Our global characterization of influenza virus-triggered SUMO redistribution provides a proteomic resource to understand host nuclear SUMOylation responses to infection.

## Highlights

• Nuclear-replicating RNA viruses trigger a host SUMOylation response
• Influenza virus RNA polymerase activity contributes to SUMO remodeling
• Influenza virus infection re-targets SUMO to a unique set of 63 host proteins
• SUMOylation of parafibromin (CDC73) potentiates antiviral gene expression

## Graphical Abstract

**Figure:**
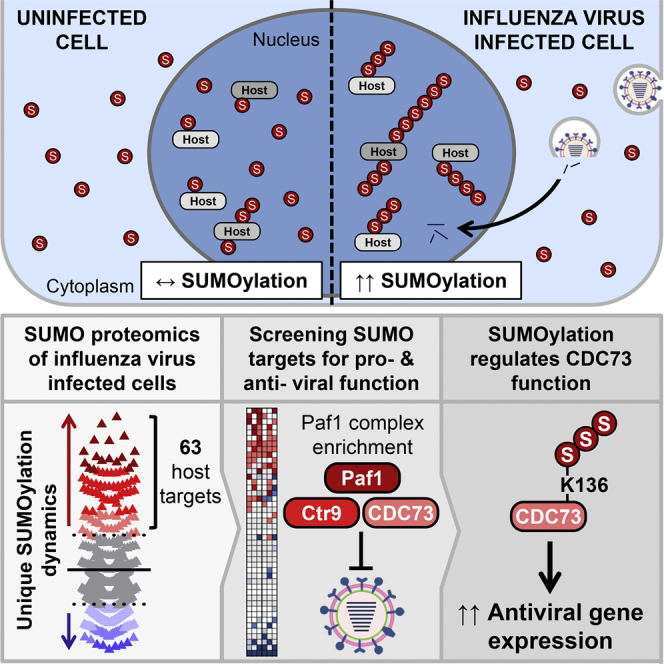

## Teaser

Dynamic SUMOylation is essential for cellular stress responses. Here, Domingues et al. describe a host SUMOylation response to nuclear-replicating influenza viruses. Using quantitative proteomics and functional screening, they reveal that infection uniquely re-targets SUMO to a diverse set of host proteins involved in transcription, RNA processing, and DNA damage repair.

## Introduction

Reversible posttranslational modification of proteins provides cells with a rapid and dynamic mechanism to modulate proteome functionality in response to many stimuli, including pathogen invasion. Ubiquitin and ubiquitin-like modifiers (Ubls) have emerged as central players in mediating the host innate immune response to infection, and their diversity, coordination by specialized enzymatic cascades, and range of linkage topologies all contribute to an incredibly rich regulatory potential. Among Ubls, the small ubiquitin-like modifiers (SUMOs) are predominantly located in the cell nucleus, and they are reversibly attached to lysine residues in target proteins by only a small set of known enzymes (Hay, 2013). Nevertheless, SUMOs can conjugate to thousands of proteins (Hendriks et al., 2014, Tammsalu et al., 2014) and regulate distinct cellular processes, such as transcription, chromatin remodeling, DNA repair, and cell-cycle progression.

The SUMO conjugation machinery is highly responsive to stress stimuli, with global changes to SUMOylation occurring rapidly after cells have been exposed to heat shock (Golebiowski et al., 2009, Saitoh and Hinchey, 2000), proteasome inhibition (Lamoliatte et al., 2014, Tatham et al., 2011), or DNA damage (Hendriks et al., 2015, Yin et al., 2012). Thus, stress-triggered SUMOylation modulates nuclear functions as part of a resolution strategy to protect cell integrity. Recent studies also have implicated SUMOylation as playing a critical role in activating host intracellular pathogen defenses, particularly against DNA viruses that enter the nucleus (Boutell et al., 2011, Cuchet-Lourenço et al., 2011), but also against HIV-1 (Li et al., 2012) and bacteria (Fritah et al., 2014, Ribet et al., 2010). As such, many DNA viruses, as well as some bacteria, encode proteins that actively suppress host SUMOylation or reduce the global amount of SUMO conjugates in infected cells (Everett et al., 2013, Ribet et al., 2010). Notably, with clear parallels to the SUMOylation response triggered by environmental stresses, a nuclear-replicating HSV-1 mutant lacking the ability to degrade SUMO-modified proteins induces increased SUMO conjugate formation during infection and is restricted by an active SUMO system (Boutell et al., 2011). This suggests that cells sense DNA virus infection stress in the nucleus and (in the absence of a pathogen-encoded antagonist) respond by enhancing SUMOylation of certain targets to suppress replication.

Influenza viruses are atypical RNA viruses that replicate in host-cell nuclei and encode multiple proteins that become SUMOylated during infection in order to regulate their trafficking or function (Han et al., 2014, Santos et al., 2013, Wu et al., 2011, Xu et al., 2011). The inextricable linking of influenza viruses to nuclei is further exemplified by the coupling of viral RNA transcription to that of the host, the viral re-purposing of host-cell RNA splicing machinery, and the tethering of viral RNA genomes to cellular chromatin (Fodor, 2013). We speculated that the replication strategy of these nuclear RNA pathogens may induce a form of nuclear stress akin to that of an invading DNA virus, thereby resulting in an analogous host SUMOylation response to resolve infection. Here we show that global remodeling of the host SUMO system is induced by nuclear-replicating influenza virus infections, but not other cytoplasmic-replicating RNA virus infections, and that influenza viral polymerase activity in the nucleus is a key contributor to this SUMO response. Furthermore, we utilize a system-wide quantitative mass spectrometry approach to identify the SUMO-modified proteome of human lung epithelial cells, and we quantify changes in host SUMO modification during influenza A virus (IAV) infection. Combining our proteomic results with a targeted gene-depletion screen and mechanistic studies, we uncover a host SUMOylation response to IAV infection that is distinct from that triggered by other cellular stresses, and we reveal several potential pro- and antiviral host factors whose function is regulated by SUMO. These data form a comprehensive proteomic and functional resource to understand the nuclear SUMO response to an RNA virus infection.

## Results

### Nuclear-Replicating Influenza Viruses Induce Specific Remodeling of Host SUMO Conjugation and Localization

**Figure 1:**
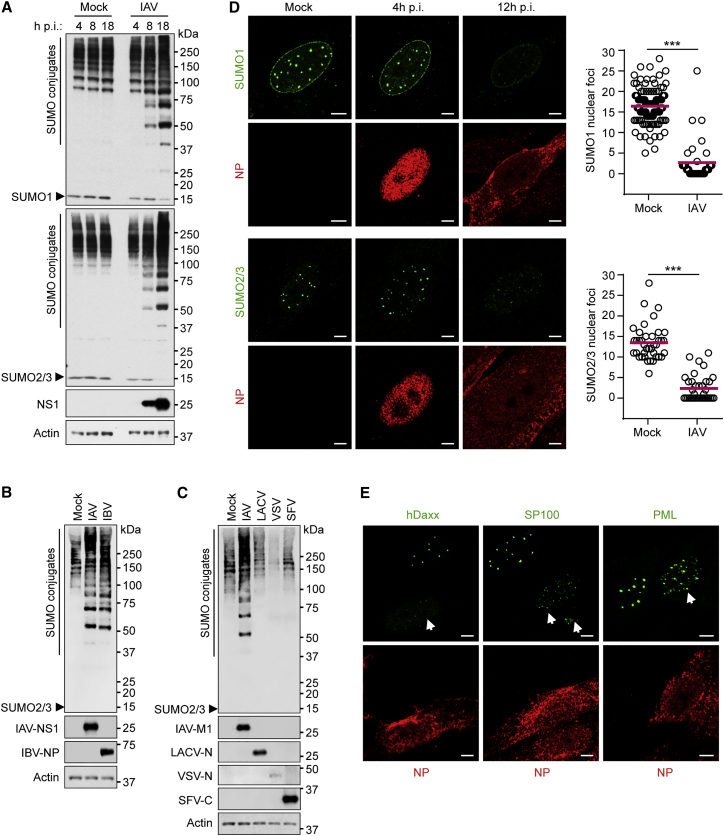
SUMO Conjugation Patterns and Intracellular Distribution following Infection with a Panel of Nuclear- and Cytoplasmic-Replicating RNA Viruses (A) Western blot of lysates from A549s infected with IAV (5 PFU/cell) as indicated. SUMO1, SUMO2/3, NS1, and actin were detected. (B and C) Western blot of Vero cells infected with IAV or influenza B virus (IBV) for 16 hr at 33°C (B) or infected with IAV, LACV, VSV, or SFV for 12 hr at 37°C (all ∼5 PFU/cell) (C). SUMO2/3, actin, and individual viral proteins were detected. (D and E) Immunofluorescent analysis and quantification of MRC5s infected with IAV at 0.1 PFU/cell as indicated. SUMO1 and SUMO2/3 (D) or hDaxx, SP100, PML (E), and IAV NP were visualized after staining. Scale bars represent 5 μm. Statistical significance (^∗∗∗^p < 0.0001) in (D) was determined using the Student’s t test. See also Figure S1.

Human cells express three main SUMO paralogues as follows: SUMO2 and SUMO3, which only differ by 3 amino acids in their mature state (hereafter referred to as SUMO2/3); and SUMO1, which shares ∼50% sequence identity with SUMO2/3. Consistent with the results of others (Pal et al., 2011), western blot analysis of total human lung epithelial cell (A549) lysates revealed that IAV infection triggers an increase in the abundance of proteins modified by both SUMO1 and SUMO2/3, whereas the amounts of free, unconjugated SUMO1 and SUMO2/3 are depleted (Figure 1A). This SUMOylation response is not due to an increase in SUMO mRNA transcripts during infection (Figure S1A), indicating that new SUMO conjugates arise from the pre-existing SUMO pool. Furthermore, this response is not unique to IAV, as influenza B virus (which also replicates in the nucleus) triggered similar SUMO conjugate induction during infection (Figure 1B). Nevertheless, infection with a panel of cytoplasmic-replicating RNA viruses (including members of the *Bunyaviridae* [−ve sense, segmented RNA genome], *Rhabdoviridae* [−ve sense, single-stranded RNA genome], and *Togaviridae* [+ve sense, single-stranded RNA genome]) revealed that these viruses do not trigger gross SUMO conjugate induction (Figure 1C). These data suggest a specific induction of SUMO conjugates in response to nuclear-replicating influenza viruses.

To further characterize SUMO remodeling during IAV infection, we studied the intracellular distribution of SUMO1 and SUMO2/3. SUMOs normally form discrete intra-nuclear foci (10–20 per cell), a fraction of which co-localizes with promyelocytic leukemia (PML) nuclear bodies (PML NBs) (Everett et al., 2013). In addition, a sub-population of SUMO1 localizes to the nuclear rim. We found that, concomitant with SUMO conjugate induction, IAV infection triggers dispersal of both SUMO1 and SUMO2/3 nuclear puncta (Figures 1D and S1B), a phenotype similar to that observed in response to DNA damage and heat shock stresses (Hendriks et al., 2015, Nefkens et al., 2003). Notably, components of PML NBs (such as hDaxx and SP100) also disperse during IAV infection, but PML itself only redistributes into smaller, yet more numerous, foci (Figure 1E). A strikingly similar specific redistribution of PML NB components has been observed previously in response to heat shock (Nefkens et al., 2003). However, western blotting did not reveal gross changes in PML SUMOylation following IAV infection (Figure S1C), suggesting that this SUMO remodeling is specific to certain cellular substrates. These data indicate that IAV-induced SUMO remodeling causes a redistribution of SUMO from sites including PML NBs to new targets that are distributed diffusely throughout the nucleus.

### Influenza Virus RNA Polymerase Activity Contributes to Host SUMO Remodeling

IAV-triggered SUMOylation was not abrogated in cells functionally deficient in the cytoplasmic innate immune mediators MAVS, IRF3, and STAT1 (Figure S2A). Furthermore, there was no change in the kinetics of IAV-induced SUMOylation when small interfering RNAs (siRNAs) were used to deplete infected cells of NS1, the major IAV interferon antagonist (Figure S2B). In addition, an IAV infection-like SUMOylation response was not observed following type I/II interferon stimulation or following stimulation of canonical innate immune responses by a defective-interfering particle-rich stock of Sendai virus (Figure S2C). These data are consistent with previous observations disconnecting IAV-induced SUMOylation from interferon responses (Pal et al., 2011). We therefore hypothesized that a form of viral stress distinct from that triggering classical innate immune pathways may be responsible for IAV-induced SUMOylation. However, an IAV-like broad SUMO response could not be triggered using chemical stimuli promoting ER stress, DNA damage, or apoptosis, three canonical cellular stresses we suspected might occur during IAV infection, but that were not active at times when IAV-stimulated SUMOylation was apparent (Figures S2D and S2E).

**Figure 2:**
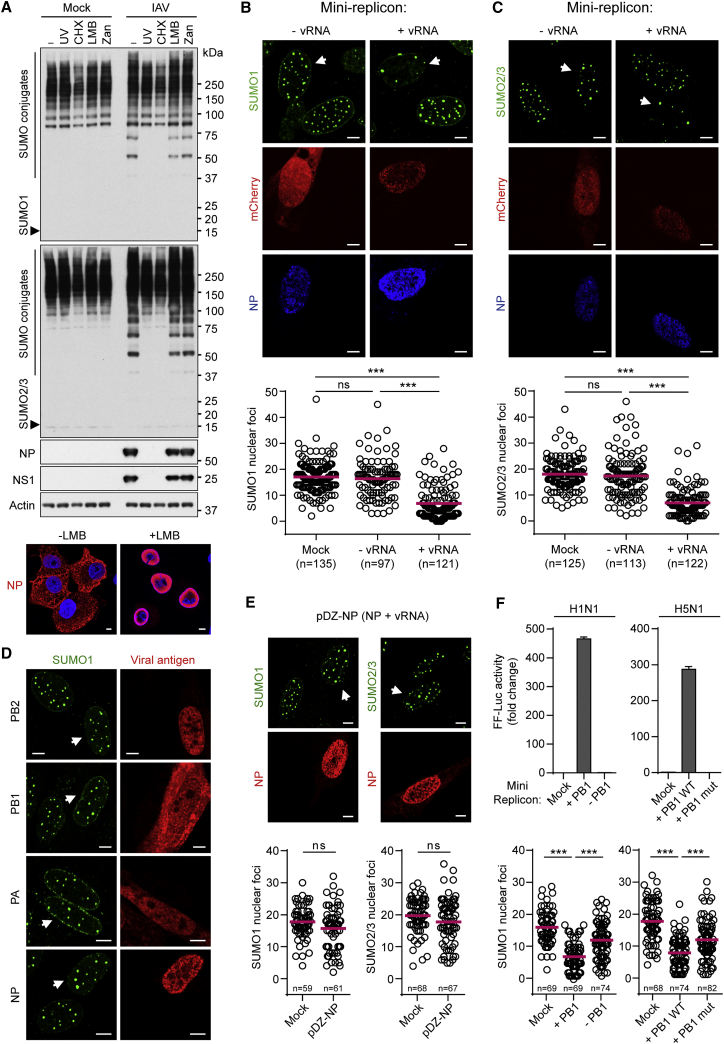
IAV Polymerase Activity Contributes to SUMO Remodeling (A) Western blot of lysates from IAV-infected A549 cells treated with different inhibitors. Cells were infected with IAV or UV-inactivated IAV (UV) at 5 PFU/cell, followed by incubation with 50 μg/ml cycloheximide (CHX), 11 nM leptomycin B (LMB), or 10 μM zanamivir (Zan) for 12 hr. SUMO1, SUMO2/3, NS1, NP, and actin were detected. (Bottom) Immunofluorescence shows NP staining at 12 hr post-infection in A549s ± LMB. DAPI was used to stain DNA. (B and C) Immunofluorescent analyses of MRC5s transiently expressing PB1, PB2, PA, NP, and a negative-sense viral-like mini-replicon mCherry reporter construct (+vRNA), or PB1, PB2, PA, NP, and mCherry (no viral-like reporter; −vRNA). (D) Immunofluorescent analysis of MRC5s individually expressing PB1, PB2, PA, or NP. (E) Immunofluorescent analysis of MRC5s transiently transfected with pDZ-NP, which expresses both NP protein from a pol-II promoter and NP vRNA from a pol-I promoter. (F) (Top) Luciferase-based mini-replicon assays in 293Ts to assess polymerase activity. (Left) (WSN, H1N1) Cells transiently expressing PB1 (or not), PB2, PA, NP, and a negative-sense viral-like mini-replicon Firefly luciferase reporter construct. (Right) (KAN-1, H5N1) Cells transiently expressing PB1 (or an E445A/E446A inactive mutant), AvianPr-PB2-E627K, PA, NP, and a negative-sense viral-like mini-replicon Firefly luciferase reporter construct. Bars represent mean values from triplicates (±SD). (Bottom) Quantification of SUMO1 nuclear foci for the conditions indicated above as determined by the mCherry-based mini-replicon reporter assay in MRC5s. For (B)–(F), cells were transfected for 36 hr prior to processing or fixation and immunostaining. Representative images are shown. Scale bars represent 5 μm. Statistical significance in panels (B), (C), (E), and (F) was determined using the Student’s t test (^∗∗∗^p < 0.0001; ns, non-significant). See also Figure S2.

We used small-molecule inhibitors and UV-inactivation methods to map IAV-triggered SUMOylation to a process requiring viral genome replication and protein synthesis, but not genome nuclear export or later stages of the virus replication cycle, such as virion budding (Figure 2A). Given the tight association of active influenza virus replication complexes with nuclear processes (a distinguishing feature from cytoplasmic-replicating RNA viruses), we speculated that the stress of nuclear IAV polymerase activity may contribute to host SUMO remodeling. To test this hypothesis, we used a transfection-based mini-replicon reporter system, whereby viral ribonucleoprotein complexes (vRNPs) consisting of viral NP, PB1, PB2, and PA are assembled together in the nucleus following plasmid expression along with a negative-sense viral-like RNA genomic segment encoding mCherry. As the viral-like RNA cannot be transcribed into mRNA by cellular polymerases, mCherry protein is only produced in cells expressing all five viral components. Furthermore, the mCherry construct is unspliced such that this assay recapitulates IAV RNA transcription and replication, but not splicing.

Using SUMO foci dispersion as a single-cell readout of host SUMO proteome remodeling, we found that cells expressing actively replicating IAV RNP complexes had significantly fewer SUMO1 and SUMO2/3 foci than cells expressing all the viral protein components of the RNP in the absence of viral-like RNA or mock-transfected cells (Figures 2B and 2C). In addition, expression of each individual vRNP component, or negative-sense viral RNA together with NP, failed to stimulate SUMO remodeling (Figures 2D and 2E). To assess directly the contribution of viral polymerase activity (rather than basic processes such as RNP formation) on SUMO redistribution, we also tested inactive RNPs in this assay, either by omitting an essential polymerase component or by substituting in a mutant polymerase. In both cases, polymerase activity was completely abrogated, as determined by standard mini-replicon assays, and concomitantly there was a significant relief in RNP-triggered SUMO redistribution (Figure 2F). Overall, these data suggest that IAV RNA polymerase activity in the nucleus (transcription processes or replication enhancing vRNA/cRNA levels, but probably not splicing) is a stimulus for triggering remodeling of the host SUMO system during infection, and they are consistent with the hypothesis that virus-induced SUMOylation is specific to nuclear-replicating viruses, rather than RNA viruses in general. We propose that IAV polymerase activity in the nucleus triggers a previously unappreciated form of nuclear stress that is regulated by SUMOylation.

### SILAC-Based Quantitative Proteomics of IAV-Induced SUMO Remodeling

To survey the dynamics of cellular and viral protein SUMOylation during IAV infection, we adopted a quantitative proteomic strategy that has been used previously to identify changes in SUMO modification in response to proteotoxic stresses (Golebiowski et al., 2009, Tatham et al., 2011). We generated A549 cell lines stably expressing either SUMO1 or SUMO2 fused to an N-terminal tandem affinity purification (TAP) tag. An additional A549 cell line stably expressing the TAP tag only (TAP only) was generated as a negative control (Figures S3A and S3B). Both heterologously expressed TAP-SUMO1 and TAP-SUMO2 conjugated to endogenous cellular proteins under normal growth conditions in the respective cell lines, and conjugation of these tagged SUMO forms was robustly enhanced following IAV infection (Figure S3C), indicating that the tag did not interfere with SUMO conjugation and that these constructs faithfully recapitulate endogenous SUMOylation changes in response to infection.

**Figure 3:**
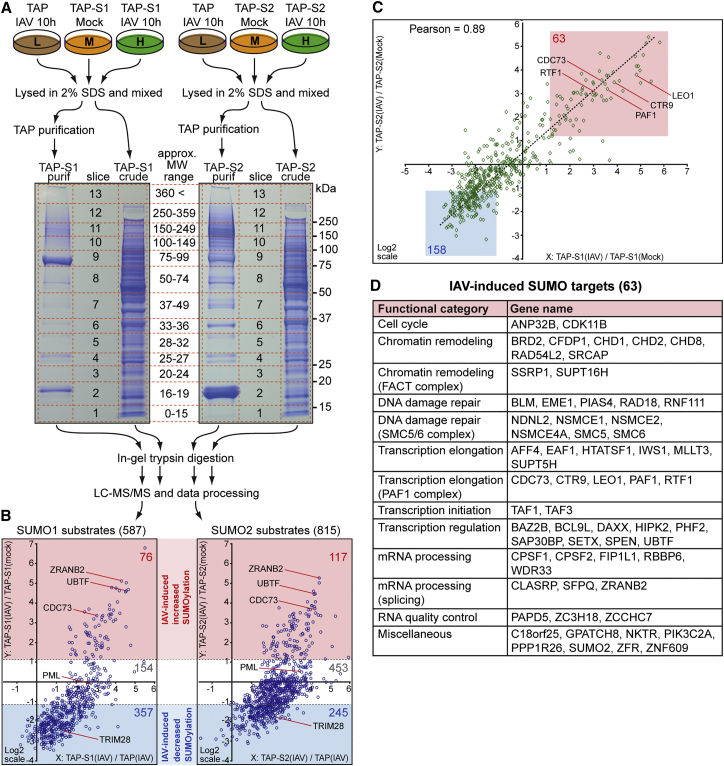
Quantitative SUMO Proteomics of IAV-Infected Cells (A) SILAC-based SUMO1 and SUMO2 proteomic workflow. The specified A549s were grown for five to six cell doublings in light (L; isotopically normal, K0R0), medium (M; K4R6), or heavy (H; K8R10) SILAC medium prior to treatment and processing as indicated. (B) The tsMAPs of SUMO1 (left) and SUMO2 (right) substrates after data filtering, indicating log2-fold changes in protein modification following IAV infection (y axis). The numbers of substrates identified in each category are indicated and certain examples are highlighted. (C) Correlation of log2-fold changes in SUMO1 and SUMO2 substrate modification following IAV infection. The 63 substrates that increase (and 158 substrates that decrease) in both SUMO1 and SUMO2 modification following IAV infection are highlighted, and certain example proteins are labeled. (D) The 63 host substrates that increase in SUMOylation with IAV infection organized by manually curated functional category. See also Figure S3 and Tables S1, S2, S3, S4, and S7.

We conducted two independent stable isotope labeling by amino acids in cell culture (SILAC) experiments to determine the impact of IAV infection for 10 hr on the SUMO1 and SUMO2 sub-proteomes of A549 cells (Figure 3A). This time point was chosen to ensure all infected cells had undergone a full single cycle of virus replication and to capture primary dynamic SUMOylation changes. Notably, <2% of cellular proteins varied in total abundance more than ∼2-fold either between the TAP-only and TAP-SUMO cell lines or after IAV infection (Figures S3D and S3E). In contrast, analysis of the purified samples showed that ∼32% (SUMO1) and ∼47% (SUMO2) of quantified proteins were >2-fold more abundant in the purified TAP-SUMO material compared with the purified TAP-only material, and ∼36% (SUMO1) and ∼25% (SUMO2) of quantified proteins varied >2-fold in abundance in the purified TAP-SUMO material following IAV infection (Figures S3F and S3G). Together, this suggests that a large proportion of identified and quantified proteins in the purified, but not crude, samples show specific changes in abundance relating to SUMO modification status, as well as a dependence upon infection for SUMO conjugation state.

Using a false discovery threshold of 1%, we identified and quantified 587 putative SUMO1 substrates and 815 putative SUMO2 substrates in A549 cells (Figure 3B; Tables S1 and S2). Bioinformatic comparison of the combined 895 putative SUMO substrates with those identified in independent studies using different cell types revealed that 89% of our assigned SUMO substrates have been described as SUMO targets previously (Figure S3H; Table S3). We identified 506 putative substrates as common to both SUMO1 and SUMO2 (Figure S3I), and, consistent with the roles of SUMO, gene ontology analysis using the Enrichr platform (Chen et al., 2013) revealed enrichment of these substrates for cellular compartments including the nucleolus, nucleoplasm, chromatin, and PML NBs as well as molecular function enrichment for chromatin binding, transcription coactivator/corepressor activity, histone binding, and transcription factor binding (Table S3). Furthermore, >76% of these common SUMO substrates recently have been confirmed as bona fide SUMO substrates by high-resolution mass spectrometry-based SUMO modification site-mapping techniques, which identify SUMO-modified lysine residues (Table S4). Such a high degree of overlap with other studies, combined with the gene ontology analysis, supports the validity of our approach in identifying SUMO substrates in human lung A549 cells.

Triple SILAC maps (tsMAPs) of the putative host SUMO substrates illustrated that, surprisingly, the bulk of substrates exhibited reduced SUMO modification (357 for SUMO1 and 245 for SUMO2) or unchanged SUMOylation following IAV infection (Figure 3B; Tables S1 and S2). Strikingly, only 76 SUMO1 substrates (13%) and 117 SUMO2 substrates (14%) increased substantially in SUMO modification status (up to ∼35-fold) during IAV infection (Figure 3B; Tables S1 and S2). Thus, although the original western blotting experiments suggested an overall increase in SUMOylation upon infection, it is clear that IAV-induced SUMOylation of substantial numbers of substrates occurs concomitantly with the deSUMOylation of a different set of proteins. Notably, protein deSUMOylation occurred to a much lower extent (maximum ∼8-fold decrease) than SUMOylation, suggesting widespread dynamic exchange of SUMO during infection from the bulk of pre-existing substrates to a restricted set of new cellular targets.

Comparison of the quantitative changes to SUMO1 and SUMO2 conjugation in response to IAV infection demonstrated a high degree of correlation (Pearson’s coefficient of 0.89) (Figure 3C), indicating no gross differences between SUMO1 and SUMO2 paralogues, although some paralogue-specific SUMO modification changes were observed (Table S3). Thus, the combined SUMO1 and SUMO2 data represent a common and stringent consensus of SUMO substrate changes following IAV infection. Using such criteria, we define 63 host proteins as increasing in SUMO modification with IAV infection and 158 proteins as decreasing in SUMO modification (Figures 3C and 3D; Table S3).

### Validation of IAV-Induced Host SUMOylation Remodeling

**Figure 4:**
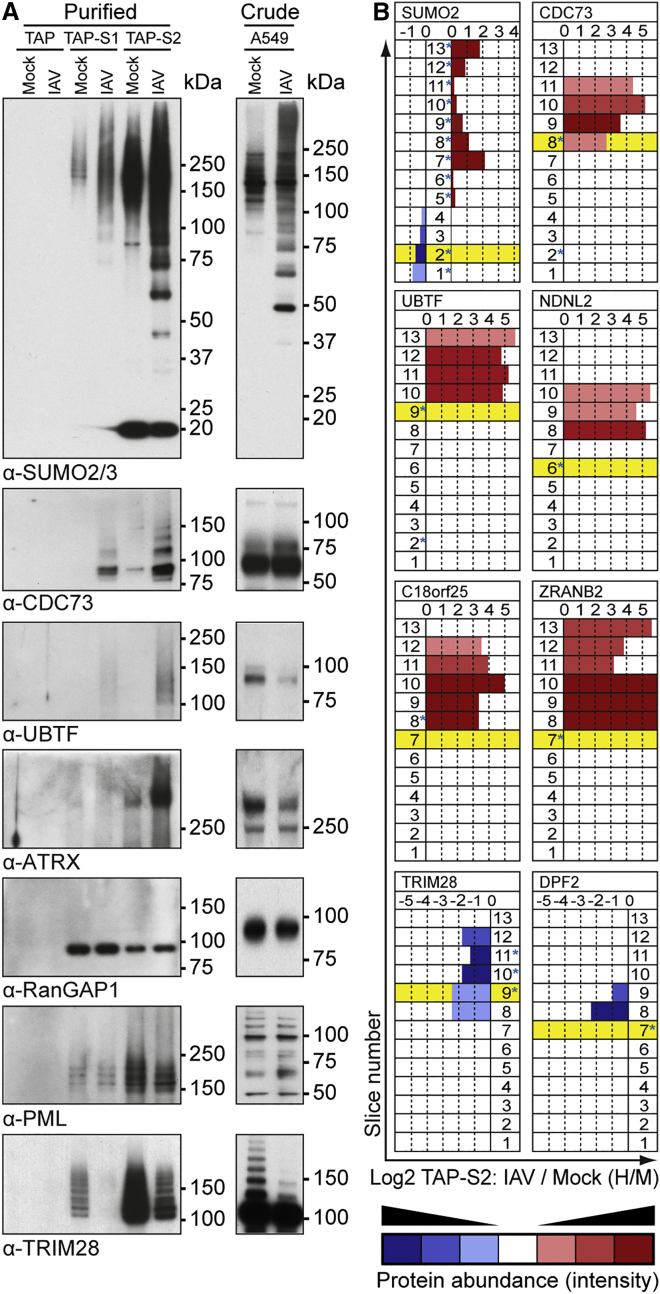
Western Blot and Bioinformatic Validation of IAV-Induced SUMO Targets (A) TAP-purified (left) or crude lysate (right) samples from specified A549s either mock or IAV infected were subjected to western blot analysis for the indicated substrates. (B) Predicted molecular weight (preMW) of substrates (unmodified) was compared to their observed electrophoretic mobility (obsEM) across all 13 slices (crude and TAP-SUMO2 purified, IAV/Mock). Yellow background indicates slice where unmodified protein would be expected based on its preMW. Bar length indicates change in protein ratio between IAV- and mock-infected (H/M) purified TAP-SUMO2 samples. Intensity of color indicates protein abundance (intensity) in each slice; red was nominally given to increased H/M ratios while blue was nominally given to decreased H/M ratios. Asterisks indicate obsEM of specified proteins in crude lysate. See also Figures S4 and S5.

We took both a biochemical and a bioinformatic approach to validate the SILAC ratios obtained for IAV-induced SUMOylation changes. Given that only a small proportion of any given substrate population is usually SUMO modified, detection of conjugated forms of proteins is notoriously difficult without prior enrichment. We therefore used immunoblotting for endogenous cellular proteins to analyze SUMO1 and SUMO2 immunoprecipitation samples from IAV-infected A549s or TAP purification samples from independent IAV infections of the TAP-only, TAP-SUMO1, and TAP-SUMO2 cell lines. As shown in Figures 4A and S4, we could validate the IAV-enhanced conjugation of SUMO1 and SUMO2 to endogenous cellular proteins, such as CDC73, UBTF, and ATRX. Interestingly, we also confirmed IAV-enhanced conjugation of SUMO2 in the TAP-SUMO1 samples, suggestive of a possible increase in SUMO1-capped SUMO2 chains or the increased conjugation of both SUMO1 and SUMO2 to the same target proteins. We also confirmed our mass spectrometry data that RanGAP1 and PML are basally SUMOylated but largely do not change in modification status following IAV infection, whereas TRIM28 is highly deSUMOylated during infection. Importantly, independent western blot analysis of total A549 cell lysates following infection revealed that the abundance of the major unconjugated forms of all these target proteins does not increase in response to IAV infection, an observation consistent with the mass spectrometry quantification of each protein in crude lysates and indicative of a specific effect of infection on SUMO modification status (Figures 4A and S4; Tables S1 and S2).

To further validate the IAV-triggered host SUMOylation changes for additional targets without suitable antibodies available, we developed a slice-by-slice bioinformatic analysis based on scrutinizing the change in electrophoretic mobility of target proteins in TAP-SUMO2-purified mass spectrometry samples in response to IAV infection. First, using mass spectrometry data obtained from individual gel slices, we compared the migration pattern of selected putative targets in total crude lysates with their migration pattern in TAP-SUMO2-purified samples, defining each target as SUMO2 modified if it migrated slower in the purified sample than expected based on its predicted molecular weight. In addition, for each target and gel slice, we analyzed the change in peptide ratio upon IAV infection in the TAP-SUMO2-purified samples in order to determine the magnitude of SUMOylation change at a given mobility.

As shown in Figure 4B, such an analysis of SUMO2 revealed that during IAV infection SUMO2 shifted from a faster migrating species (its unconjugated form) to several slower migrating species distributed throughout multiple gel slices, indicating that increased SUMO2 conjugation during IAV infection is partly a consequence of depleting unmodified SUMO2. Strikingly, similar analyses revealed that host protein examples, such as CDC73, UBTF, NDNL2, C18orf25, and ZRANB2, all migrated slower than expected in the TAP-SUMO2-purified samples (indicative of their SUMO2 modification), and their abundance in these slower migrating forms was highly enhanced upon IAV infection (indicative of increased SUMOylation). In contrast, host proteins such as TRIM28 and DPF2 also migrated slower than expected in TAP-SUMO-purified samples, although their abundance in these fractions decreased with infection, suggesting a decrease in their SUMOylation that correlated with the mass spectrometry and western blot analyses (Figure 4B). These validation examples further strengthened the confidence in our mass spectrometry dataset as a whole.

### IAV Proteins as SUMO Targets

Several IAV proteins have been described as targets for SUMO modification (Pal et al., 2011, Santos et al., 2013, Wu et al., 2011, Xu et al., 2011). Analysis of our TAP-SUMO-enriched mass spectrometry data using a stringent 1% false discovery threshold and our slice-by-slice bioinformatic strategy revealed that only NS1, M1, and NEP satisfied our filtering criteria for SUMO1- and SUMO2-modified targets during infection (Figures S3F, S3G, and S5A). NS1 and M1 have been studied extensively as SUMO targets, but NEP has not previously been identified as a bona fide SUMO substrate during IAV infection. These mass spectrometry data therefore support the rationale for future studies investigating functional consequences of viral protein SUMOylation.

### IAV-Induced Host SUMOylation Responses Are Distinct from Those Triggered by Canonical Stress Stimuli

**Figure 5:**
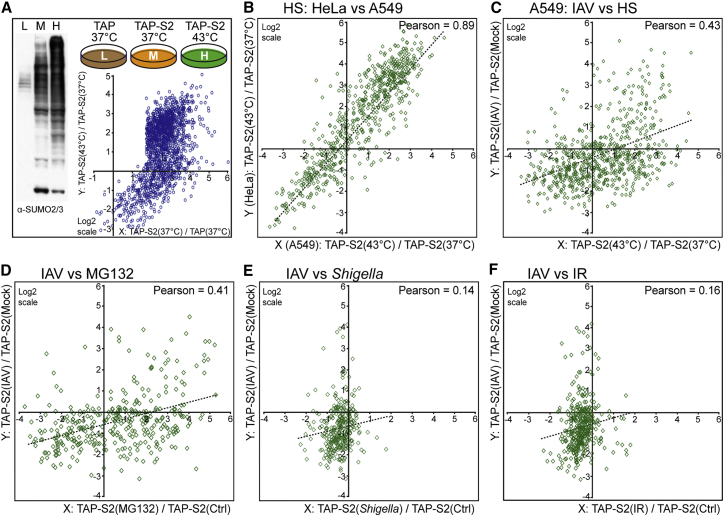
The IAV-Induced Host SUMOylation Response Is Quantitatively Distinct from that Induced by Other Stresses (A) Quantitative SUMO2 proteomics of the heat shock response in A549s. The indicated A549s were grown for five to six cell doublings in light (L; isotopically normal, K0R0), medium (M; K4R6), or heavy (H; K8R10) SILAC medium prior to treatment (for 30 min) as indicated. Subsequent TAP purification and analyses were performed as for Figure 3. Graph shows tsMAP of SUMO2 substrates after data filtering, indicating log2-fold changes in protein modification following heat shock (y axis). (B–F) Correlations show log2-fold changes in SUMO2 modification in response to heat shock between HeLa and A549 cells (B); between IAV infection and heat shock in A549 cells (C); between IAV infection in A549 cells and proteasome inhibition (MG132) in HeLa cells (D); between IAV infection in A549 cells and *Shigella flexneri* infection in HeLa cells (E); and between IAV infection in A549 cells and ionizing radiation (IR; 15 Gy) in HeLa cells (F). See also Figure S5 and Table S5.

A multitude of stresses has been demonstrated to modulate cellular SUMO modification dynamics, including heat shock, ionizing radiation, proteotoxicity, and bacterial infection. Strikingly, heat shock causes a global increase in total SUMO conjugates that, by western blot, appears similar to that observed with IAV infection (Figure 5A). To compare the cellular SUMOylation response to IAV with the response to heat shock treatment, we also used SILAC proteomics to determine how the SUMO2-modified proteome changes in TAP-SUMO2 A549 cells following incubation at 43°C for 30 min (Figure 5A; Table S5). Heat-shock-triggered SUMO2 conjugation changes in A549s strongly correlated with those previously determined in HeLa cells using similar methodologies (Golebiowski et al., 2009), thereby confirming the validity of our data and indicating that this cellular stress induces a cell-type-independent SUMO response (Pearson’s coefficient of 0.89, Figure 5B). Nevertheless, the IAV-triggered SUMOylation response in A549s did not quantitatively correlate with the heat shock SUMOylation response (Pearson’s coefficient of 0.43) (Figure 5C).

Furthermore, given that our analysis of A549 and HeLa heat shock data indicated a common SUMO response in both A549 and HeLa cells, we took advantage of several HeLa SUMO proteomic datasets generated under various stress conditions (Fritah et al., 2014, Tatham et al., 2011, Yin et al., 2012) and compared them with our IAV-induced SUMO response in A549s. Notably, we also did not find a correlation between the IAV-induced SUMO response and the SUMO responses to proteasome inhibition (MG132 treatment), infection with *Shigella flexneri*, or ionizing radiation (Figures 5D–5F). On a qualitative level, bioinformatic analysis revealed distinct pathways that were enriched in enhanced SUMOylation following individual stresses (Table S7). For example, IAV-induced SUMOylation targets were enriched in members of the human PAF1 complex (PAF1C) and several categories relating to RNA polymerase II function, while *Shigella flexneri*-induced targets were enriched in centromer chromatin complex members. Heat shock stress caused SUMO to redistribute to a wider range of targets, with the most enriched categories including the spliceosome, the polycomb repressive complex, and the DNA synthesome complex (Table S7). These comparative observations suggest that IAV infection triggers a host SUMOylation response that is phenotypically distinct from responses to canonical cellular stresses, highlighting a potentially unique stress to the cell induced by viral RNA polymerase activity in the nucleus.

### shRNA Screening Identifies IAV-Triggered SUMO Targets as Pro- and Antiviral Host Factors

**Figure 6:**
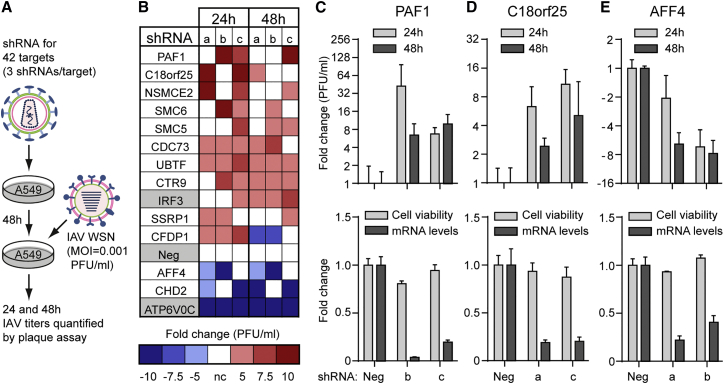
SUMO Targets Impacting IAV Replication (A) Schematic representation of the lentivirus-based shRNA screen assessing 42 host SUMO targets for their impact on IAV replication. (B) Heatmap summary of factors identified as required or restrictive to IAV replication in A549 cells. Genes are shown whose depletion led to a 5-fold or more difference in infectious IAV titer as compared with control for at least two of three shRNA sequences. Each individual shRNA is labeled a, b, or c and control shRNAs are highlighted in gray. (C–E) Validation of PAF1 (C), C18orf25 (D), and AFF4 (E) as impacting IAV replication. The two shRNA sequences for each gene from (B) that showed consistent impact on IAV replication were independently validated in the same assay for their effect on IAV replication (top) and specific gene knockdown and effect on cell viability (bottom). Bars represent mean values from triplicates (±SD). See also Table S6.

Consistent between our SUMO1 and SUMO2 proteomic studies, IAV infection triggered a >4-fold increase in SUMOylation of 42 host proteins. To identify functional roles for these core SUMO targets during IAV infection, we depleted A549 cells of the corresponding 42 genes one by one using small hairpin RNA (shRNA)-expressing lentiviruses (three per gene), and we determined the subsequent replication of IAV by measuring infectious virus yields at 24 and 48 hr post-infection (Figure 6A). As controls, we also assessed the impact on IAV replication of depleting IRF3, a host antiviral defense transcription factor, and ATP6V0C, a vacuolar ATPase component required for efficient IAV entry (König et al., 2010). We classified a host gene as important for IAV replication if at least two of three shRNAs increased or decreased infectious IAV yields by at least 5-fold at a minimum of one time point compared to the non-targeting shRNA.

Using these criteria, we identified ten putative host antiviral factors among the IAV-induced SUMO targets and two required factors (Figure 6B; Table S6). For several of these factors, independent experiments confirmed that the shRNAs efficiently depleted the target host mRNA, had minimal effect on cell viability, and reproducibly impacted IAV replication (Figures 6C–6E; Table S6). Notably, our shRNA screen identified three members of the human SMC5/6 complex (SMC5, SMC6, and the SUMO E3 ligase NSMCE2) as potential IAV antiviral factors, along with three members of PAF1C (PAF1, CTR9, and CDC73) and two PAF1C-associated proteins (SSRP1 and CFDP1). These data indicate that the IAV-triggered SUMOylation response targets both pro- and antiviral host factors, and they suggest a thus far unknown role for the human SMC5/6 DNA damage repair complex in IAV restriction.

### SUMOylation of CDC73 Promotes Antiviral Gene Expression

In our proteomic screen, all components of PAF1C increased highly in SUMOylation during IAV infection (Figure S5B), and PAF1C was the most highly enriched functional group among all the substrates with infection-enhanced SUMOylation (Table S7). An antiviral role for the PAF1 component of PAF1C during IAV infection has been previously attributed to its potentiation of interferon-stimulated gene (ISG) expression (Marazzi et al., 2012). However, the contribution of SUMO modification to this process is unknown. To explore mechanistically how IAV-triggered SUMOylation may impact the function of PAF1C, we focused follow-up studies on CDC73 (also known as parafibromin or HRPT2), a core component of PAF1C with SUMO modification sites recently mapped by mass spectrometry (Hendriks et al., 2014, Lamoliatte et al., 2014, Tammsalu et al., 2014), and which our shRNA screening data revealed as a potential host antiviral factor.

**Figure 7:**
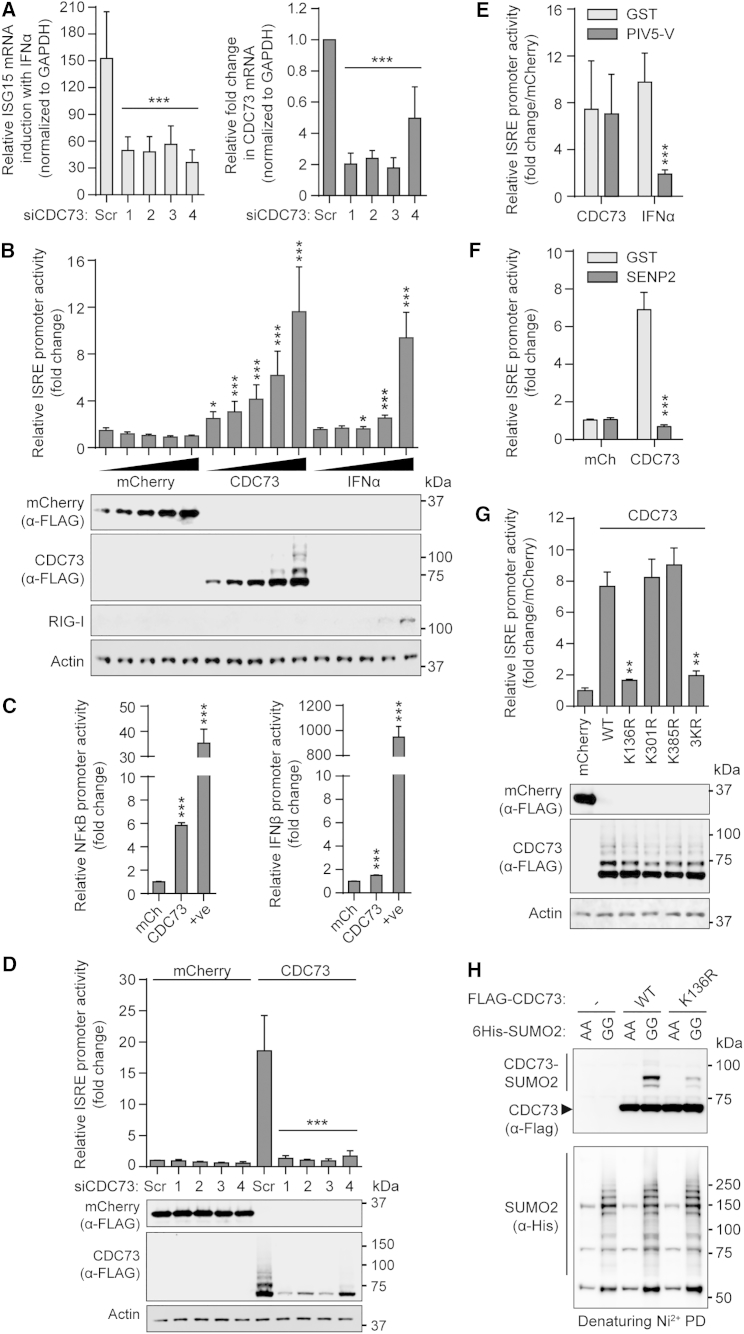
SUMO Modification Promotes the Function of CDC73 in Mediating an ISG Response (A) Impact of CDC73 knockdown on ISG expression. A549s were transfected for 48 hr with four independent siRNAs targeting CDC73 (or scrambled) before stimulation with 100 IU/ml IFNα for 8 hr. The mRNA levels of CDC73, ISG15, and GAPDH were quantified. (Left) IFNα-mediated induction of ISG15 mRNA relative to mock is shown. (B) Induction of an ISRE-containing promoter by overexpressed CDC73. 293Ts were co-transfected with expression plasmids encoding FLAG-tagged mCherry or CDC73 (12.5–200 ng) together with pGL3-Mx1P-FFluc and pRL-SV40. After 36 hr, FF luciferase activity was determined and normalized to *Renilla*. Parallel samples were harvested for western blot, probing for the indicated proteins. (C) Impact of CDC73 overexpression on NF-κB (left) and IFNβ (right) promoters. 293Ts were co-transfected with FLAG-tagged mCherry or CDC73 (200 ng) together with pNF-κB-FFLuc (or p125-FFLuc) and pRL-SV40. Control for NF-κB promoter activation was 10 ng/ml TNF-α for 12 hr (+ve); control for IFNβ promoter activation was co-transfection of 20 ng RIG-I2CARD (+ve). After 36 hr, relative activity was determined as in (B). (D) The siRNAs targeting CDC73 abrogate the effect of CDC73 overexpression on inducible gene expression. 293Ts were transfected/processed as in (B) except four independent siRNAs targeting CDC73 (or scrambled) also were transfected. (E) CDC73-mediated induction of an ISRE-containing promoter is independent of STAT1 function. 293Ts were co-transfected with expression plasmids encoding FLAG-tagged mCherry or CDC73 (100 ng) together with plasmids encoding GST or PIV5-V (100 ng) and pGL3-Mx1P-FFluc and pRL-SV40. Control cells were stimulated with 100 IU/ml IFNα. Then, 36 hr post-transfection, relative activity was determined as in (B). Data represent fold induction in promoter activation relative to mCherry-expressing cells. (F) CDC73-mediated induction of an ISRE-containing promoter is dependent on SUMOylation. Experiment was as in (E) except plasmids encoding GST or SENP2 (100 ng) were co-transfected. (G) The K136 SUMOylation site in CDC73 is essential for stimulating inducible gene expression. Experiment was as in (B) but included a panel of FLAG-tagged CDC73 lysine mutants (wild-type [WT]; K136R; K301R; K385R; or a triple mutant, 3KR). (H) K136 is a major SUMOylation site in CDC73. 293Ts were co-transfected with expression plasmids encoding FLAG-tagged CDC73-WT or CDC73-K136R, together with 6His-tagged SUMO2-GG or SUMO2-AA. Following denaturing Ni^2+^ pull-down, purified proteins were detected by western blot with anti-6His or anti-FLAG. For all graphs, bars represent mean values from triplicates (±SD) and are derived from three independent experiments. Statistical significance was determined using the Student’s t test (^∗^p < 0.01, ^∗∗^p < 0.001, and ^∗∗∗^p < 0.0001; ns, non-significant). See also Figure S6.

In agreement with the antiviral role of PAF1 in mediating RNA polymerase II transcription elongation of ISGs (Marazzi et al., 2012), we found that siRNA-mediated depletion of endogenous CDC73 resulted in defective induction of ISG15 mRNA following IFNα treatment (Figure 7A). In addition, overexpression of CDC73 alone was able to stimulate expression from a promoter containing an interferon-stimulated response element (ISRE) in a dose-dependent manner (Figure 7B). The effect of CDC73 overexpression was not limited to ISRE-containing promoters, as a similar enhancing effect was observed for an NF-κB promoter, although minimally for the IFNβ promoter reporter, indicating a degree of specificity in CDC73’s capacity to regulate inducible gene expression (Figure 7C). Promoter stimulation in these assays was specific to CDC73 overexpression, as co-transfection of siRNAs targeting CDC73 mRNA ablated protein production downstream of the ISRE promoter (Figure 7D). Furthermore, consistent with a model for CDC73 acting in RNA polymerase II-mediated transcription elongation, we found that the effect of CDC73 on ISRE promoter-driven expression was insensitive to depletion of the STAT1 transcription factor, which is otherwise essential for IFNα-stimulated activation of the ISRE (Figure 7E). These data suggest that CDC73 may act as an antiviral factor by potentiating inducible antiviral gene expression at a level subsequent to transcription factor activation.

To evaluate the role of CDC73 SUMO modification in transcription of inducible genes, such as ISGs, we assessed the ability of CDC73 to stimulate the ISRE reporter in the context of co-expressed human SENP2, a deSUMOylating enzyme previously implicated in regulating host antiviral responses (Ran et al., 2011). We found that SENP2 was able to antagonize CDC73-mediated activation of the ISRE reporter, suggesting that SUMOylation of CDC73 is important for this function (Figure 7F). In addition, we screened the ISRE-stimulating capabilities of a panel of three CDC73 mutants with lysine-to-arginine substitutions at sites positively identified by mass spectrometry to be SUMO modified using high-confidence remnant immunoaffinity profiling methods (Lamoliatte et al., 2014, Tammsalu et al., 2014). Notably, the single K136R substitution abrogated CDC73-mediated ISRE-dependent expression, while arginine substitutions at lysines 301 and 385 did not abrogate this response (Figure 7G).

Recent studies have validated CDC73-K136 as a bona fide SUMO modification site in vitro using purified recombinant proteins (Lamoliatte et al., 2014). We found that K136 is a major site for CDC73 SUMOylation in transfected cells, with the K136R substitution alone leading to highly reduced levels of SUMO-modified CDC73 (Figure 7H). K136 is located within an NLS of CDC73, and the arginine substitution at this site also leads to a subtle shift in the nuclear-cytoplasmic distribution of CDC73 (Figure S6), which may be indicative of SUMO modification contributing to the nuclear retention of CDC73. We note that CDC73 SUMOylation can be enhanced by other cellular stresses, including heat shock (Figure S5C), and interestingly the CDC73-K136R SUMOylation mutant has been previously shown to be defective in localizing to PML NBs in response to proteotoxic stress (Lamoliatte et al., 2014). Based on these data, we propose a general function for stress-triggered CDC73 SUMOylation in regulating stress-inducible genes that are required for resolution of cell integrity. With regard to virus infection, IAV-induced SUMOylation of CDC73 appears to potentiate its function in transcription elongation of genes promoting antiviral immunity.

## Discussion

The role of SUMO modifications in resolving cellular stress conditions and responding to DNA virus infections in the nucleus is well established (Everett et al., 2013, Hay, 2013). Here we report the induction of a host SUMOylation response to nuclear-replicating RNA viruses, exemplified by the important human and animal pathogens, influenza A and B viruses. For nuclear-replicating DNA viruses, the incoming naked DNA molecule has been suggested to trigger SUMOylation in a manner analogous to damaged cellular DNA (Cuchet-Lourenço et al., 2011). In our study, we found that active viral RNA polymerase function is a major trigger for SUMO remodeling, suggesting that this foreign activity in the nucleus induces a previously unappreciated form of stress to which the host raises a SUMO response. It is currently unknown which aspect of viral RNA polymerase activity might induce SUMO remodeling, although our unspliced replicon data suggest that viral hijack of the host RNA-splicing machinery is not a major stimulus. A key question to resolve will be whether IAV-induced SUMOylation is a specific response to infection or a generalized response to nuclear stress. For example, with parallels to cytoplasmic RIG-I, a nuclear pathogen sensor might be activated by newly synthesized IAV RNA to stimulate SUMOylation. Alternatively, the cell could simply be reacting to infection-driven changes in the levels of specific host factors, such as ribonucleosides depleted by IAV polymerase activity or uncapped host RNAs generated by viral cap snatching. Given the close physical association of IAV RNPs with host chromosomes (Chase et al., 2011), it is possible that infection induces a non-canonical DNA damage-like SUMO response. In this regard, tethering of viral RNAs to chromatin may mimic aberrant RNA:DNA hybrids reminiscent of R loops, a situation in which SUMOylation plays an important resolving role (Richard et al., 2013).

Several studies have shown that SUMO is important for different aspects of IAV replication, predominantly by directly modifying viral proteins (Han et al., 2014, Santos et al., 2013, Wu et al., 2011, Xu et al., 2011). Experimentally assessing the global contribution of SUMOylation to virus infection is confounded by the integral nature of key SUMO components to cellular activities. For example, the sole SUMO E2 enzyme (Ubc9), several SUMO proteases, and SUMO2 itself are all essential for embryonic development (Kang et al., 2010, Nacerddine et al., 2005, Wang et al., 2014), and SUMO1-deficient mice are only viable due to functional compensation by SUMO2/3 (Evdokimov et al., 2008). Furthermore, depletion of Ubc9 is difficult to establish in tissue culture and results in extensive cell-cycle defects and loss of cell viability (Boutell et al., 2011). Thus, in this study, we rationally undertook to identify and characterize only the IAV-responsive SUMO proteome. Using quantitative proteomics, we revealed an IAV-driven reprogramming of host SUMOylation that is both quantitatively and qualitatively distinct from previously characterized SUMO stress responses. This dataset provided the framework for us to perform targeted functional analysis of a specific IAV-induced SUMO substrate, CDC73, without the need to deplete the entire SUMO system. Our data provide a resource to continue such studies with other IAV-induced SUMO substrates.

Proteins that change in SUMOylation status during IAV infection are involved in a diverse range of nuclear biological processes and regulatory pathways (Figure 3D; Table S7). Consistent with viral RNA polymerase activity triggering SUMO remodeling, we found that IAV infection retargets SUMO to many proteins involved in chromatin remodeling or RNA polymerase II transcription, including chromodomain DNA-binding helicases (CHD1, CHD2, and CHD8), the FACT complex (SSRP1 and SUPT16H), transcription initiation factors (TAF1 and TAF3), the PAF1 complex (PAF1, CTR9, RTF1, LEO1, and CDC73), and other transcription elongation factors (AFF4, EAF1, HTATSF1, IWS1, MLLT3, and SUPT5H). Several infection-induced SUMO targets also are involved in mRNA maturation events, such as 3′ end pre-mRNA processing (CPSF1, CPSF2, FIP1L1, RBBP6, and WDR33), splicing (CLASRP, SFPQ, and ZRANB2), and nuclear RNA quality control (ZC3H18, ZCCHC7, and PAPD5).

In addition, despite being unable to detect an IAV-induced canonical DNA damage response, we identified an infection-responsive increase in the SUMOylation of a remarkable number of host proteins that function in DNA damage repair, such as BLM, EME1, the SUMO E3 ligase PIAS4, the ubiquitin E3 ligases RAD18 and RNF111, and almost all members of the human SMC5/6 complex. Functional screening by ourselves and others has shown that a number of these IAV-induced SUMO targets are required for efficient IAV propagation (Karlas et al., 2010, Landeras-Bueno et al., 2011, Naito et al., 2007) or act as IAV restriction factors (Marazzi et al., 2012). Notably, we identified three members of the SMC5/6 complex (SMC5, SMC6, and the SUMO E3 ligase NSMCE2) as potential antiviral factors, indicating that DNA damage repair proteins may play additional roles in resolving IAV infection. These data suggest that nuclear RNA virus replication stress can be channelled into SUMO-dependent effector pathways shared with cellular DNA repair processes.

We identified all components of the PAF1 complex as IAV-triggered SUMO targets. PAF1 itself, as well as CTR9, recently has been implicated as a positive regulator of antiviral and pro-inflammatory gene expression (Marazzi et al., 2012, Parnas et al., 2015, Youn et al., 2007), and genetic deletion of the CDC73 component of PAF1C in mouse embryonic fibroblasts led to reduced expression levels of several known ISGs, including *Ddx58*, *Trim21*, *Mov10*, *Isg20*, *Stat2*, and *Bst2* (Wang et al., 2008). Here our mechanistic studies revealed a role for SUMOylation of CDC73 in promoting ISG expression. These data thereby directly link one consequence of the IAV-triggered SUMOylation response to antiviral defense. Given that CDC73 SUMOylation has been described to be important for its trafficking in response to proteotoxicity (Lamoliatte et al., 2014) and CDC73 SUMOylation also is enhanced by heat shock, we hypothesize a general function for stress-triggered CDC73 SUMOylation in regulating stress-inducible gene expression.

Our SUMO proteomic datasets and functional characterization now provide a platform to address the role of selected protein groups and their SUMO modification in the IAV replication cycle. This is complemented by existing proteomic studies that recently have mapped SUMO modification sites in most of the targets we identified here (Table S4). IAV infection causes a global reprogramming of the host SUMOylation landscape, the specific temporal dynamics of which have yet to be fully explored. The resources presented here will add a layer of post-translational understanding to previous transcriptomic, proteomic, and genome-wide depletion studies that have sought to gain insights into the extensive interplay between influenza viruses and their hosts (Josset et al., 2014, Karlas et al., 2010, König et al., 2010, Shapira et al., 2009).

## Experimental Procedures

### Cells and Viruses

HEK 293T, A549, Vero, and MDCK cells were maintained in DMEM supplemented with 10% (v/v) fetal calf serum (FCS), 100 units/ml penicillin, and 100 μg/ml streptomycin (Life Technologies). MRC5 cells were maintained in Eagle’s minimal essential medium (EMEM, Sigma) supplemented with 10% (v/v) FCS, 100 units/ml penicillin, 100 μg/ml streptomycin, 2 mM L-Glutamine, and 1% (v/v) Non-Essential Amino Acids (Life Technologies). Generation of A549 cells stably expressing TAP or TAP-SUMO proteins is described in the Supplemental Experimental Procedures. IAV (A/WSN/33) was propagated and titrated by standard plaque assay in MDCKs, while influenza B virus (B/Yamagata/88) and Sendai virus were propagated in 10-day-old embryonated chicken eggs. La Crosse encephalitis virus (LACV), vesicular stomatitis virus (VSV), and Semliki Forest virus (SFV) were propagated and titrated by plaque assay in Vero cells. UV inactivation of IAV was performed on ice with UV irradiation (254 nm) for 1 min at a distance of 7 cm. PCR-based analyses, luciferase reporter assays, cell viability, and statistical methods are detailed in the Supplemental Experimental Procedures.

### Immunodetection Analyses

For western blots, samples were lysed 1:1 in either 2× urea disruption buffer (6 M urea, 2 M β-mercaptoethanol, and 4% SDS) or 2× Laemmli’s sample buffer, nucleic acids sheared by passing three times through a 29G needle, and boiled for 10 min prior to protein separation by SDS-PAGE on NuPAGE Novex 4%–12% Bis-Tris gradient gels (Life Technologies). Proteins were detected by western blotting following transfer to polyvinylidene fluoride (PVDF) membranes. Antibodies used, as well as immunofluorescence assays, are described in the Supplemental Experimental Procedures.

### SILAC Cell Culture, Treatments, and TAP Purification

Proteomic experiments were performed using the SILAC technique that allows for quantitative data analysis. In brief, cells were grown in DMEM with L-lysine and L-arginine replaced with stable isotope forms (Cambridge Isotope Laboratories) in various combinations depending on treatment (see the Supplemental Experimental Procedures). SILAC DMEM was supplemented with 10% dialyzed FCS. The modified denaturing TAP procedure has been described previously (Golebiowski et al., 2010). In brief, after treatment, cells were washed with PBS and lysed with denaturing buffer containing 2% SDS. For large-scale mass spectrometry experiments with three conditions, all resulting lysates were mixed 1:1:1 (based on total protein), and a crude sample (∼1% of the total) was analyzed separately from the remaining ∼99% material, which was subjected to TAP purification (see the Supplemental Experimental Procedures). Both samples were resolved on NuPAGE Novex 10% Bis-Tris polyacrylamide gels prior to gel slice excision, in-gel tryptic digestion, and liquid chromatography-tandem mass spectrometry (LC-MS/MS). Detailed methods as well as information on data processing are included in the Supplemental Experimental Procedures. Small-scale purifications were done in essentially the same way, although lysates were handled separately and analyzed by SDS-PAGE and western blot.

### shRNA Lentivirus Library Preparation and Functional Screening

A customized 129 component sequence-verified MISSION shRNA lentiviral plasmid (pLKO.1-puro) library targeting 44 genes of interest (as well as a negative control scramble sequence) (Table S6) was purchased from Sigma-Aldrich. Lentiviral stocks were prepared by co-transfecting each pLKO.1-puro plasmid with pMD2.G and pCMVdR8.91 into 293T cells using PEI. Lentiviral supernatants were harvested 60 hr post-transfection, aliquoted, and stored at −80°C. For screening gene depletion impact on IAV, A549 cells in 24-well plates were transduced with the appropriate lentivirus stock for 48 hr in the presence of 8 μg/ml polybrene (Millipore). Very high transduction efficiency for a subset of our lentiviral preparations was confirmed using puromycin treatment of parallel plates. Transduced cells were infected with WSN at an MOI of 0.001 plaque-forming units (PFU)/cell, and supernatants were collected and titrated by plaque assay at 24 and 48 hr.

## Author Contributions

P.D. designed, performed, and analyzed the cell biology and virology experiments. F.G. designed, performed, and analyzed the proteomic experiments. M.H.T. performed mass spectrometry and contributed to data analysis. A.M.L. generated new reagents. A.T. contributed to virology. R.T.H. designed experiments and interpreted results. B.G.H. led the project, designed experiments, interpreted results, and wrote the manuscript.

## Accession Numbers

The mass spectrometry proteomic data have been deposited to the ProteomeXchange Consortium (Vizcaíno et al., 2014) via the PRIDE partner repository with the dataset identifier PXD002943.

## Supplemental Information

Document S1. Supplemental Experimental Procedures and Figures S1–S6

Table S1. SUMO1 Proteomic Dataset in Uninfected and IAV-Infected A549s, Related to Figure 3

Table S2. SUMO2 Proteomic Dataset in Uninfected and IAV-Infected A549s, Related to Figure 3

Table S3. Summary of Common and Unique SUMO Targets in A549s and the Impact of IAV Infection, Related to Figure 3

Table S4. Summary of Common SUMO Targets in A549s and the Overlap with Previous SUMO Site-Specific Proteomic Mapping Studies, Related to Figure 3

Table S5. SUMO2 Proteomic Dataset in Unstimulated and Heat-Shocked A549s, Related to Figure 5

Table S6. Primary shRNA Screening Data and Validations for the Role of Host SUMO Targets during IAV Infection, Related to Figure 6

Table S7. Enrichr Pathway Analysis for Functional Classification of Host Substrates that Change in SUMOylation during IAV Infection and Other Stresses, Related to Figure 3

Document S2. Article plus Supplemental Information
