# Supplementary material for: Global Reprogramming of Host SUMOylation during Influenza Virus Infection
Source: Cell Rep. 2015 Nov 5;13(7):1467–80. doi: 10.1016/j.celrep.2015.10.001 (PMC4660286; doi:10.1016/j.celrep.2015.10.001)
Supplement: Document S1. Supplemental Experimental Procedures and Figures S1–S6 [file mmc1.pdf]

Cell Reports
Supplemental Information

Global Reprogramming of Host SUMOylation during Influenza Virus Infection

Patricia Domingues, Filip Golebiowski, Michael H. Tatham, Antonio M. Lopes, Aislynn Taggart, Ronald T. Hay, and Benjamin G. Hale

Figure S1

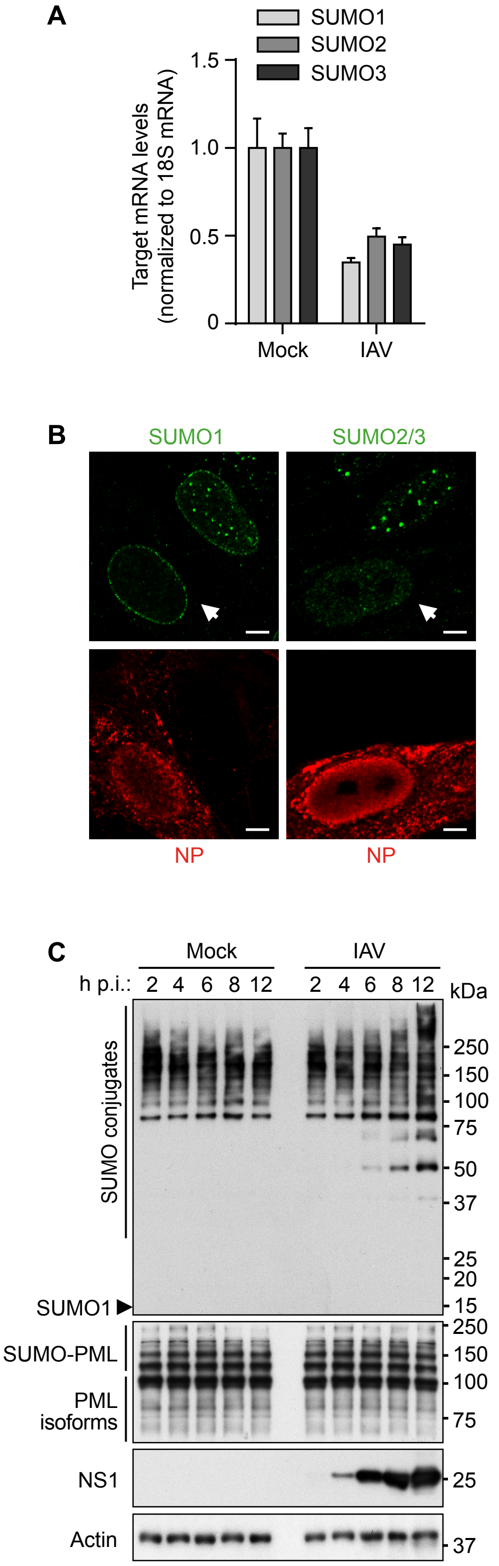
A
SUMO1
SUMO2
SUMO3
Target mRNA levels (normalized to 18S mRNA)
1.5
1.0
0.5
0
Mock
IAV
B
SUMO1
SUMO2/3
NP
NP
C
Mock
IAV
h p.i.: 2 4 6 8 12 2 4 6 8 12
kDa
SUMO conjugates
SUMO1
SUMO-PML
PML isoforms
NS1
Actin
250
150
100
75
50
37
25
20
15
25
37

Figure S1, related to Figure 1. Induction of SUMO conjugates by IAV infection does not correlate with changes in SUMO mRNA levels and shows target specificity. (**A**) RT-qPCR analysis of SUMO paralogue mRNA levels in A549 cells following infection with IAV at an MOI of 5 PFU/cell (or mock). Bars represent mean values, and error bars standard deviation of three independent experiments. (**B**) Immunofluorescent analysis of MRC5 cells infected with IAV at an MOI of 0.1 PFU/cell for 12h. SUMO1, or SUMO2/3, and NP were visualized after staining with specific antibodies. An uninfected and infected cell is shown in each panel for comparison of the 'SUMO foci' and 'SUMO diffuse' phenotypes. Scale bars represent 5μm. (**C**) Western blot analysis of whole-cell lysates from MRC5 cells infected with IAV at an MOI of 5 PFU/cell for the times indicated. SUMO1, PML, NS1, and actin were detected with specific antibodies.

Figure S2

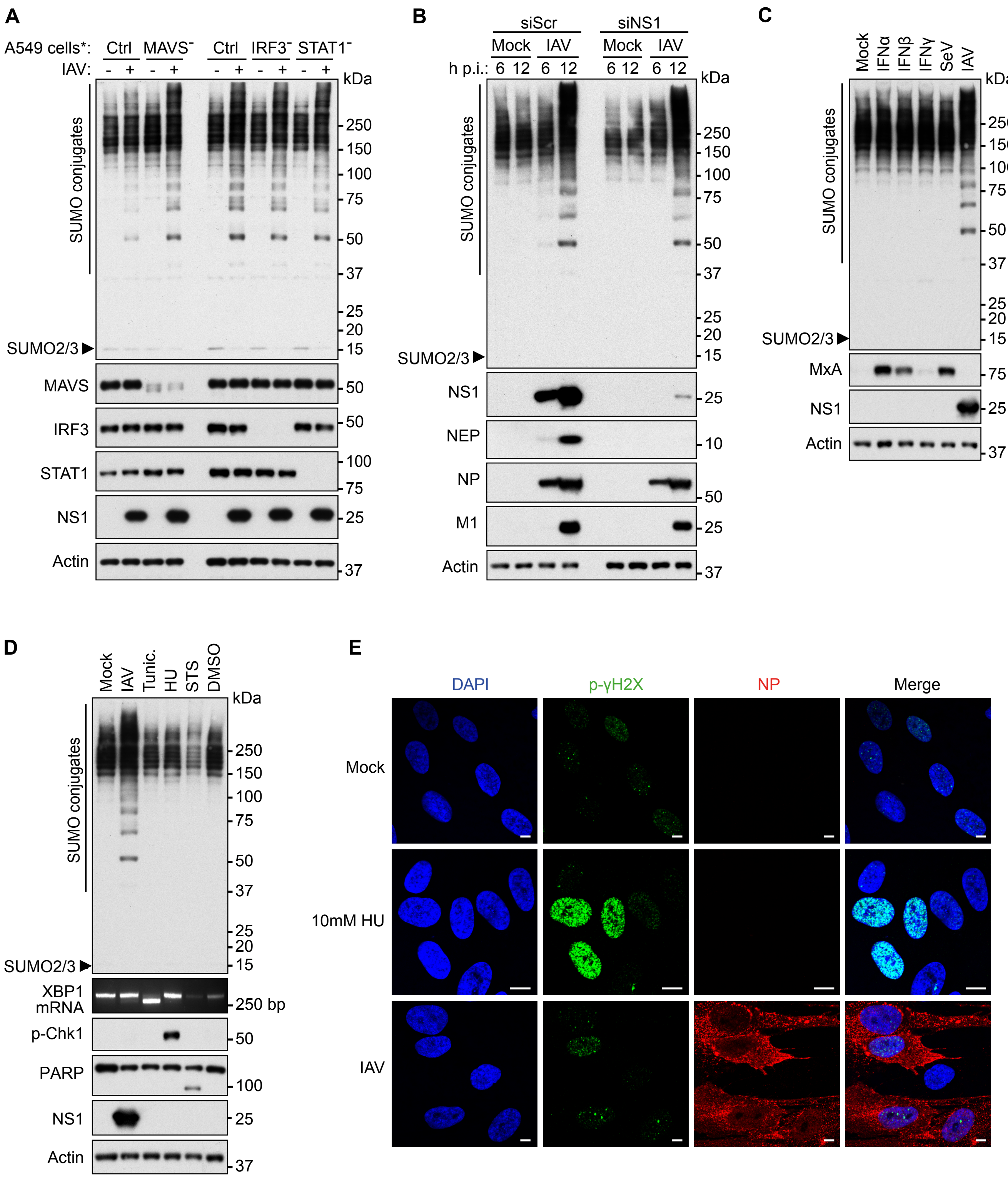
A
A549 cells*: Ctrl MAVS⁻ Ctrl IRF3⁻ STAT1⁻
IAV: - + - + - + - + - +
kDa
SUMO conjugates
SUMO2/3
250 150 100 75 50 37 25 20 15
MAVS
IRF3
STAT1
NS1
Actin
B
siScr siNS1
Mock IAV Mock IAV
h p.i.: 6 12 6 12 6 12 6 12
kDa
SUMO conjugates
SUMO2/3
NS1
NEP
NP
M1
Actin
C
Mock IFNα IFNβ IFNγ SeV IAV
kDa
SUMO conjugates
SUMO2/3
MxA
NS1
Actin
D
Mock IAV Tunic. HU STS DMSO
kDa
SUMO conjugates
SUMO2/3
XBP1 mRNA
250 bp
p-Chk1
PARP
NS1
Actin
E
DAPI
p-γH2X
NP
Merge
Mock
10mM HU
IAV

Figure S2, related to Figure 2. Canonical interferon, ER stress, apoptotic and DNA-damage response pathways do not correlate with IAV-triggered SUMOylation. (**A**) A549 cell-lines stably expressing HCV NS3/4A (cleaves MAVS), BVDV-NPro (degrades IRF3) or PIV5-V (degrades STAT1) were infected with IAV at an MOI of 10 PFU/cell for 18h. Following total-cell lysis, western blot analysis was performed to detect SUMO2/3, MAVS, IRF3, STAT1, NS1 and actin. (**B**) siRNAs targeting NS1/NEP were transiently transfected into A549s 48h prior to infection with IAV at an MOI of 5 PFU/cell for the indicated time. Following total-cell lysis, western blot analysis was performed to detect SUMO2/3, NS1, NEP, NP, M1 and actin. (**C**) A549 cells were treated with 200IU/ml IFNα, IFNβ or IFNγ, or infected with SeV or IAV at an MOI of 10 PFU/ml, for 18h. Following total-cell lysis, western blot analysis was performed to detect SUMO2/3, MxA, NS1 and actin. (**D**) Impact of stimulating ER stress, apoptosis and DNA-damage on SUMOylation. A549 cells were infected with IAV at an MOI of 5 PFU/cell or treated with different drug compounds to activate ER stress (Tunicamycin, Tunic.; 1μg/ml), DNA damage (Hydroxyurea, HU; 10mM) or apoptosis (Staurosporine, STS; 1μM). At 12h post infection/treatment, total cell lysates or total RNA were analyzed by western blot to detect SUMO2/3, phospho-Chk1 (marker for DNA damage), PARP (PARP cleavage marker of apoptosis), NS1, or actin protein levels, or RT-PCR to detect XBP1 splicing (marker of ER stress). (**E**) Immunofluorescent analysis of MRC5 cells stimulated with 10mM hydroxyurea for 12h, or infected with IAV for 12h. Cells were stained for phospho-histone H2AX (marker for DNA damage), NP and DNA (DAPI). Scale bars represent 10μm.

Figure S3

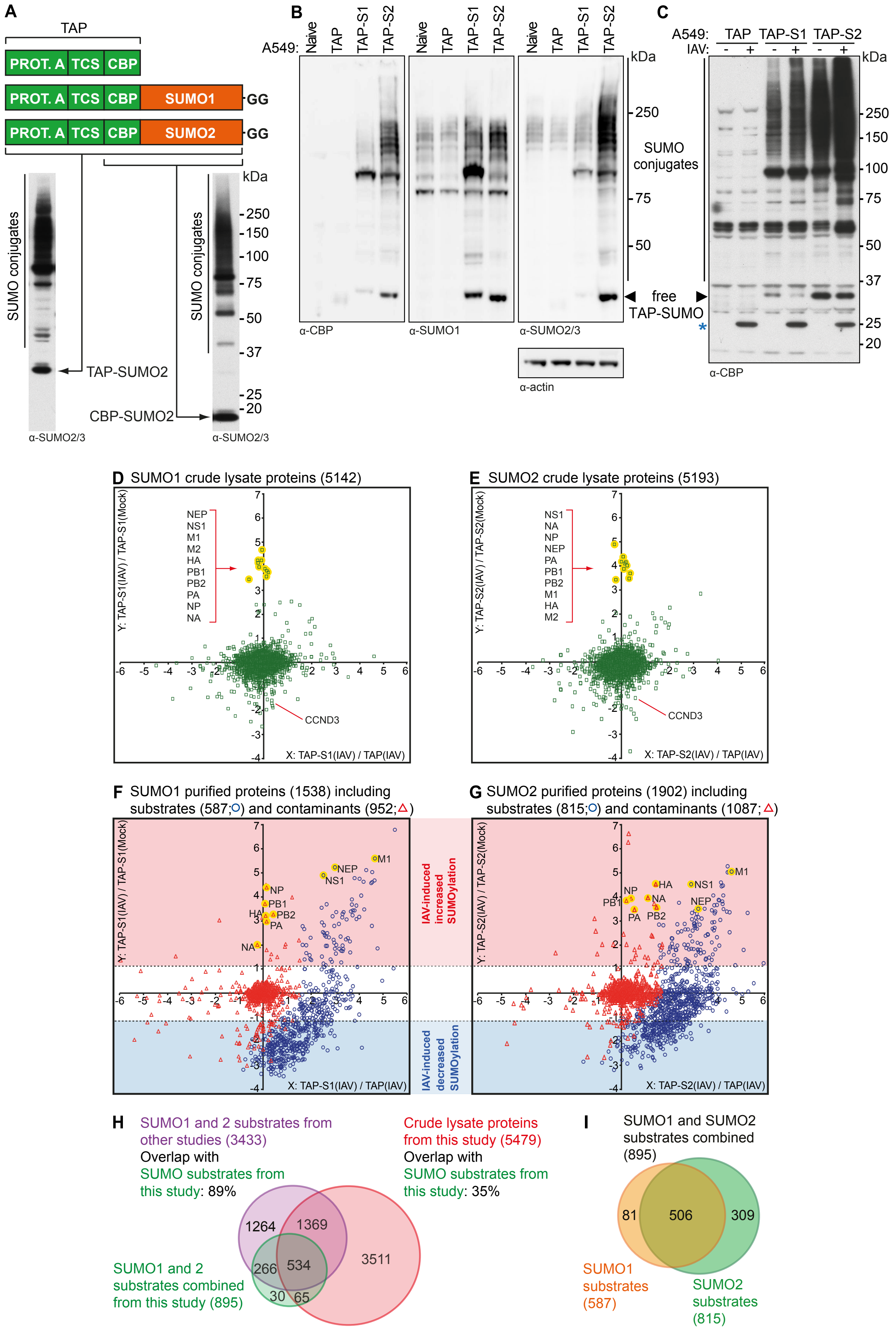
A
TAP
PROT. A TCS CBP
SUMO1 GG
SUMO2 GG
SUMO conjugates
TAP-SUMO2
CBP-SUMO2
α-SUMO2/3
B
A549: Naive TAP TAP-S1 TAP-S2
α-CBP
α-SUMO1
α-SUMO2/3
α-actin
SUMO conjugates
free TAP-SUMO
C
A549: TAP TAP-S1 TAP-S2
IAV: - + - + - +
α-CBP
kDa
D SUMO1 crude lysate proteins (5142)
Y: TAP-S1(IAV) / TAP-S1(Mock)
X: TAP-S1(IAV) / TAP(IAV)
NEP NS1 M1 M2 HA PB1 PB2 PA NP NA
CCND3
E SUMO2 crude lysate proteins (5193)
Y: TAP-S2(IAV) / TAP-S2(Mock)
X: TAP-S2(IAV) / TAP(IAV)
NS1 NA NP NEP PA PB1 PB2 M1 HA M2
CCND3
F SUMO1 purified proteins (1538) including substrates (587;○) and contaminants (952;△)
G SUMO2 purified proteins (1902) including substrates (815;○) and contaminants (1087;△)
IAV-induced increased SUMOylation
IAV-induced decreased SUMOylation
M1 NEP NS1 NP PB1 HA PB2 PA NA
H
SUMO1 and 2 substrates from other studies (3433)
Overlap with SUMO substrates from this study: 89%
Crude lysate proteins from this study (5479)
Overlap with SUMO substrates from this study: 35%
1264 1369 266 534 3511 30 65
SUMO1 and 2 substrates combined from this study (895)
I
SUMO1 and SUMO2 substrates combined (895)
81 506 309
SUMO1 substrates (587)
SUMO2 substrates (815)

Figure S3, related to Figure 3. Quantitative SUMO proteomics of IAV-infected cells. (**A-C**) Generation and characterization of A549 cells stably expressing TAP, TAP-SUMO1 or TAP-SUMO2. (**A**) Schematic representation of the TAP tag and the TAP-SUMO constructs, together with representative western blots (anti-SUMO2/3) of total-cell lysate from cells stably expressing TAP-SUMO2 (left panel) and TAP-SUMO2 purified material (right panel) to indicate motility of protein constructs and conjugates before and after TEV cleavage. Abbreviations: PROT. A: protein A; TCS: TEV cleavage site; CBP: calmodulin binding protein. (**B**) Western blot analysis of SUMO1 and SUMO2/3 total protein expression levels in naïve A549 cells and A549s stably expressing TAP only, TAP-SUMO1 or TAP-SUMO2. (**C**) A549 cells stably expressing TAP, TAP-SUMO1 or TAP-SUMO2 were infected (or mock) with IAV at an MOI of 2 PFU/cell for 10h and total-cell lysates were analyzed by western blot with an anti-CBP antibody. Asterisk indicates a non-specific band that is present in infected cell lysates only. (**D-G**) **tsMAPs of all SUMO proteomics data.** (**D & E**) tsMAPs of normalized crude sample data for TAP-SUMO1 (**D**) and TAP-SUMO2 (**E**) experiments as detailed in figure 3 indicating log2-fold changes in total protein abundance following IAV infection (y-axis), or between the TAP- and TAP-SUMO A549 cell lines (x-axis). IAV proteins are highlighted with yellow background and named, as is cyclin D3 (CCND3), a host protein previously described to decrease in abundance during IAV infection (Zhang et al., 2011). (**F & G**) tsMAPs of normalized purified sample data for TAP-SUMO1 (**F**) and TAP-SUMO2 (**G**) experiments indicating log2-fold changes in protein modification following IAV infection (y-axis), or between the TAP- and TAP-SUMO A549 cell-lines (x-axis). Contaminant proteins (e.g. non-specifically bound proteins from all conditions, or external contaminants, such as keratins) are indicated with red triangles and SUMO

substrates with blue circles. IAV proteins are highlighted with yellow background and named. (**H**) Venn diagram showing high overlap in the SUMO substrates identified in this study with those identified in other studies, and low overlap with proteins identified in general crude lysates, suggesting high enrichment of SUMO conjugates. (**I**) Venn diagram showing the overlap in SUMO substrates identified in the two independent SUMO1 and SUMO2 experiments.

Figure S4

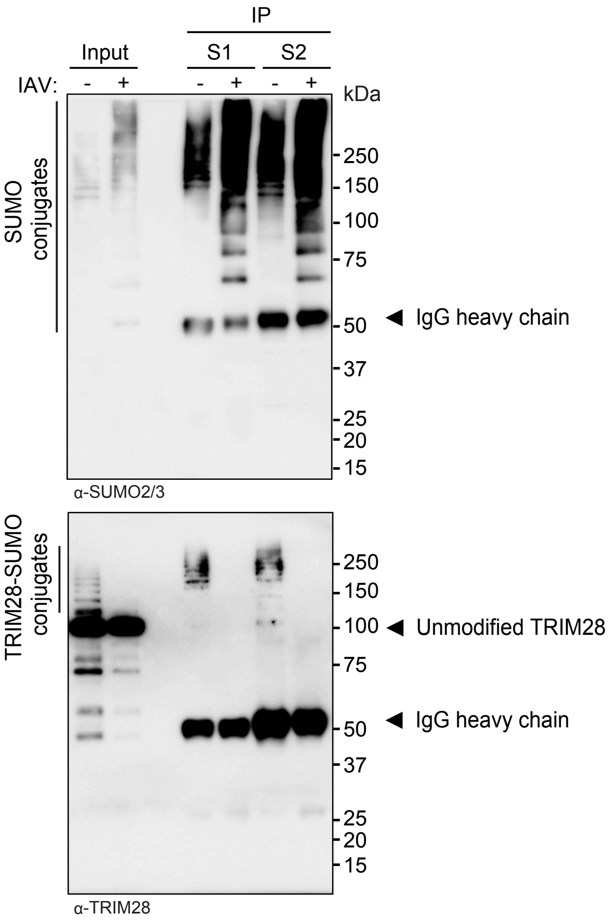
IP
Input
S1
S2
IAV:
- + - + - +
kDa
SUMO conjugates
250
150
100
75
50
37
25
20
15
IgG heavy chain
α-SUMO2/3
TRIM28-SUMO conjugates
250
150
100
75
50
37
25
20
15
Unmodified TRIM28
IgG heavy chain
α-TRIM28

Figure S4, related to Figure 4. Validation of IAV-induced deSUMOylation of endogenous TRIM28. A549 cells were infected with IAV at an MOI of 5 PFU/cell for 16h. Western blot analysis was performed on both total-cell lysates and SUMO immunoprecipitates to detect SUMO2/3 and TRIM28.

Figure S5

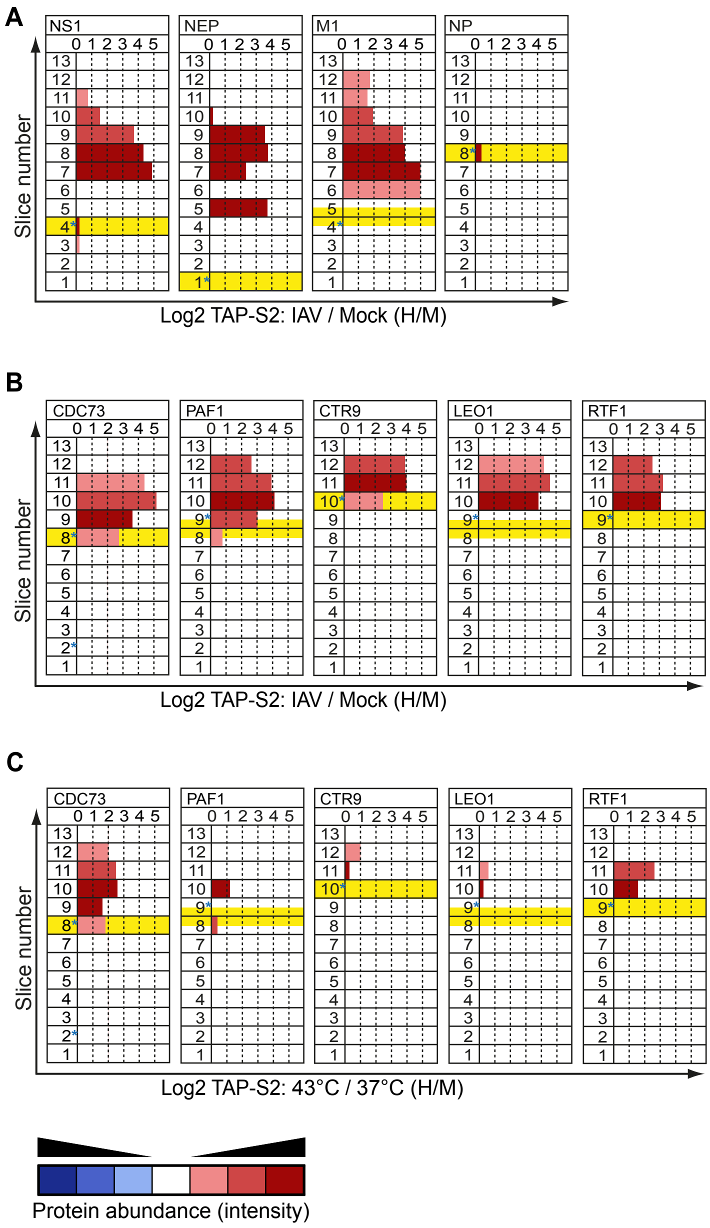
A
NS1
NEP
M1
NP
0 1 2 3 4 5
Slice number
Log2 TAP-S2: IAV / Mock (H/M)
B
CDC73
PAF1
CTR9
LEO1
RTF1
Slice number
Log2 TAP-S2: IAV / Mock (H/M)
C
CDC73
PAF1
CTR9
LEO1
RTF1
Slice number
Log2 TAP-S2: 43°C / 37°C (H/M)
Protein abundance (intensity)

Figure S5, related to Figures 4 & 5. Analysis of electrophoretic mobility shifts as a result of SUMO modification. (A-B) Predicted molecular weight (preMW) of substrates (unmodified) was compared to their observed electrophoretic mobility (obsEM) across all 13 slices (crude and TAP-SUMO2 purified, IAV/Mock). Yellow background indicates slice where unmodified protein would be expected based on its preMW. Bar length indicates change in protein ratio between IAV- and Mock- infected (H/M) purified TAP-SUMO2 samples. Intensity of color indicates protein abundance (intensity) in each slice: red nominally given to increased H/M ratios while blue nominally given to decreased H/M ratios. Asterisks indicate obsEM of specified proteins in crude lysate. (**A**) Analysis of NS1, NEP and M1 viral proteins confirms their annotation as SUMO substrates. NP analysis suggests it is a contaminant. (**B**) Analysis of PAF1 complex proteins in IAV-infected TAP-SUMO2 cells confirms their annotation as SUMO targets with increased modification following infection. (**C**) Analysis of PAF1 complex proteins in heat-shock stimulated TAP-SUMO2 cells – increased SUMOylation of CDC73 and RTF1 can be readily detected.

Figure S6

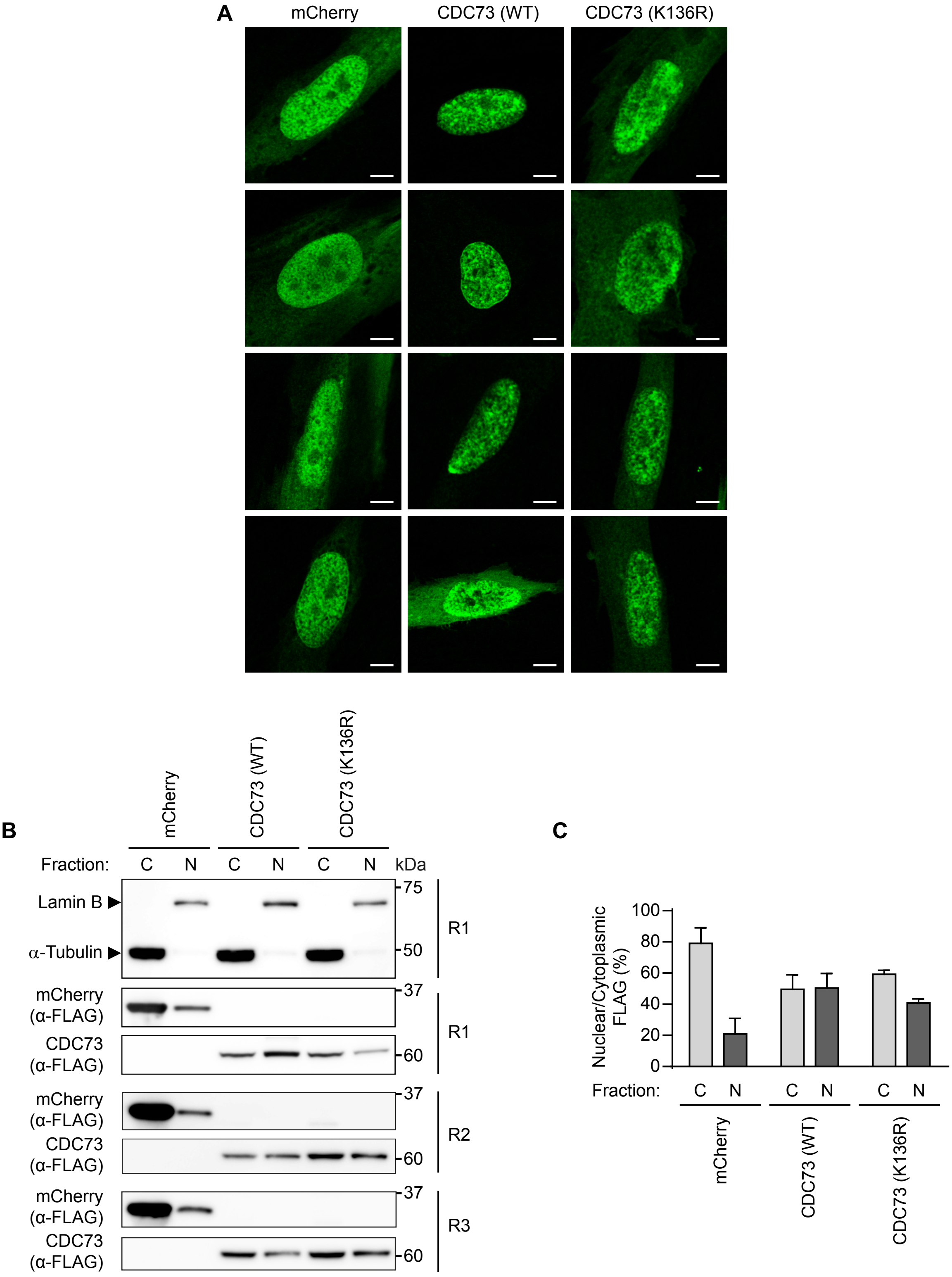
A
mCherry
CDC73 (WT)
CDC73 (K136R)
B
Fraction:
C
N
kDa
Lamin B
α-Tubulin
75
50
37
60
R1
R2
R3
mCherry (α-FLAG)
CDC73 (α-FLAG)
C
Nuclear/Cytoplasmic FLAG (%)
0
20
40
60
80
100

Figure S6, related to Figure 7. Intracellular distribution of CDC73 and CDC73-K136R. (**A**) Immunofluorescent analysis (anti-FLAG) of MRC5 cells transiently expressing FLAG-tagged mCherry, FLAG-tagged CDC73-WT or FLAG-tagged CDC73-K136R. Four representative images are shown. Scale bars represent 5µm. (**B**) Western blot analysis of nuclear (N) and cytoplasmic (C) fractions prepared from 293T cells transiently expressing FLAG-tagged mCherry, FLAG-tagged CDC73-WT or FLAG-tagged CDC73-K136R. Proteins were detected with the indicated antibodies. The experiment was performed three independent times: for the FLAG-tagged proteins of interest, each replicate is shown (R1-R3); for the Lamin B and α-Tubulin controls, a representative image (R1) is shown. (**C**) Quantification of the three independent replicates from (**B**). Bars represent mean values with standard deviations indicated.

Table S1, related to Figure 3. SUMO1 proteomic dataset in uninfected and IAV-infected A549s.

Table S2, related to Figure 3. SUMO2 proteomic dataset in uninfected and IAV-infected A549s.

Table S3, related to Figure 3. Summary of common and unique SUMO targets in A549s, and the impact of IAV infection.

Table S4, related to Figure 3. Summary of common SUMO targets in A549s and the overlap with previous SUMO site-specific proteomic mapping studies.

Table S5, related to Figure 5. SUMO2 proteomic dataset in unstimulated and heat-shocked A549s.

Table S6, related to Figure 6. Primary shRNA screening data and validations for the role of host SUMO targets during IAV infection.

Table S7, related to Figure 3. Enrichr pathway analysis for functional classification of host substrates that change in SUMOylation during IAV infection and other stresses.

SUPPLEMENTAL EXPERIMENTAL PROCEDURES

Cell-Lines and Compounds.

To generate A549 cells stably expressing TAP or TAP-SUMO proteins, sequences encoding TAP, TAP-SUMO1 or TAP-SUMO2 (ending in the C-terminal GG residues required for conjugation) were subcloned into the *Xho*I and *Xba*I sites of pEFIRES-P (Hobbs et al., 1998) to create pEFIRES-P/TAP, pEFIRES-P/TAP-SUMO1 or pEFIRES/TAP-SUMO2 expression vectors, respectively. Each construct was transfected into A549 cells, and individual clones stably expressing the desired proteins were selected using puromycin (1μg/ml) and validated by western blot. A549 cells stably expressing BVDV-NPro, PIV5-V or HCV-NS3/4A have been described previously (Chen et al., 2010; Hale et al., 2009; Killip et al., 2013), and were generously provided by Rick Randall (University of St. Andrews, UK). Compounds used include: cycloheximide (Sigma, 50μg/ml), leptomycin B (Enzo Life Sciences, 11nM), zanamivir (Redx Pharma, 10μM), tunicamycin (Sigma, 1μg/ml), hydroxyurea (Sigma, 10mM), and staurosporine (Sigma, 1μM). Interferon (IFN) alpha, beta and gamma were purchased from Merck, Calbiochem and Roche, respectively, and used as indicated. TNFα was purchased from Prepotech and used at 10ng/ml. Cell viability was assessed using CellTiter-Glo® Luminescent Cell Viability Assay (Promega), according to the manufacturer's instructions. Nuclear-cytoplasmic fractionation was performed essentially as described (Sunters et al., 2010; Trilling et al., 2014).

Immunofluorescence.

Cells on 13 mm coverslips were fixed, permeabilized and stained as described (Boutell et al., 2011). Antibodies used are detailed below. DNA was stained using DAPI. Images were visualized on a Zeiss LSM 710 confocal microscope. For the mini-replicon reporter assay, pPolI-358-FFluc and pPolI-358-mCherry were generated in a manner similar to previous descriptions (Hoffmann et al., 2008), and pCAGGS expression vectors encoding WSN PA, PB1, PB2 and NP, as well as pDZ-NP(WSN), have been described (Quinlivan et al., 2005; Zhang et al., 2012). pCAGGS expression vectors encoding KAN-1 PB1, PB1-E445A/E446A, AvianPr-PB2-E627K, PA and NP were kindly provided by Martin Schwemmle (University of Freiburg, Germany) (Manz et al., 2012). pmCherry-C1 was purchased from Clontech. Transfections were performed using FuGENE HD (Promega), with the ratio PA:PB1:PB2:NP:reporter being 1:1:1:10:10.

Antibodies.

Antibodies used for western blotting were: SUMO1 (Abcam, ab32058), SUMO2/3 (Abcam, ab53194), TRIM28 (Bethyl, A300-274A), CDC73 (Abcam, ab70533), UBTF (Santa Cruz, sc9131), ATRX (Santa Cruz, sc-15408), RanGAP1 (Life Technologies, 19C7), PML (Bethyl, A301-167A), CBP (Millipore, 07-482), actin (Sigma, A2103), IAV-NS1 (rabbit 1-73 (Solorzano et al., 2005)), IBV-NP (Abcam, ab20711), IAV-M1 (Abcam, ab22396), LACV-N (Reichelt et al., 2004), VSV-N (polyclonal anti-serum a gift from Jovan Pavlovic, University of Zurich, Switzerland), SFV-C (Landis et al., 1998), IAV-NP (Solorzano et al., 2005), FLAG (Sigma, F1804), RIG-I (Baum et al., 2010), NEP (Genescript, A01499), IRF3 (Santa Cruz, sc-9082), STAT1 (Santa Cruz, sc-417), MAVS (a gift from John McLauchlan, University of Glasgow, UK), phospho-Chk1 (Cell Signaling, 2348), PARP (Cell Signaling, 9542), MxA (Santa Cruz, sc-50509), 6His

(Abcam, ab18184), α-tubulin (Sigma, T6074) and Lamin B (Santa Cruz, sc-6216).

Antibodies used for immunofluorescence were: IAV-NP (HT103 (O'Neill et al., 1998)), IAV-PB1 (cc11, a gift from Silke Stertz), IAV-PB2 (polyclonal anti-serum a gift from Silke Stertz), IAV-PA (1J6, a gift from Silke Stertz), PML (Bethyl, A301-167A), hDaxx (Upstate, 07-471), Sp100 (a gift from Roger D. Everett, University of Glasgow, UK), SUMO1 (Enzo Lifesciences, BML-PW0505), SUMO2 (Enzo Lifesciences, BML-PW0510), FLAG (Sigma, F1804), phospho-histone H2AX (Cell Signaling, 9718).

SILAC Cell Culture.

Each experiment consisted of three SILAC conditions: L (light), where A549/TAP cells were grown in DMEM containing isotopically 'normal' amino acids (L-lysine, L-arginine); M (medium), where A549/TAP-SUMO1 (or SUMO2) cells were grown in DMEM containing 4,4,5,5-D_4 lysine and $^{13}C_6$ arginine; and H (heavy), where A549/TAP-SUMO1 (or SUMO2) cells were grown in DMEM containing $^{13}C_6$ $^{15}N_2$ lysine and $^{13}C_6$ $^{15}N_4$ arginine. Cells were grown in 15cm Petri dishes, with 20 dishes per SILAC condition. In both SUMO1 and SUMO2 IAV experiments, cells in condition L and H were infected with IAV for 10h at an MOI of 2 PFU/cell, and cells in condition M were mock-infected. In the SUMO2 heat-shock experiment, cells in condition L and M were maintained at 37°C, while cells in condition H were subjected to heat-shock (43°C) for 30 minutes.

TAP Procedure and Mass Spectrometry.

Lysates were diluted 25x in order to dilute out the denaturing 2% SDS and passed over IgG sepharose (GE Healthcare), which was followed by enzymatic removal of the Protein A portion of the TAP-tag (see **Fig. S3A**) using TEV protease (Promega). The resulting eluate was then affinity purified on calmodulin sepharose (GE Healthcare) followed by protein elution with buffer containing 10 mM EGTA, and protein recovery by precipitation with 100% TCA (w/v) and acetone washing. Purification resulted in ~25μg of protein sample that was resuspended in 30μl of 2x LDS sample buffer (Invitrogen). Crude sample (~50μg) was also mixed 1:1 with 2x LDS sample buffer. Both purified and crude samples were resolved on NuPAGE Novex 10% Bis-Tris polyacrylamide gels using MOPS buffer (Invitrogen). Gel-fractionated proteins were stained with Coommassie blue and the gel was sliced into 13 sections as outlined in **Fig. 3**. Protein slices were subjected to in-gel digestion with trypsin (Promega) essentially as described previously (Shevchenko et al., 2006). The resulting peptide mixtures were vacuum-dried and resuspended in 30μl of 1% formic acid prior to analysis by LC-MS/MS on a Q Exactive mass spectrometer (Thermo Scientific) coupled to an EASY-nLC 1000 liquid chromatography system via an EASY-Spray ion source (Thermo Scientific) running at 75 μm x 500 mm at 45ºC on an EASY-Spray column. An elution gradient duration of 240 min was used. Data were acquired in the data-dependent mode. Full scan spectra (*m/z* 300-1800) were acquired with resolution *R* = 70,000 at *m/z* 400 (after accumulation to a target value of 1,000,000 with maximum injection time of 20 ms). The 10 most intense ions were fragmented by HCD and measured with a target value of 500,000, maximum injection time of 60 ms and intensity threshold of 1.7e3. A 40 second dynamic exclusion list was applied.

Quantitative Mass Spectrometry Data Analyses.

MaxQuant Analyses.

All raw files generated by MS analysis were processed with MaxQuant software (version 1.3.0.5) (Cox and Mann, 2008) and searched against a FASTA database consisting of UniProtKB human and influenza A virus (A/WSN/1933(H1N1)) reference proteomes (both current as of June 2013). Two separate MaxQuant analyses were performed for each of the two IAV experiments. The first analysis aimed to determine relative changes to SUMOylation of all substrates ('global') - this included the data from all 13 TAP-purified sample slices and all 13 corresponding crude-lysate sample slices. The second analysis ('slice-by-slice') was designed to evaluate changes to SUMOylation in each individual sample slice, thus allowing definition of abundance (and thereby ratio) of every protein within the slice. The MaxQuant parameters used are below (asterisks denote non-default settings):

Parameter	Value
Version	1.3.0.5
Fixed modifications	Carbamidomethyl (C)
Randomize	FALSE
Special AAs	KR
Include contaminants	TRUE
MS/MS tol. (FTMS)	20 ppm
Top MS/MS peaks per 100 Da. (FTMS)	10
MS/MS deisotoping (FTMS)	TRUE
MS/MS tol. (ITMS)	0.5 Da
Top MS/MS peaks per 100 Da. (ITMS)	6
MS/MS deisotoping (ITMS)	FALSE
MS/MS tol. (TOF)	0.1 Da
Top MS/MS peaks per 100 Da. (TOF)	10
MS/MS deisotoping (TOF)	TRUE

MS/MS tol. (Unknown)	0.5 Da
Top MS/MS peaks per 100 Da. (Unknown)	6
MS/MS deisotoping (Unknown)	FALSE
Peptide FDR	0.01
Max. peptide PEP	1
Protein FDR	0.01
Site FDR	0.01
Use Normalized Ratios For Occupancy	TRUE
Apply site FDR separately	TRUE
Min. peptide Length	7
Min. score	0
Min. unique peptides	0
Min. razor peptides	1
Min. peptides	1
Use only unmodified peptides and	TRUE
Modifications included in protein quantification	Oxidation (M);Acetyl (N-term);GlyGly (K)
Peptides used for protein quantification	Razor
Discard unmodified counterpart peptides	TRUE
Min. ratio count	2
Lfq min. ratio count	2
Site quantification	Use least modified peptide
Re-quantify	TRUE*
Keep low-scoring versions of identified peptides	No
MS/MS recalibration	FALSE
Match between runs	TRUE*
Time window [min]	2*
Find dependent peptides	FALSE
Labeled amino acid filtering	TRUE
Site tables	GlyGly (K)Sites.txt*;Oxidation (M)Sites.txt
Cut peaks	TRUE
Randomize	FALSE
Special AAs	KR
Include contaminants	TRUE
RT shift	FALSE
Advanced ratios	FALSE
AIF correlation	0.8
First pass AIF correlation	0.8
AIF topx	50
AIF min mass	0
AIF SIL weight	4
AIF ISO weight	2

AIF iterative	FALSE
AIF threshold FDR	0.01

Manual Data Processing of 'Global' Analyses.

Unfiltered datasets resulting from MaxQuant analyses were handled in the form of Excel spreadsheets, and included three SILAC ratios (M/L, H/L and H/M) reflecting the relative abundance of proteins in the three different experimental conditions. As detailed below, data were manually filtered in order to remove contaminants, normalize SILAC ratios, and to define SUMO substrates as well as changes in substrate SUMOylation in response to treatment.

First, all MaxQuant-defined unwanted hits (e.g. 'reverse' (peptide sequences that would match other sequences if reversed), 'contaminants', and 'identified by site' (only identified by modification site)) as well as any remaining suspected internal contaminants (e.g. keratins, immunoglobulins, non-human origin proteins) were removed. All such pre-filtered content was copied into 2 identical tabs and named 'Crude' and 'Purified'. In the 'Crude' list, all hits with 0 unique crude peptides and/or no crude ratios reported were removed. In the 'Purified' list, all hits with 0 unique purified peptides and/or no purified ratios reported were removed. Although MaxQuant uses built-in normalization algorithms to account for variable isotope purity/incorporation or error in lysate mixing, it is only applicable to values distributed in a unimodal Gaussian manner. We therefore applied an alternative method of normalization for our 'purified' data, which due to their highly purified nature and substantial changes in SUMOylation means they are not unimodal. Given that the majority of proteins from our crude samples were unaffected by any

treatment (**Figs. S3D & S3E**), the median of the raw M/L, H/L, and H/M ratios for proteins was calculated and applied to normalize the raw ratios from crude samples as well as from corresponding TAP-purified samples. In order to calculate normalized values, raw ratios were divided by the normalization factors below:

Experiment	Norm. factor M/L	Norm. factor H/L	Norm. factor H/M	Filter cut-off value M/L	Filter cut-off value H/L	Filter cut-off value H/M
IAV SUMO1	1.05	0.95	0.9	1.75	1.58	1.08
IAV SUMO2	0.99	0.92	0.92	1.61	1.54	1.12
HS SUMO2	0.87	0.87	1.0	1.63	1.82	0.82

Next, log2 values of normalized ratios were calculated to facilitate further graphical representation of data in the form of tsMAPs (triple SILAC maps). We assumed that total abundance of the majority of the ~5000 proteins identified in crude lysates does not change significantly with treatment. Thus, the variable distribution of SILAC ratios in the 'crude' samples was deemed representative of the distribution of 'contaminants' in the 'purified' samples. We calculated the total abundance change of the 99% of all proteins closest to zero (log2 values) for all three SILAC ratios in our crude samples (see **Figs. S3D & S3E**), and used the maximum abundance change for these 99% of proteins as

‘cut-off values’ (table above) for filtering the data from the ‘purified’ dataset (see **Figs S3F & S3G**). Further filtering of ‘purified’ ratios was divided into two phases. Firstly, putative SUMO substrates were defined by filtering ratios of TAP-SUMO over TAP and thus applying appropriate cut-off values to M/L and H/L ratios. All ‘hits’ with ratios above certain values (see table above) for either M/L or H/L were called SUMO substrates, and the remainder classified as contaminants. Secondly, changes in substrate SUMO modification by particular treatments were defined by filtering ratios of TAP-SUMO (treatment) over TAP-SUMO (no treatment) and applying appropriate cut-off values to the H/M ratios (table above). All hits with ratios above a certain value (on the positive part of the axis) were defined as substrates with increased SUMOylation in response to treatment, while ‘hits’ below a certain value (on the negative part of the axis) were defined as substrates with decreased SUMOylation (values stated in table above).

A search for ubiquitylation sites (GlyGly) was also included in the processing of the raw mass spectrometry data from purified samples. However, very few (~20) ubiquitylation sites were identified (**Tables S1 & S2**), none of which were subsequently classified as SUMO substrates that change in abundance with IAV infection, and therefore were not followed-up in this study. Notably however, within our A549 SUMO-modified proteome, we identified ubiquitylation sites on ubiquitin itself at lysines 48 and 63. These sites may represent certain topologies of hybrid SUMO-ubiquitin chains (Praefcke et al., 2012).

Manual Data Processing of ‘Slice-by-Slice’ Analyses to Confirm SUMO Modification.

For selected putative SUMO substrates, predicted molecular weight (preMW) was compared to their observed electrophoretic mobility (obsEM) in both ‘crude’ and

‘purified’ datasets. Such a method has been developed previously to confirm ubiquitin conjugation to target proteins (Peng and Cheng, 2005). We assumed that a protein should run according mostly to its mass by SDS-PAGE, and should thus be detected within the gel slice (1-13) covering its approximate MW. For almost all proteins in crude lysates, the preMW was found to correspond to its obsEM when individual protein abundance in each gel slice was analyzed. However, *bona fide* covalent SUMO substrates would have an increased MW, and should therefore be detected in slices corresponding to larger masses. The more highly modified the substrate, the larger the obsEM/preMW ratio should be. This would be most apparent in TAP-SUMO-purified samples given that SUMO is usually only attached to a small proportion of the total population of a protein. Thus, for selected substrates, protein intensity values in individual gel slices from TAP-SUMO-purified material were compared between SILAC conditions. Raw intensity values for M and H data were normalized with the M/L and H/L normalization factors, respectively (table above). All three SILAC ratios were normalized as described for ‘global’ analyses. Among all tested proteins with sufficient data, obsEM/preMW ratios were close to 1 for >95% of crude and contaminant proteins (no SUMO modification), while ratios for putative SUMO substrates were usually infinitely high (no protein observed at its preMW) for >95% of putative SUMO substrates. Protein intensities (abundance) and SILAC ratios within each slice were taken into account when creating graphical representations of these data as shown in **Figs. 4B** and **S5**.

Data Comparisons with Other Studies.

To compare SUMOylation responses between stresses, H/M ratios (i.e. treatment vs non-treatment) for SUMO substrates common to two different datasets were visualized on

tsMAPs with Pearson's coefficient values indicated (**Fig. 5**). All datasets were generated using the same or very similar experimental and data processing methodology (Fritah et al., 2014; Golebiowski et al., 2009; Tatham et al., 2011; Yin et al., 2012). Comparison analyses were made with IPA (Ingenuity Pathway Analysis) software by matching respective gene names and ratios. To assess the reliability of our data, we also compared the substrates identified from the combined SUMO1 and SUMO2 IAV experiments in this study with either the respective crude lysate proteomes (this study) or lists of identified SUMO substrates from other studies (we first generated a database of SUMO substrates defined by publically-available proteomic studies either from our own laboratories or from independent groups (Barysch et al., 2014; Blomster et al., 2009; Fritah et al., 2014; Ganesan et al., 2007; Golebiowski et al., 2009; Hendriks et al., 2014; Matafora et al., 2009; Pungaliya et al., 2007; Rosas-Acosta et al., 2005; Schimmel et al., 2008; Tammsalu et al., 2014; Tatham et al., 2011; Vertegaal et al., 2006)). Comparisons were performed and output collated from IPA software prior to results being depicted as Venn diagrams (**Figure S3H-I**).

PCR-Based Analyses.

RT-qPCR.

Total RNA was isolated using the RNAeasy Plus Kit (Qiagen). RT-qPCRs were performed as a two-step process, and each sample was normalized to an endogenous control (18S rRNA or GAPDH). Complementary DNA (cDNA) was generated by reverse transcription of total RNA using TaqMan Reverse Transcription Reagents (Life Technologies) with an oligo dT primer. Reverse transcription reactions were as follows: (i) primer annealing at 25°C for 10 min, (ii) strand elongation at 37°C for 1h, and (iii)

reverse transcriptase inactivation at 95°C for 5 min. For the second step, sample cDNAs were analyzed in triplicate using the TaqMan Fast Universal PCR Master Mix system (Life Technologies). Each sample was run as a singleplex reaction mixture containing the appropriate TaqMan probe-primer mix: PAF1 (assay ID Hs00219496_m1); C18orf25 (assay ID Hs00973951_g1); AFF4 (assay ID Hs00232683_m1); SUMO1 (assay ID Hs00830844_g1); SUMO2 (assay ID Hs02743873_g1); SUMO3 (assay ID Hs00739248_m1). Probe-primer mixes (FAM/MGB probes) and pre-validated 18S rRNA endogenous control (accession number X03205.1) were purchased from Life Technologies. Real-time reactions were performed in a 7500 Fast Real-Time PCR machine (Life Technologies). Reactions were heated to 95°C for 20s to allow denaturation of the cDNA. PCR was performed as follows: (i) dsDNA strand-separation at 95°C for 3 s; (ii) primer annealing and strand elongation at 60°C for 30s. Steps (i) and (ii) were repeated 40 times. Data were analyzed using 7500 Fast system software (v2.0.5; Life Technologies).

CDC73 and ISG15 mRNA levels (see **Figure 7A**) were determined using the Sybr green RT-qPCR methodology as described previously (Lanz et al., 2015). CDC73-specific primers (5-ACCATTTGCCTTGAACCTTG and 5-GGCCTAAACGTTCACCAAAA) and ISG15-specific primers (5-TGTCGGTGTCAGAGCTGAAG and 5-GCCCTTGTTATTCCTCACCA) were used.

Xbp1 Splicing Assay.

XBP1 mRNA levels were determined using the One-Step AccessQuick RT-PCR system (Promega). Briefly, 1μg of total RNA was reverse-transcribed with AMV Reverse

Transcriptase (AMV RT) and the cDNA amplified with *Tfl* DNA polymerase using XBP1 specific primers (5-AGTGGCCGGGTCTGCTGAGT and 5-GGCTTCCAGCTTGGCTGATGACG). Following separation on a 1% agarose gel, the RT-PCR products corresponding to the unspliced and spliced (26 nucleotide deletion) forms of XBP1 mRNA were visualized by ethidium bromide staining.

RNA Interference.

Cells were transfected in suspension with siRNA duplexes targeting NS1/NEP (AACGGAGGACTTGAATGGAAT), CDC73 (4 individual siRNAs from FlexiTube GeneSolution GS79577, Qiagen), or a control siRNA duplex (Allstars Negative Control siRNA, SI03650318, Qiagen) at a final concentration of 30nM, using Lipofectamine RNAiMAX (Life Technologies). 48h post-transfection, cells were treated as indicated.

Luciferase Reporter Assays.

Transfections were performed in 24- or 96- well plates using Fugene HD (Promega) or Lipofectamine 3000 (Life Technologies). pGL3-Mx1P-FFluc (ISRE-containing promoter), pNFκB-FFLuc (NFκB promoter) and p125-FFLuc (IFNβ promoter) have been described previously (Kochs et al., 2007). pRL-SV40 (Promega) was used as an internal transfection/normalization control. Expression plasmids for GST and PIV5-V have been described previously (Hale et al., 2010; Kochs et al., 2007). FLAG-SENP2 was a gift from Edward Yeh (Addgene plasmid #18047)(Kang et al., 2010). The nucleotide sequences of CDC73 or mCherry were PCR amplified from existing pcDNA3-HRPT2 (gift from Matthew Meyerson (Addgene plasmid #11048)(Rozenblatt-Rosen et al., 2005)) or pmCherry-C1 vectors, respectively, and ligated in-frame into p3xFLAG-CMV-7.1

(Sigma-Aldrich) so as to express with N-terminal FLAG tags. Indicated CDC73 mutants were generated by Quikchange II Site-Directed Mutagenesis (Agilent Technologies) according to the manufacturer's instructions. Newly-generated constructs were authenticated by DNA sequencing.

Detection of Endogenous and Exogenous SUMOylated Proteins.

For detection of SUMOylated overexpressed CDC73, FLAG-tagged CDC73 constructs were co-transfected into 293T cells with pCAGGS expression vectors encoding 6His-tagged SUMO2-GG or 6His-tagged SUMO2-AA. Cells were harvested 36h post-transfection, and 6His-tagged SUMO proteins and conjugates were purified under denaturing conditions essentially as described previously (Tatham et al., 2009). To evaluate the SUMOylation status of endogenous proteins, cells were lysed in buffer containing 50mM Tris-HCl (pH 7.8), 650mM NaCl, 1% NP-40, 2% SDS, 5mM EDTA, and freshly supplemented with 20mM iodoacetamide, 10mM β-mercaptoethanol and cOmplete™, Mini, EDTA-free Protease Inhibitor Cocktail tablets (Roche). SDS levels were subsequently diluted to ~1% and β-mercaptoethanol levels to ~5mM. Lysates were then passed through a 29G needle six times. Following centrifugation at 14,000 rpm for 30 minutes at 10°C, soluble fractions were incubated with end-over-end mixing overnight at 4°C with either anti-SUMO1 (Abcam, ab32058) or anti-SUMO2/3 (Abcam, ab53194) antibodies. Immune complexes were precipitated using Protein-G beads and washed extensively in buffer containing 50mM Tris-HCl (pH 7.8), 650mM NaCl, 1% NP-40, 0.1% SDS, 5mM EDTA, and freshly supplemented with 20mM iodoacetamide and cOmplete™, Mini, EDTA-free Protease Inhibitor Cocktail tablets (Roche). Purified

proteins were eluted from the beads using 2X urea disruption buffer (6M urea, 2M β-mercaptoethanol, 4% SDS), and proteins of interest were detected by western blotting.

Statistical Analyses.

Statistical analyses were performed using an unpaired two-tailed Student's t-test. The *p* values for significance are stated in the figure legends.

Bioinformatic Pathway Analyses.

Bioinformatic pathway analyses were performed using the Enrichr platform (Chen et al., 2013).

SUPPLEMENTAL REFERENCES

Barysch, S.V., Dittner, C., Flotho, A., Becker, J., and Melchior, F. (2014). Identification and analysis of endogenous SUMO1 and SUMO2/3 targets in mammalian cells and tissues using monoclonal antibodies. Nature protocols *9*, 896-909.

Baum, A., Sachidanandam, R., and Garcia-Sastre, A. (2010). Preference of RIG-I for short viral RNA molecules in infected cells revealed by next-generation sequencing. Proceedings of the National Academy of Sciences of the United States of America *107*, 16303-16308.

Blomster, H.A., Hietakangas, V., Wu, J., Kouvonen, P., Hautaniemi, S., and Sistonen, L. (2009). Novel proteomics strategy brings insight into the prevalence of SUMO-2 target sites. Molecular & cellular proteomics : MCP *8*, 1382-1390.

Boutell, C., Cuchet-Lourenco, D., Vanni, E., Orr, A., Glass, M., McFarlane, S., and Everett, R.D. (2011). A viral ubiquitin ligase has substrate preferential SUMO targeted ubiquitin ligase activity that counteracts intrinsic antiviral defence. PLoS pathogens *7*, e1002245.

Chen, E.Y., Tan, C.M., Kou, Y., Duan, Q., Wang, Z., Meirelles, G.V., Clark, N.R., and Ma'ayan, A. (2013). Enrichr: interactive and collaborative HTML5 gene list enrichment analysis tool. BMC bioinformatics *14*, 128.

Chen, S., Short, J.A., Young, D.F., Killip, M.J., Schneider, M., Goodbourn, S., and Randall, R.E. (2010). Heterocellular induction of interferon by negative-sense RNA viruses. Virology *407*, 247-255.

Cox, J., and Mann, M. (2008). MaxQuant enables high peptide identification rates, individualized p.p.b.-range mass accuracies and proteome-wide protein quantification. Nature biotechnology *26*, 1367-1372.

Fritah, S., Lhocine, N., Golebiowski, F., Mounier, J., Andrieux, A., Jouvion, G., Hay, R.T., Sansonetti, P., and Dejean, A. (2014). Sumoylation controls host anti-bacterial response to the gut invasive pathogen Shigella flexneri. EMBO reports *15*, 965-972.

Ganesan, A.K., Kho, Y., Kim, S.C., Chen, Y., Zhao, Y., and White, M.A. (2007). Broad spectrum identification of SUMO substrates in melanoma cells. Proteomics *7*, 2216-2221.

Golebiowski, F., Matic, I., Tatham, M.H., Cole, C., Yin, Y., Nakamura, A., Cox, J., Barton, G.J., Mann, M., and Hay, R.T. (2009). System-wide changes to SUMO modifications in response to heat shock. Science signaling *2*, ra24.

Hale, B.G., Knebel, A., Botting, C.H., Galloway, C.S., Precious, B.L., Jackson, D., Elliott, R.M., and Randall, R.E. (2009). CDK/ERK-mediated phosphorylation of the human influenza A virus NS1 protein at threonine-215. Virology *383*, 6-11.

Hale, B.G., Steel, J., Medina, R.A., Manicassamy, B., Ye, J., Hickman, D., Hai, R., Schmolke, M., Lowen, A.C., Perez, D.R., *et al.* (2010). Inefficient control of host gene expression by the 2009 pandemic H1N1 influenza A virus NS1 protein. Journal of virology *84*, 6909-6922.

Hendriks, I.A., D'Souza, R.C., Yang, B., Verlaan-de Vries, M., Mann, M., and Vertegaal, A.C. (2014). Uncovering global SUMOylation signaling networks in a site-specific manner. Nature structural & molecular biology *21*, 927-936.

Hobbs, S., Jitrapakdee, S., and Wallace, J.C. (1998). Development of a bicistronic vector driven by the human polypeptide chain elongation factor 1alpha promoter for creation of stable mammalian cell lines that express very high levels of recombinant proteins. Biochemical and biophysical research communications *252*, 368-372.

Hoffmann, H.H., Palese, P., and Shaw, M.L. (2008). Modulation of influenza virus replication by alteration of sodium ion transport and protein kinase C activity. Antiviral research *80*, 124-134.

Kang, X., Qi, Y., Zuo, Y., Wang, Q., Zou, Y., Schwartz, R.J., Cheng, J., and Yeh, E.T. (2010). SUMO-specific protease 2 is essential for suppression of polycomb group protein-mediated gene silencing during embryonic development. Molecular cell *38*, 191-201.

Killip, M.J., Young, D.F., Gatherer, D., Ross, C.S., Short, J.A., Davison, A.J., Goodbourn, S., and Randall, R.E. (2013). Deep sequencing analysis of defective genomes of parainfluenza virus 5 and their role in interferon induction. Journal of virology *87*, 4798-4807.

Kochs, G., Garcia-Sastre, A., and Martinez-Sobrido, L. (2007). Multiple anti-interferon actions of the influenza A virus NS1 protein. Journal of virology *81*, 7011-7021.

Landis, H., Simon-Jodicke, A., Kloti, A., Di Paolo, C., Schnorr, J.J., Schneider-Schaulies, S., Hefti, H.P., and Pavlovic, J. (1998). Human MxA protein confers resistance to Semliki Forest virus and inhibits the amplification of a Semliki Forest virus-based replicon in the absence of viral structural proteins. Journal of virology *72*, 1516-1522.

Lanz, C., Yanguez, E., Andenmatten, D., and Stertz, S. (2015). Swine interferon-inducible transmembrane proteins potently inhibit influenza A virus replication. Journal of virology *89*, 863-869.

Manz, B., Brunotte, L., Reuther, P., and Schwemmle, M. (2012). Adaptive mutations in NEP compensate for defective H5N1 RNA replication in cultured human cells. Nature communications *3*, 802.

Matafora, V., D'Amato, A., Mori, S., Blasi, F., and Bachi, A. (2009). Proteomics analysis of nucleolar SUMO-1 target proteins upon proteasome inhibition. Molecular & cellular proteomics : MCP *8*, 2243-2255.

O'Neill, R.E., Talon, J., and Palese, P. (1998). The influenza virus NEP (NS2 protein) mediates the nuclear export of viral ribonucleoproteins. The EMBO journal *17*, 288-296.

Peng, J., and Cheng, D. (2005). Proteomic analysis of ubiquitin conjugates in yeast. Methods in enzymology *399*, 367-381.

Praefcke, G.J., Hofmann, K., and Dohmen, R.J. (2012). SUMO playing tag with ubiquitin. Trends in biochemical sciences *37*, 23-31.

Pungaliya, P., Kulkarni, D., Park, H.J., Marshall, H., Zheng, H., Lackland, H., Saleem, A., and Rubin, E.H. (2007). TOPORS functions as a SUMO-1 E3 ligase for chromatin-modifying proteins. Journal of proteome research *6*, 3918-3923.

Quinlivan, M., Zamarin, D., Garcia-Sastre, A., Cullinane, A., Chambers, T., and Palese, P. (2005). Attenuation of equine influenza viruses through truncations of the NS1 protein. Journal of virology *79*, 8431-8439.

Reichelt, M., Stertz, S., Krijnse-Locker, J., Haller, O., and Kochs, G. (2004). Missorting of LaCrosse virus nucleocapsid protein by the interferon-induced MxA GTPase involves smooth ER membranes. Traffic *5*, 772-784.

Rosas-Acosta, G., Russell, W.K., Deyrieux, A., Russell, D.H., and Wilson, V.G. (2005). A universal strategy for proteomic studies of SUMO and other ubiquitin-like modifiers. Molecular & cellular proteomics : MCP *4*, 56-72.

Rozenblatt-Rosen, O., Hughes, C.M., Nannepaga, S.J., Shanmugam, K.S., Copeland, T.D., Guszczynski, T., Resau, J.H., and Meyerson, M. (2005). The parafibromin tumor suppressor protein is part of a human Paf1 complex. Molecular and cellular biology *25*, 612-620.

Schimmel, J., Larsen, K.M., Matic, I., van Hagen, M., Cox, J., Mann, M., Andersen, J.S., and Vertegaal, A.C. (2008). The ubiquitin-proteasome system is a key component of the SUMO-2/3 cycle. Molecular & cellular proteomics : MCP *7*, 2107-2122.

Shevchenko, A., Tomas, H., Havlis, J., Olsen, J.V., and Mann, M. (2006). In-gel digestion for mass spectrometric characterization of proteins and proteomes. Nature protocols *1*, 2856-2860.

Solorzano, A., Webby, R.J., Lager, K.M., Janke, B.H., Garcia-Sastre, A., and Richt, J.A. (2005). Mutations in the NS1 protein of swine influenza virus impair anti-interferon activity and confer attenuation in pigs. Journal of virology *79*, 7535-7543.

Sunters, A., Armstrong, V.J., Zaman, G., Kypta, R.M., Kawano, Y., Lanyon, L.E., and Price, J.S. (2010). Mechano-transduction in osteoblastic cells involves strain-regulated estrogen receptor alpha-mediated control of insulin-like growth factor (IGF) I receptor sensitivity to Ambient IGF, leading to phosphatidylinositol 3-kinase/AKT-dependent Wnt/LRP5 receptor-independent activation of beta-catenin signaling. The Journal of biological chemistry *285*, 8743-8758.

Tammsalu, T., Matic, I., Jaffray, E.G., Ibrahim, A.F., Tatham, M.H., and Hay, R.T. (2014). Proteome-wide identification of SUMO2 modification sites. Science signaling *7*, rs2.

Tatham, M.H., Matic, I., Mann, M., and Hay, R.T. (2011). Comparative proteomic analysis identifies a role for SUMO in protein quality control. Science signaling *4*, rs4.

Tatham, M.H., Rodriguez, M.S., Xirodimas, D.P., and Hay, R.T. (2009). Detection of protein SUMOylation in vivo. Nature protocols *4*, 1363-1371.

Trilling, M., Le, V.T., Rashidi-Alavijeh, J., Katschinski, B., Scheller, J., Rose-John, S., Androsiac, G.E., Jonjic, S., Poli, V., Pfeffer, K.*, et al.* (2014). "Activated" STAT

proteins: a paradoxical consequence of inhibited JAK-STAT signaling in cytomegalovirus-infected cells. Journal of immunology *192*, 447-458.

Vertegaal, A.C., Andersen, J.S., Ogg, S.C., Hay, R.T., Mann, M., and Lamond, A.I. (2006). Distinct and overlapping sets of SUMO-1 and SUMO-2 target proteins revealed by quantitative proteomics. Molecular & cellular proteomics : MCP *5*, 2298-2310.

Yin, Y., Seifert, A., Chua, J.S., Maure, J.F., Golebiowski, F., and Hay, R.T. (2012). SUMO-targeted ubiquitin E3 ligase RNF4 is required for the response of human cells to DNA damage. Genes & development *26*, 1196-1208.

Zhang, L., Das, P., Schmolke, M., Manicassamy, B., Wang, Y., Deng, X., Cai, L., Tu, B.P., Forst, C.V., Roth, M.G*., et al.* (2012). Inhibition of pyrimidine synthesis reverses viral virulence factor-mediated block of mRNA nuclear export. The Journal of cell biology *196*, 315-326.

Zhang, Y., Kien, F., Ma, H.L., Tse, J., Poon, L.L.M., and Nal, B. (2011). Identification of a novel interaction between the M2 proton channel of influenza A virus and cyclin D3: consequences for cell cycle progression. BMC Proc *5*, 70.
