# Supplementary material for: Global Reprogramming of Host SUMOylation during Influenza Virus Infection
Source: Cell Rep. 2015 Nov 5;13(7):1467–80. doi: 10.1016/j.celrep.2015.10.001 (PMC4660286; doi:10.1016/j.celrep.2015.10.001)
Supplement: Document S2. Article plus Supplemental Information [file mmc9.pdf]

Cell Reports

Resource

Global Reprogramming of Host SUMOylation during Influenza Virus Infection

Graphical Abstract

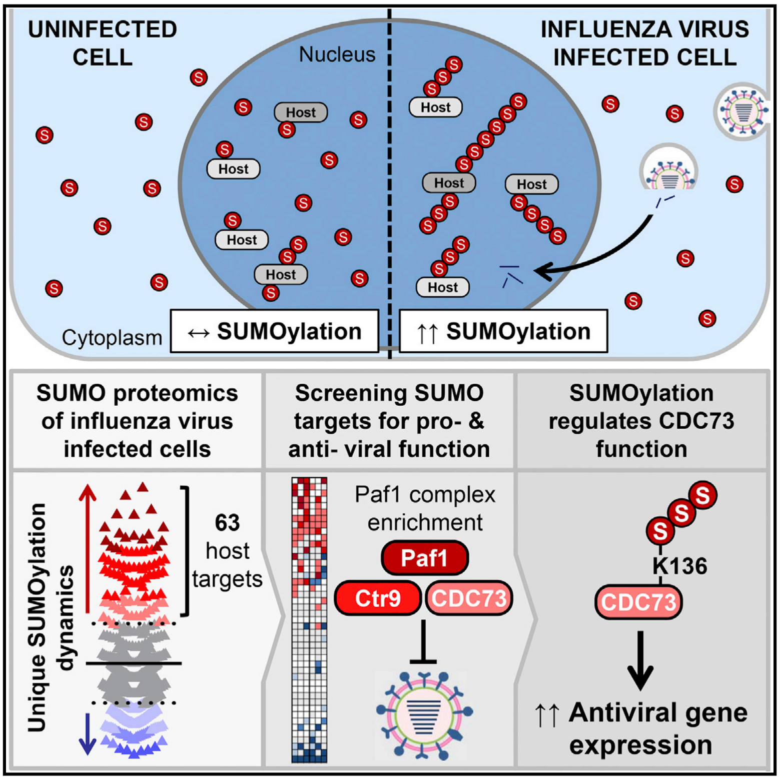

Highlights

- Nuclear-replicating RNA viruses trigger a host SUMOylation response
- Influenza virus RNA polymerase activity contributes to SUMO remodeling
- Influenza virus infection re-targets SUMO to a unique set of 63 host proteins
- SUMOylation of parafibromin (CDC73) potentiates antiviral gene expression

Authors

Patricia Domingues, Filip Golebiowski, Michael H. Tatham, Antonio M. Lopes, Aislynn Taggart, Ronald T. Hay, Benjamin G. Hale

Correspondence

hale.ben@virology.uzh.ch

In Brief

Dynamic SUMOylation is essential for cellular stress responses. Here, Domingues et al. describe a host SUMOylation response to nuclear-replicating influenza viruses. Using quantitative proteomics and functional screening, they reveal that infection uniquely re-targets SUMO to a diverse set of host proteins involved in transcription, RNA processing, and DNA damage repair.

Domingues et al., 2015, Cell Reports *13*, 1467–1480
November 17, 2015 ©2015 The Authors
http://dx.doi.org/10.1016/j.celrep.2015.10.001

Cell Reports
Resource

OPEN ACCESS
CellPress

Global Reprogramming of Host SUMOylation during Influenza Virus Infection

Patricia Domingues,[1,3,4] Filip Golebiowski,[1,3,4] Michael H. Tatham,[2] Antonio M. Lopes,[1,4] Aislynn Taggart,[1] Ronald T. Hay,[2] and Benjamin G. Hale[1,4,*]
[1]MRC-University of Glasgow Centre for Virus Research, Garscube Campus, 464 Bearsden Road, Glasgow G61 1QH, UK
[2]Centre for Gene Regulation and Expression, College of Life Sciences, University of Dundee, Dundee DD1 5EH, UK
[3]Co-first author
[4]Present address: Institute of Medical Virology, University of Zurich, Winterthurerstrasse 190, 8057 Zurich, Switzerland
*Correspondence: hale.ben@virology.uzh.ch
http://dx.doi.org/10.1016/j.celrep.2015.10.001
This is an open access article under the CC BY license (http://creativecommons.org/licenses/by/4.0/).

SUMMARY

Dynamic nuclear SUMO modifications play essential roles in orchestrating cellular responses to proteotoxic stress, DNA damage, and DNA virus infection. Here, we describe a non-canonical host SUMOylation response to the nuclear-replicating RNA pathogen, influenza virus, and identify viral RNA polymerase activity as a major contributor to SUMO proteome remodeling. Using quantitative proteomics to compare stress-induced SUMOylation responses, we reveal that influenza virus infection triggers unique re-targeting of SUMO to 63 host proteins involved in transcription, mRNA processing, RNA quality control, and DNA damage repair. This is paralleled by widespread host deSUMOylation. Depletion screening identified ten virus-induced SUMO targets as potential antiviral factors, including C18orf25 and the SMC5/6 and PAF1 complexes. Mechanistic studies further uncovered a role for SUMOylation of the PAF1 complex component, parafibromin (CDC73), in potentiating antiviral gene expression. Our global characterization of influenza virus-triggered SUMO redistribution provides a proteomic resource to understand host nuclear SUMOylation responses to infection.

INTRODUCTION

Reversible posttranslational modification of proteins provides cells with a rapid and dynamic mechanism to modulate proteome functionality in response to many stimuli, including pathogen invasion. Ubiquitin and ubiquitin-like modifiers (Ubls) have emerged as central players in mediating the host innate immune response to infection, and their diversity, coordination by specialized enzymatic cascades, and range of linkage topologies all contribute to an incredibly rich regulatory potential. Among Ubls, the small ubiquitin-like modifiers (SUMOs) are predominantly located in the cell nucleus, and they are reversibly attached to lysine residues in target proteins by only a small set of known enzymes (Hay, 2013). Nevertheless, SUMOs can conjugate to thousands of proteins (Hendriks et al., 2014; Tammsalu et al., 2014) and regulate distinct cellular processes, such as transcription, chromatin remodeling, DNA repair, and cell-cycle progression.

The SUMO conjugation machinery is highly responsive to stress stimuli, with global changes to SUMOylation occurring rapidly after cells have been exposed to heat shock (Golebiowski et al., 2009; Saitoh and Hinchey, 2000), proteasome inhibition (Lamoliatte et al., 2014; Tatham et al., 2011), or DNA damage (Hendriks et al., 2015; Yin et al., 2012). Thus, stress-triggered SUMOylation modulates nuclear functions as part of a resolution strategy to protect cell integrity. Recent studies also have implicated SUMOylation as playing a critical role in activating host intracellular pathogen defenses, particularly against DNA viruses that enter the nucleus (Boutell et al., 2011; Cuchet-Lourenço et al., 2011), but also against HIV-1 (Li et al., 2012) and bacteria (Fritah et al., 2014; Ribet et al., 2010). As such, many DNA viruses, as well as some bacteria, encode proteins that actively suppress host SUMOylation or reduce the global amount of SUMO conjugates in infected cells (Everett et al., 2013; Ribet et al., 2010). Notably, with clear parallels to the SUMOylation response triggered by environmental stresses, a nuclear-replicating HSV-1 mutant lacking the ability to degrade SUMO-modified proteins induces increased SUMO conjugate formation during infection and is restricted by an active SUMO system (Boutell et al., 2011). This suggests that cells sense DNA virus infection stress in the nucleus and (in the absence of a pathogen-encoded antagonist) respond by enhancing SUMOylation of certain targets to suppress replication.

Influenza viruses are atypical RNA viruses that replicate in host-cell nuclei and encode multiple proteins that become SUMOylated during infection in order to regulate their trafficking or function (Han et al., 2014; Santos et al., 2013; Wu et al., 2011; Xu et al., 2011). The inextricable linking of influenza viruses to nuclei is further exemplified by the coupling of viral RNA transcription to that of the host, the viral re-purposing of host-cell RNA splicing machinery, and the tethering of viral RNA genomes to cellular chromatin (Fodor, 2013). We speculated that the replication strategy of these nuclear RNA pathogens may induce a form of nuclear stress akin to that of an invading DNA virus,

thereby resulting in an analogous host SUMOylation response to resolve infection. Here we show that global remodeling of the host SUMO system is induced by nuclear-replicating influenza virus infections, but not other cytoplasmic-replicating RNA virus infections, and that influenza viral polymerase activity in the nucleus is a key contributor to this SUMO response. Furthermore, we utilize a system-wide quantitative mass spectrometry approach to identify the SUMO-modified proteome of human lung epithelial cells, and we quantify changes in host SUMO modification during influenza A virus (IAV) infection. Combining our proteomic results with a targeted gene-depletion screen and mechanistic studies, we uncover a host SUMOylation response to IAV infection that is distinct from that triggered by other cellular stresses, and we reveal several potential pro- and antiviral host factors whose function is regulated by SUMO. These data form a comprehensive proteomic and functional resource to understand the nuclear SUMO response to an RNA virus infection.

RESULTS

Nuclear-Replicating Influenza Viruses Induce Specific Remodeling of Host SUMO Conjugation and Localization

Human cells express three main SUMO paralogues as follows: SUMO2 and SUMO3, which only differ by 3 amino acids in their mature state (hereafter referred to as SUMO2/3); and SUMO1, which shares ~50% sequence identity with SUMO2/3. Consistent with the results of others (Pal et al., 2011), western blot analysis of total human lung epithelial cell (A549) lysates revealed that IAV infection triggers an increase in the abundance of proteins modified by both SUMO1 and SUMO2/3, whereas the amounts of free, unconjugated SUMO1 and SUMO2/3 are depleted (Figure 1A). This SUMOylation response is not due to an increase in SUMO mRNA transcripts during infection (Figure S1A), indicating that new SUMO conjugates arise from the pre-existing SUMO pool. Furthermore, this response is not unique to IAV, as influenza B virus (which also replicates in the nucleus) triggered similar SUMO conjugate induction during infection (Figure 1B). Nevertheless, infection with a panel of cytoplasmic-replicating RNA viruses (including members of the *Bunyaviridae* [−ve sense, segmented RNA genome], *Rhabdoviridae* [−ve sense, single-stranded RNA genome], and *Togaviridae* [+ve sense, single-stranded RNA genome]) revealed that these viruses do not trigger gross SUMO conjugate induction (Figure 1C). These data suggest a specific induction of SUMO conjugates in response to nuclear-replicating influenza viruses.

To further characterize SUMO remodeling during IAV infection, we studied the intracellular distribution of SUMO1 and SUMO2/3. SUMOs normally form discrete intra-nuclear foci (10–20 per cell), a fraction of which co-localizes with promyelocytic leukemia (PML) nuclear bodies (PML NBs) (Everett et al., 2013). In addition, a sub-population of SUMO1 localizes to the nuclear rim. We found that, concomitant with SUMO conjugate induction, IAV infection triggers dispersal of both SUMO1 and SUMO2/3 nuclear puncta (Figures 1D and S1B), a phenotype similar to that observed in response to DNA damage and heat shock stresses (Hendriks et al., 2015; Nefkens et al., 2003). Notably, components of PML NBs (such as hDaxx and SP100) also disperse during IAV infection, but PML itself only redistributes into smaller, yet more numerous, foci (Figure 1E). A strikingly similar specific redistribution of PML NB components has been observed previously in response to heat shock (Nefkens et al., 2003). However, western blotting did not reveal gross changes in PML SUMOylation following IAV infection (Figure S1C), suggesting that this SUMO remodeling is specific to certain cellular substrates. These data indicate that IAV-induced SUMO remodeling causes a redistribution of SUMO from sites including PML NBs to new targets that are distributed diffusely throughout the nucleus.

Influenza Virus RNA Polymerase Activity Contributes to Host SUMO Remodeling

IAV-triggered SUMOylation was not abrogated in cells functionally deficient in the cytoplasmic innate immune mediators MAVS, IRF3, and STAT1 (Figure S2A). Furthermore, there was no change in the kinetics of IAV-induced SUMOylation when small interfering RNAs (siRNAs) were used to deplete infected cells of NS1, the major IAV interferon antagonist (Figure S2B). In addition, an IAV infection-like SUMOylation response was not observed following type I/II interferon stimulation or following stimulation of canonical innate immune responses by a defective-interfering particle-rich stock of Sendai virus (Figure S2C). These data are consistent with previous observations disconnecting IAV-induced SUMOylation from interferon responses (Pal et al., 2011). We therefore hypothesized that a form of viral stress distinct from that triggering classical innate immune pathways may be responsible for IAV-induced SUMOylation. However, an IAV-like broad SUMO response could not be triggered using chemical stimuli promoting ER stress, DNA damage, or apoptosis, three canonical cellular stresses we suspected might occur during IAV infection, but that were not active at times when IAV-stimulated SUMOylation was apparent (Figures S2D and S2E).

We used small-molecule inhibitors and UV-inactivation methods to map IAV-triggered SUMOylation to a process requiring viral genome replication and protein synthesis, but not genome nuclear export or later stages of the virus replication cycle, such as virion budding (Figure 2A). Given the tight association of active influenza virus replication complexes with nuclear processes (a distinguishing feature from cytoplasmic-replicating RNA viruses), we speculated that the stress of nuclear IAV polymerase activity may contribute to host SUMO remodeling. To test this hypothesis, we used a transfection-based mini-replicon reporter system, whereby viral ribonucleoprotein complexes (vRNPs) consisting of viral NP, PB1, PB2, and PA are assembled together in the nucleus following plasmid expression along with a negative-sense viral-like RNA genomic segment encoding mCherry. As the viral-like RNA cannot be transcribed into mRNA by cellular polymerases, mCherry protein is only produced in cells expressing all five viral components. Furthermore, the mCherry construct is unspliced such that this assay recapitulates IAV RNA transcription and replication, but not splicing.

Using SUMO foci dispersion as a single-cell readout of host SUMO proteome remodeling, we found that cells expressing actively replicating IAV RNP complexes had significantly fewer SUMO1 and SUMO2/3 foci than cells expressing all the viral

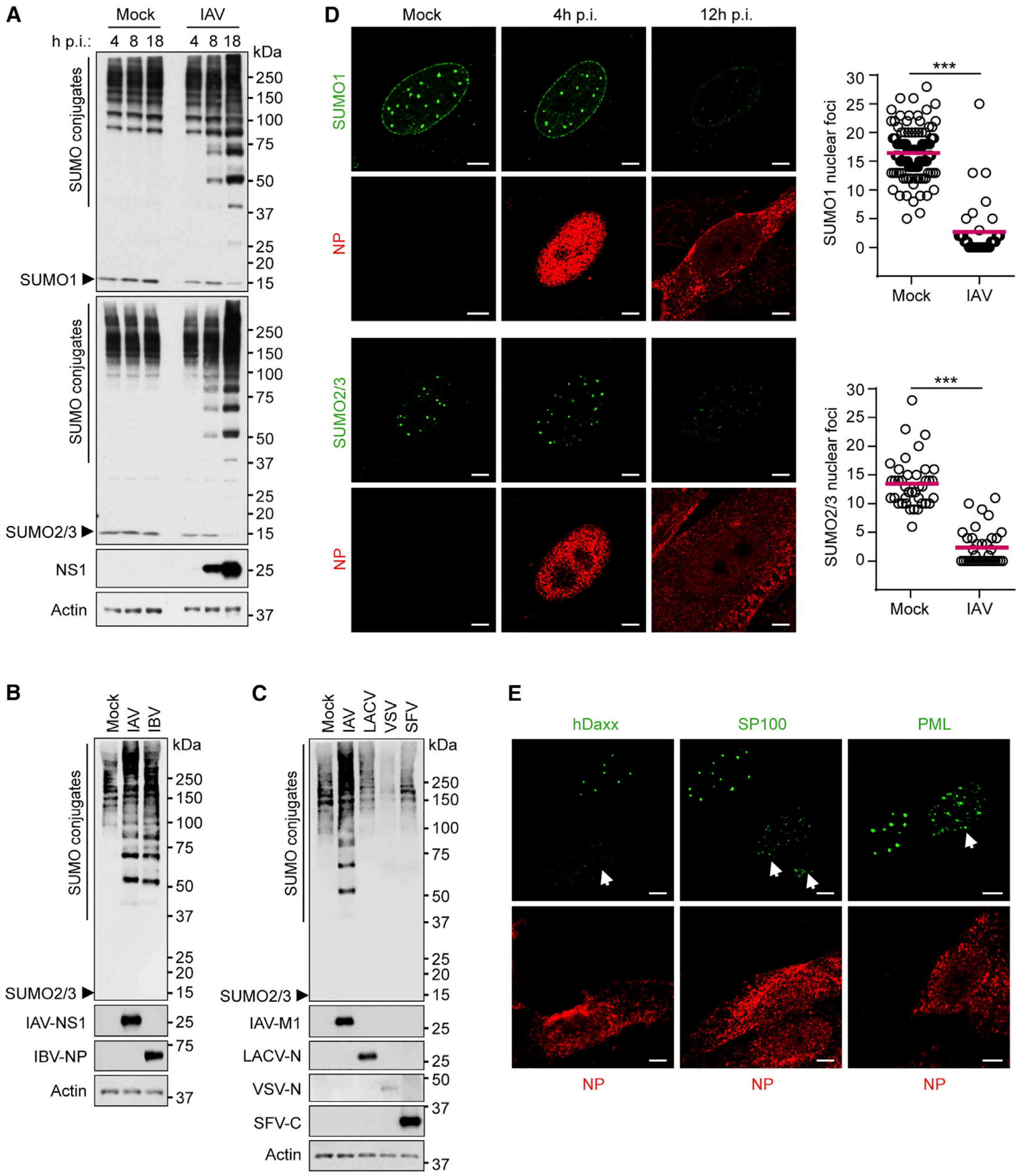

Figure 1. SUMO Conjugation Patterns and Intracellular Distribution following Infection with a Panel of Nuclear- and Cytoplasmic-Replicating RNA Viruses

(A) Western blot of lysates from A549s infected with IAV (5 PFU/cell) as indicated. SUMO1, SUMO2/3, NS1, and actin were detected.

(B and C) Western blot of Vero cells infected with IAV or influenza B virus (IBV) for 16 hr at 33°C (B) or infected with IAV, LACV, VSV, or SFV for 12 hr at 37°C (all ~5 PFU/cell) (C). SUMO2/3, actin, and individual viral proteins were detected.

(D and E) Immunofluorescent analysis and quantification of MRC5s infected with IAV at 0.1 PFU/cell as indicated. SUMO1 and SUMO2/3 (D) or hDaxx, SP100, PML (E), and IAV NP were visualized after staining. Scale bars represent 5 μm. Statistical significance (***$p < 0.0001$) in (D) was determined using the Student's t test. See also Figure S1.

protein components of the RNP in the absence of viral-like RNA or mock-transfected cells (Figures 2B and 2C). In addition, expression of each individual vRNP component, or negative-sense viral RNA together with NP, failed to stimulate SUMO remodeling (Figures 2D and 2E). To assess directly the contribution of viral polymerase activity (rather than basic processes such as RNP formation) on SUMO redistribution, we also tested inactive RNPs in this assay, either by omitting an essential polymerase

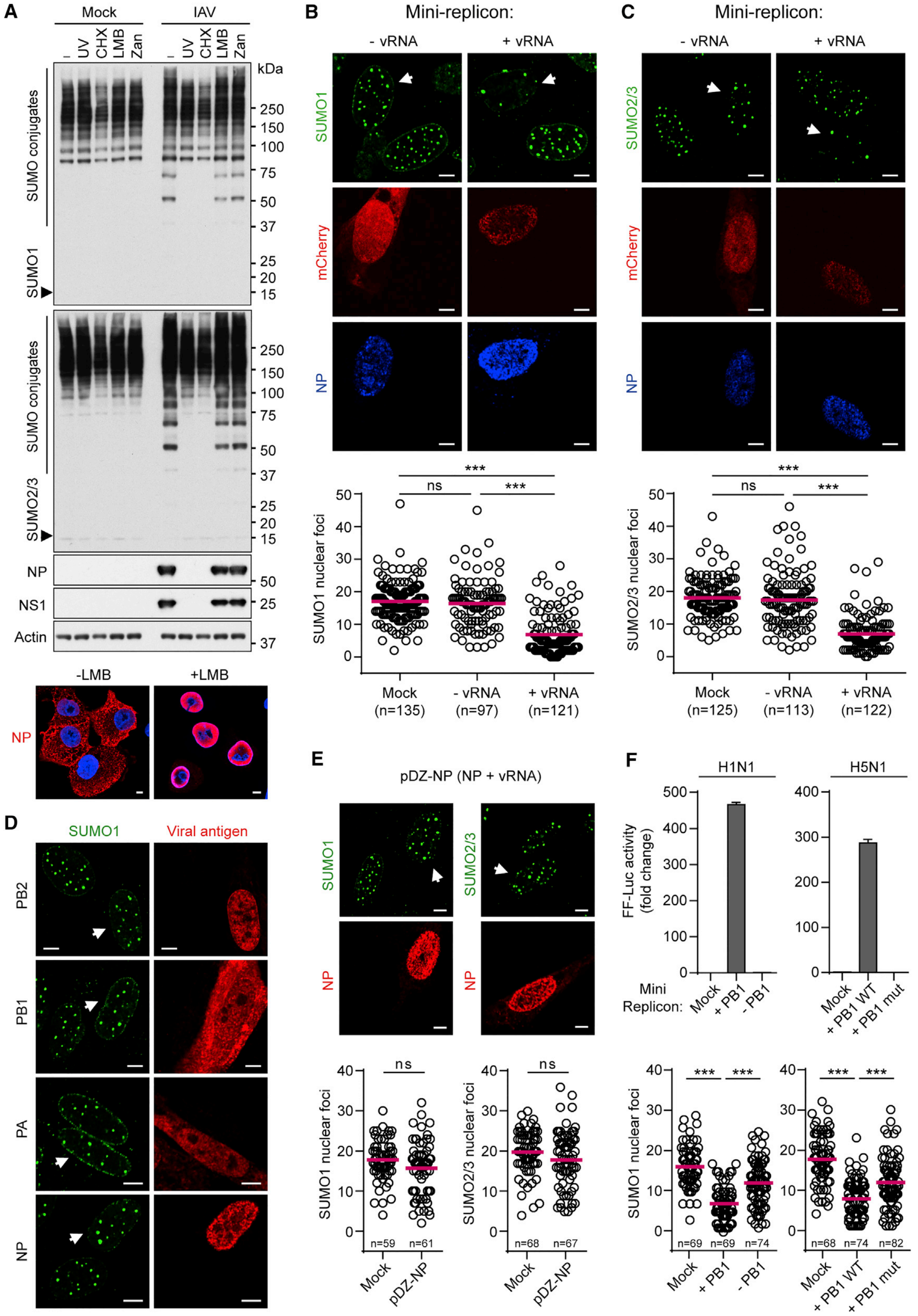

(legend on next page)

component or by substituting in a mutant polymerase. In both cases, polymerase activity was completely abrogated, as determined by standard mini-replicon assays, and concomitantly there was a significant relief in RNP-triggered SUMO redistribution (Figure 2F). Overall, these data suggest that IAV RNA polymerase activity in the nucleus (transcription processes or replication enhancing vRNA/cRNA levels, but probably not splicing) is a stimulus for triggering remodeling of the host SUMO system during infection, and they are consistent with the hypothesis that virus-induced SUMOylation is specific to nuclear-replicating viruses, rather than RNA viruses in general. We propose that IAV polymerase activity in the nucleus triggers a previously unappreciated form of nuclear stress that is regulated by SUMOylation.

SILAC-Based Quantitative Proteomics of IAV-Induced SUMO Remodeling

To survey the dynamics of cellular and viral protein SUMOylation during IAV infection, we adopted a quantitative proteomic strategy that has been used previously to identify changes in SUMO modification in response to proteotoxic stresses (Golebiowski et al., 2009; Tatham et al., 2011). We generated A549 cell lines stably expressing either SUMO1 or SUMO2 fused to an N-terminal tandem affinity purification (TAP) tag. An additional A549 cell line stably expressing the TAP tag only (TAP only) was generated as a negative control (Figures S3A and S3B). Both heterologously expressed TAP-SUMO1 and TAP-SUMO2 conjugated to endogenous cellular proteins under normal growth conditions in the respective cell lines, and conjugation of these tagged SUMO forms was robustly enhanced following IAV infection (Figure S3C), indicating that the tag did not interfere with SUMO conjugation and that these constructs faithfully recapitulate endogenous SUMOylation changes in response to infection.

We conducted two independent stable isotope labeling by amino acids in cell culture (SILAC) experiments to determine the impact of IAV infection for 10 hr on the SUMO1 and SUMO2 sub-proteomes of A549 cells (Figure 3A). This time point was chosen to ensure all infected cells had undergone a full single cycle of virus replication and to capture primary dynamic SUMOylation changes. Notably, <2% of cellular proteins varied in total abundance more than ~2-fold either between the TAP-only and TAP-SUMO cell lines or after IAV infection (Figures S3D and S3E). In contrast, analysis of the purified samples showed that ~32% (SUMO1) and ~47% (SUMO2) of quantified proteins were >2-fold more abundant in the purified TAP-SUMO material compared with the purified TAP-only material, and ~36% (SUMO1) and ~25% (SUMO2) of quantified proteins varied >2-fold in abundance in the purified TAP-SUMO material following IAV infection (Figures S3F and S3G). Together, this suggests that a large proportion of identified and quantified proteins in the purified, but not crude, samples show specific changes in abundance relating to SUMO modification status, as well as a dependence upon infection for SUMO conjugation state.

Using a false discovery threshold of 1%, we identified and quantified 587 putative SUMO1 substrates and 815 putative SUMO2 substrates in A549 cells (Figure 3B; Tables S1 and S2). Bioinformatic comparison of the combined 895 putative SUMO substrates with those identified in independent studies using different cell types revealed that 89% of our assigned SUMO substrates have been described as SUMO targets previously (Figure S3H; Table S3). We identified 506 putative substrates as common to both SUMO1 and SUMO2 (Figure S3I), and, consistent with the roles of SUMO, gene ontology analysis using the Enrichr platform (Chen et al., 2013) revealed enrichment of these substrates for cellular compartments including the nucleolus, nucleoplasm, chromatin, and PML NBs as well as molecular function enrichment for chromatin binding, transcription coactivator/corepressor activity, histone binding, and transcription factor binding (Table S3). Furthermore, >76% of these common SUMO substrates recently have been confirmed as bona fide SUMO substrates by high-resolution mass spectrometry-based SUMO modification site-mapping techniques, which identify SUMO-modified lysine residues (Table S4). Such a high degree of overlap with other studies, combined with the gene ontology analysis, supports the validity of our approach in identifying SUMO substrates in human lung A549 cells.

Triple SILAC maps (tsMAPs) of the putative host SUMO substrates illustrated that, surprisingly, the bulk of substrates exhibited reduced SUMO modification (357 for SUMO1 and 245 for SUMO2) or unchanged SUMOylation following IAV infection (Figure 3B; Tables S1 and S2). Strikingly, only 76 SUMO1 substrates (13%) and 117 SUMO2 substrates (14%) increased substantially in SUMO modification status (up to ~35-fold) during IAV infection (Figure 3B; Tables S1 and S2). Thus, although the

Figure 2. IAV Polymerase Activity Contributes to SUMO Remodeling

(A) Western blot of lysates from IAV-infected A549 cells treated with different inhibitors. Cells were infected with IAV or UV-inactivated IAV (UV) at 5 PFU/cell, followed by incubation with 50 μg/ml cycloheximide (CHX), 11 nM leptomycin B (LMB), or 10 μM zanamivir (Zan) for 12 hr. SUMO1, SUMO2/3, NS1, NP, and actin were detected. (Bottom) Immunofluorescence shows NP staining at 12 hr post-infection in A549s ± LMB. DAPI was used to stain DNA.

(B and C) Immunofluorescent analyses of MRC5s transiently expressing PB1, PB2, PA, NP, and a negative-sense viral-like mini-replicon mCherry reporter construct (+vRNA), or PB1, PB2, PA, NP, and mCherry (no viral-like reporter; −vRNA).

(D) Immunofluorescent analysis of MRC5s individually expressing PB1, PB2, PA, or NP.

(E) Immunofluorescent analysis of MRC5s transiently transfected with pDZ-NP, which expresses both NP protein from a pol-II promoter and NP vRNA from a pol-I promoter.

(F) (Top) Luciferase-based mini-replicon assays in 293Ts to assess polymerase activity. (Left) (WSN, H1N1) Cells transiently expressing PB1 (or not), PB2, PA, NP, and a negative-sense viral-like mini-replicon Firefly luciferase reporter construct. (Right) (KAN-1, H5N1) Cells transiently expressing PB1 (or an E445A/E446A inactive mutant), AvianPr-PB2-E627K, PA, NP, and a negative-sense viral-like mini-replicon Firefly luciferase reporter construct. Bars represent mean values from triplicates (±SD). (Bottom) Quantification of SUMO1 nuclear foci for the conditions indicated above as determined by the mCherry-based mini-replicon reporter assay in MRC5s. For (B)–(F), cells were transfected for 36 hr prior to processing or fixation and immunostaining. Representative images are shown. Scale bars represent 5 μm. Statistical significance in panels (B), (C), (E), and (F) was determined using the Student's t test (***$p < 0.0001$; ns, non-significant). See also Figure S2.

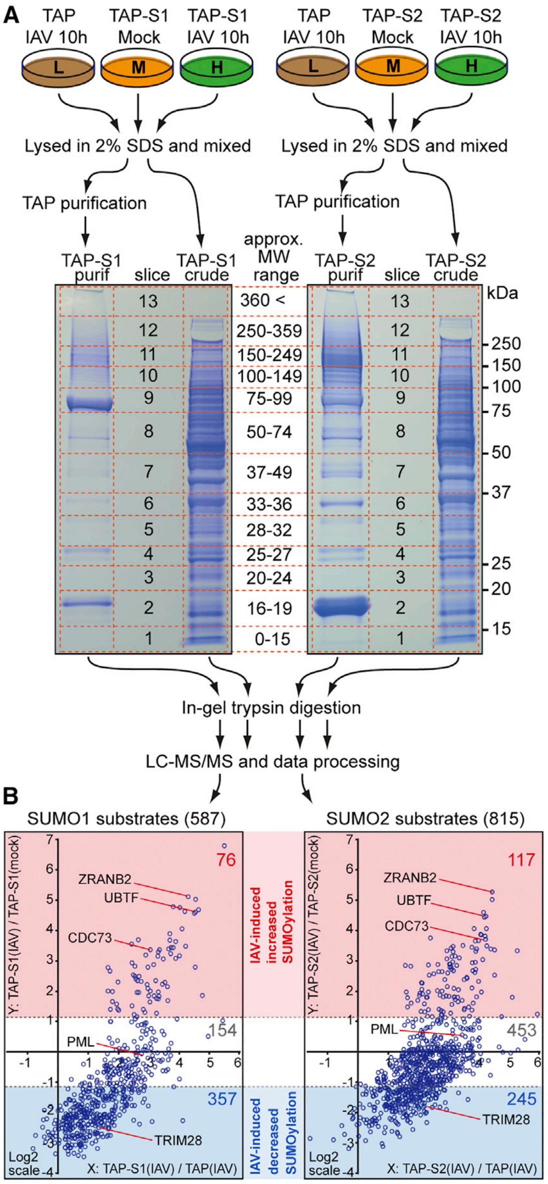

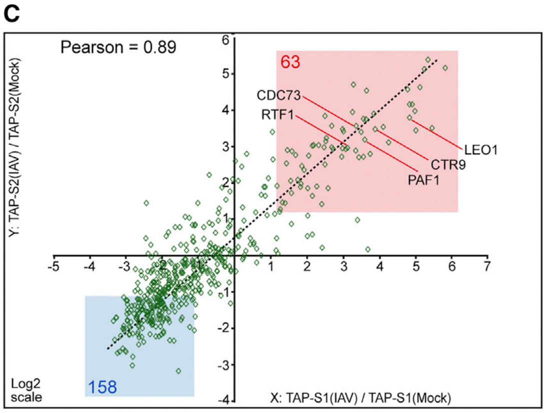

D **IAV-induced SUMO targets (63)**

Functional category	Gene name
Cell cycle	ANP32B, CDK11B
Chromatin remodeling	BRD2, CFDP1, CHD1, CHD2, CHD8, RAD54L2, SRCAP
Chromatin remodeling (FACT complex)	SSRP1, SUPT16H
DNA damage repair	BLM, EME1, PIAS4, RAD18, RNF111
DNA damage repair (SMC5/6 complex)	NDNL2, NSMCE1, NSMCE2, NSMCE4A, SMC5, SMC6
Transcription elongation	AFF4, EAF1, HTATSF1, IWS1, MLLT3, SUPT5H
Transcription elongation (PAF1 complex)	CDC73, CTR9, LEO1, PAF1, RTF1
Transcription initiation	TAF1, TAF3
Transcription regulation	BAZ2B, BCL9L, DAXX, HIPK2, PHF2, SAP30BP, SETX, SPEN, UBTF
mRNA processing	CPSF1, CPSF2, FIP1L1, RBBP6, WDR33
mRNA processing (splicing)	CLASRP, SFPQ, ZRANB2
RNA quality control	PAPD5, ZC3H18, ZCCHC7
Miscellaneous	C18orf25, GPATCH8, NKTR, PIK3C2A, PPP1R26, SUMO2, ZFR, ZNF609

Figure 3. Quantitative SUMO Proteomics of IAV-Infected Cells

(A) SILAC-based SUMO1 and SUMO2 proteomic workflow. The specified A549s were grown for five to six cell doublings in light (L; isotopically normal, K0R0), medium (M; K4R6), or heavy (H; K8R10) SILAC medium prior to treatment and processing as indicated.

(B) The tsMAPs of SUMO1 (left) and SUMO2 (right) substrates after data filtering, indicating log2-fold changes in protein modification following IAV infection (y axis). The numbers of substrates identified in each category are indicated and certain examples are highlighted.

(C) Correlation of log2-fold changes in SUMO1 and SUMO2 substrate modification following IAV infection. The 63 substrates that increase (and 158 substrates that decrease) in both SUMO1 and SUMO2 modification following IAV infection are highlighted, and certain example proteins are labeled.

(D) The 63 host substrates that increase in SUMOylation with IAV infection organized by manually curated functional category. See also Figure S3 and Tables S1, S2, S3, S4, and S7.

original western blotting experiments suggested an overall increase in SUMOylation upon infection, it is clear that IAV-induced SUMOylation of substantial numbers of substrates occurs concomitantly with the deSUMOylation of a different set of proteins. Notably, protein deSUMOylation occurred to a much lower extent (maximum ~8-fold decrease) than SUMOylation, suggesting widespread dynamic exchange of SUMO during infection from the bulk of pre-existing substrates to a restricted set of new cellular targets.

Comparison of the quantitative changes to SUMO1 and SUMO2 conjugation in response to IAV infection demonstrated a high degree of correlation (Pearson's coefficient of 0.89) (Figure 3C), indicating no gross differences between SUMO1 and SUMO2 paralogues, although some paralogue-specific SUMO

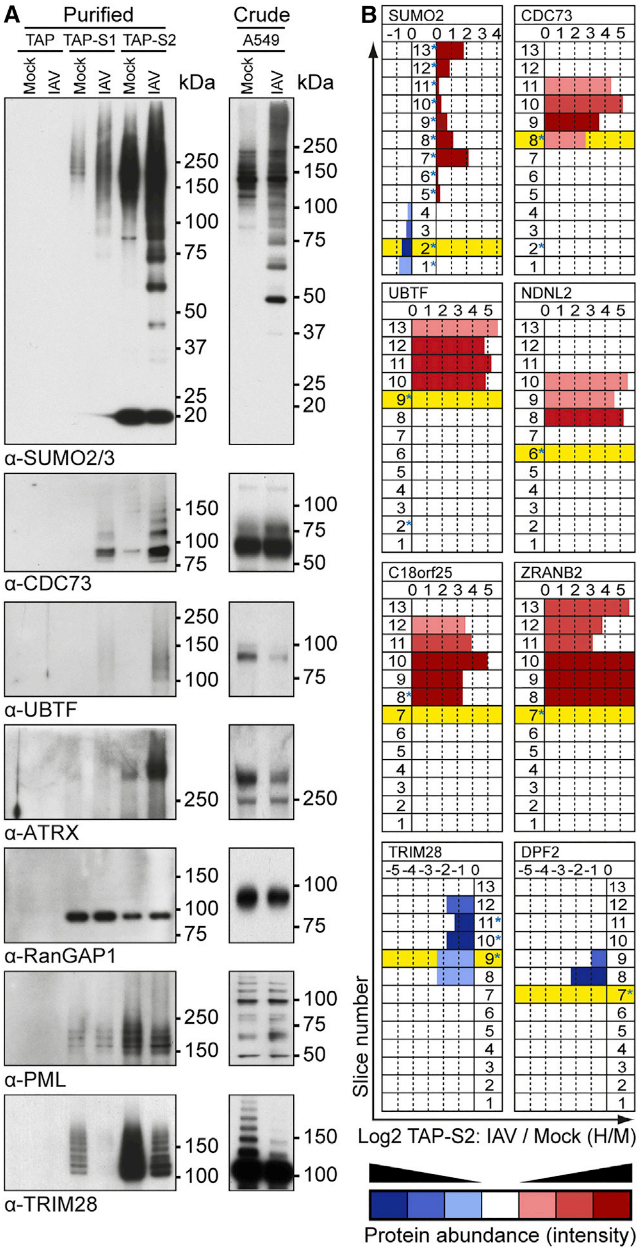

Figure 4. Western Blot and Bioinformatic Validation of IAV-Induced SUMO Targets

(A) TAP-purified (left) or crude lysate (right) samples from specified A549s either mock or IAV infected were subjected to western blot analysis for the indicated substrates.

(B) Predicted molecular weight (preMW) of substrates (unmodified) was compared to their observed electrophoretic mobility (obsEM) across all 13 slices (crude and TAP-SUMO2 purified, IAV/Mock). Yellow background indicates slice where unmodified protein would be expected based on its preMW. Bar length indicates change in protein ratio between IAV- and mock-infected (H/M) purified TAP-SUMO2 samples. Intensity of color indicates protein abundance (intensity) in each slice; red was nominally given to increased H/M ratios while blue was nominally given to decreased H/M ratios. Asterisks indicate obsEM of specified proteins in crude lysate. See also Figures S4 and S5.

modification changes were observed (Table S3). Thus, the combined SUMO1 and SUMO2 data represent a common and stringent consensus of SUMO substrate changes following IAV infection. Using such criteria, we define 63 host proteins as increasing in SUMO modification with IAV infection and 158 proteins as decreasing in SUMO modification (Figures 3C and 3D; Table S3).

Validation of IAV-Induced Host SUMOylation Remodeling

We took both a biochemical and a bioinformatic approach to validate the SILAC ratios obtained for IAV-induced SUMOylation changes. Given that only a small proportion of any given substrate population is usually SUMO modified, detection of conjugated forms of proteins is notoriously difficult without prior enrichment. We therefore used immunoblotting for endogenous cellular proteins to analyze SUMO1 and SUMO2 immunoprecipitation samples from IAV-infected A549s or TAP purification samples from independent IAV infections of the TAP-only, TAP-SUMO1, and TAP-SUMO2 cell lines. As shown in Figures 4A and S4, we could validate the IAV-enhanced conjugation of SUMO1 and SUMO2 to endogenous cellular proteins, such as CDC73, UBTF, and ATRX. Interestingly, we also confirmed IAV-enhanced conjugation of SUMO2 in the TAP-SUMO1 samples, suggestive of a possible increase in SUMO1-capped SUMO2 chains or the increased conjugation of both SUMO1 and SUMO2 to the same target proteins. We also confirmed our mass spectrometry data that RanGAP1 and PML are basally SUMOylated but largely do not change in modification status following IAV infection, whereas TRIM28 is highly deSUMOylated during infection. Importantly, independent western blot analysis of total A549 cell lysates following infection revealed that the abundance of the major unconjugated forms of all these target proteins does not increase in response to IAV infection, an observation consistent with the mass spectrometry quantification of each protein in crude lysates and indicative of a specific effect of infection on SUMO modification status (Figures 4A and S4; Tables S1 and S2).

To further validate the IAV-triggered host SUMOylation changes for additional targets without suitable antibodies available, we developed a slice-by-slice bioinformatic analysis based on scrutinizing the change in electrophoretic mobility of target proteins in TAP-SUMO2-purified mass spectrometry samples in response to IAV infection. First, using mass spectrometry data obtained from individual gel slices, we compared the migration pattern of selected putative targets in total crude lysates with their migration pattern in TAP-SUMO2-purified samples, defining each target as SUMO2 modified if it migrated slower in the purified sample than expected based on its predicted molecular weight. In addition, for each target and gel slice, we analyzed the change in peptide ratio upon IAV infection in the TAP-SUMO2-purified samples in order to determine the magnitude of SUMOylation change at a given mobility.

As shown in Figure 4B, such an analysis of SUMO2 revealed that during IAV infection SUMO2 shifted from a faster migrating species (its unconjugated form) to several slower migrating species distributed throughout multiple gel slices, indicating that increased SUMO2 conjugation during IAV infection is partly a

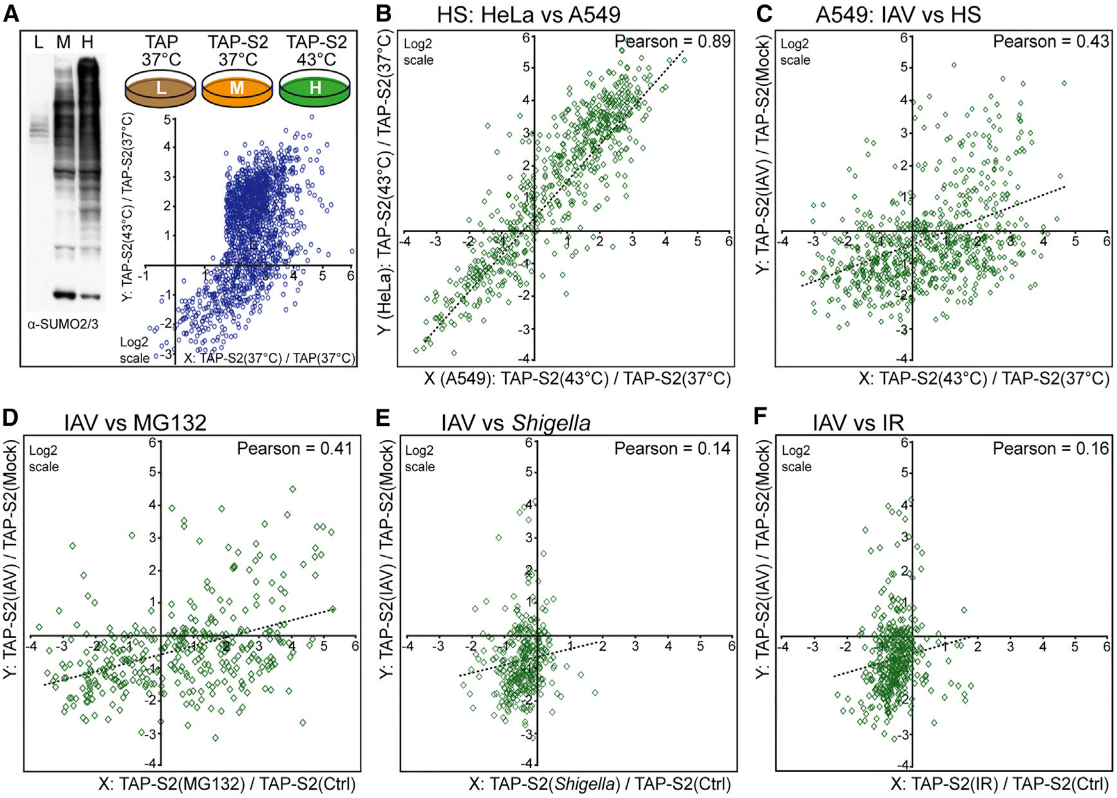

Figure 5. The IAV-Induced Host SUMOylation Response Is Quantitatively Distinct from that Induced by Other Stresses

(A) Quantitative SUMO2 proteomics of the heat shock response in A549s. The indicated A549s were grown for five to six cell doublings in light (L; isotopically normal, K0R0), medium (M; K4R6), or heavy (H; K8R10) SILAC medium prior to treatment (for 30 min) as indicated. Subsequent TAP purification and analyses were performed as for Figure 3. Graph shows tsMAP of SUMO2 substrates after data filtering, indicating log2-fold changes in protein modification following heat shock (y axis).

(B–F) Correlations show log2-fold changes in SUMO2 modification in response to heat shock between HeLa and A549 cells (B); between IAV infection and heat shock in A549 cells (C); between IAV infection in A549 cells and proteasome inhibition (MG132) in HeLa cells (D); between IAV infection in A549 cells and *Shigella flexneri* infection in HeLa cells (E); and between IAV infection in A549 cells and ionizing radiation (IR; 15 Gy) in HeLa cells (F). See also Figure S5 and Table S5.

consequence of depleting unmodified SUMO2. Strikingly, similar analyses revealed that host protein examples, such as CDC73, UBTF, NDNL2, C18orf25, and ZRANB2, all migrated slower than expected in the TAP-SUMO2-purified samples (indicative of their SUMO2 modification), and their abundance in these slower migrating forms was highly enhanced upon IAV infection (indicative of increased SUMOylation). In contrast, host proteins such as TRIM28 and DPF2 also migrated slower than expected in TAP-SUMO-purified samples, although their abundance in these fractions decreased with infection, suggesting a decrease in their SUMOylation that correlated with the mass spectrometry and western blot analyses (Figure 4B). These validation examples further strengthened the confidence in our mass spectrometry dataset as a whole.

IAV Proteins as SUMO Targets

Several IAV proteins have been described as targets for SUMO modification (Pal et al., 2011; Santos et al., 2013; Wu et al., 2011; Xu et al., 2011). Analysis of our TAP-SUMO-enriched mass spectrometry data using a stringent 1% false discovery threshold and our slice-by-slice bioinformatic strategy revealed that only NS1, M1, and NEP satisfied our filtering criteria for SUMO1- and SUMO2-modified targets during infection (Figures S3F, S3G, and S5A). NS1 and M1 have been studied extensively as SUMO targets, but NEP has not previously been identified as a bona fide SUMO substrate during IAV infection. These mass spectrometry data therefore support the rationale for future studies investigating functional consequences of viral protein SUMOylation.

IAV-Induced Host SUMOylation Responses Are Distinct from Those Triggered by Canonical Stress Stimuli

A multitude of stresses has been demonstrated to modulate cellular SUMO modification dynamics, including heat shock, ionizing radiation, proteotoxicity, and bacterial infection. Strikingly, heat shock causes a global increase in total SUMO conjugates that, by western blot, appears similar to that observed with IAV infection (Figure 5A). To compare the cellular SUMOylation response to IAV with the response to heat shock treatment, we also used SILAC proteomics to determine how the SUMO2-modified proteome changes in TAP-SUMO2 A549 cells following incubation at 43°C for 30 min (Figure 5A; Table

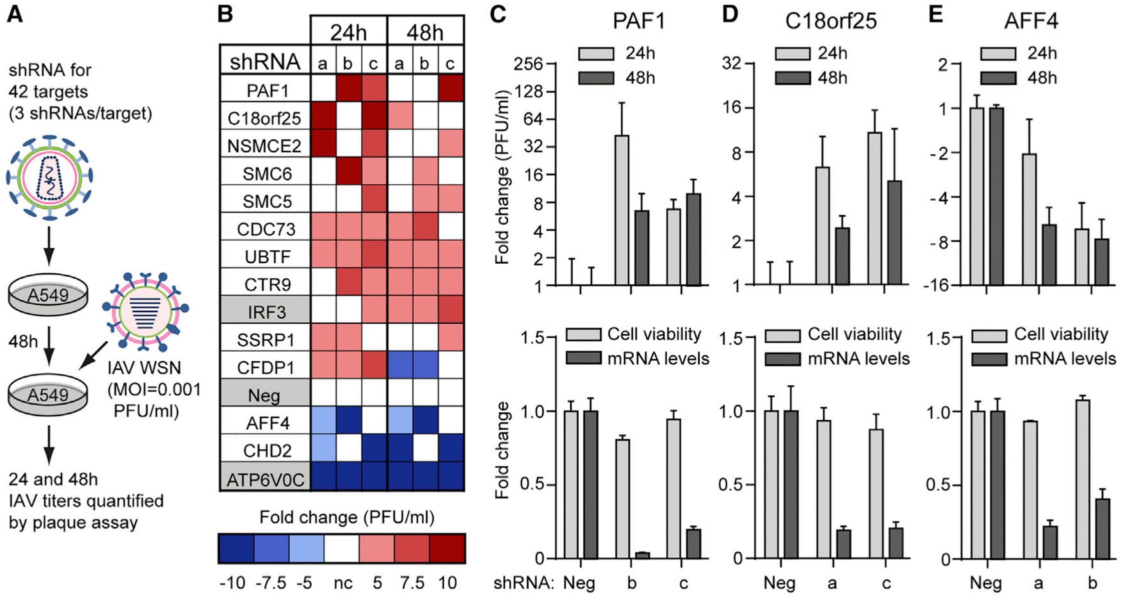

Figure 6. SUMO Targets Impacting IAV Replication

(A) Schematic representation of the lentivirus-based shRNA screen assessing 42 host SUMO targets for their impact on IAV replication.

(B) Heatmap summary of factors identified as required or restrictive to IAV replication in A549 cells. Genes are shown whose depletion led to a 5-fold or more difference in infectious IAV titer as compared with control for at least two of three shRNA sequences. Each individual shRNA is labeled a, b, or c and control shRNAs are highlighted in gray.

(C–E) Validation of PAF1 (C), C18orf25 (D), and AFF4 (E) as impacting IAV replication. The two shRNA sequences for each gene from (B) that showed consistent impact on IAV replication were independently validated in the same assay for their effect on IAV replication (top) and specific gene knockdown and effect on cell viability (bottom). Bars represent mean values from triplicates (±SD). See also Table S6.

S5). Heat-shock-triggered SUMO2 conjugation changes in A549s strongly correlated with those previously determined in HeLa cells using similar methodologies (Golebiowski et al., 2009), thereby confirming the validity of our data and indicating that this cellular stress induces a cell-type-independent SUMO response (Pearson's coefficient of 0.89, Figure 5B). Nevertheless, the IAV-triggered SUMOylation response in A549s did not quantitatively correlate with the heat shock SUMOylation response (Pearson's coefficient of 0.43) (Figure 5C).

Furthermore, given that our analysis of A549 and HeLa heat shock data indicated a common SUMO response in both A549 and HeLa cells, we took advantage of several HeLa SUMO proteomic datasets generated under various stress conditions (Fritah et al., 2014; Tatham et al., 2011; Yin et al., 2012) and compared them with our IAV-induced SUMO response in A549s. Notably, we also did not find a correlation between the IAV-induced SUMO response and the SUMO responses to proteasome inhibition (MG132 treatment), infection with *Shigella flexneri*, or ionizing radiation (Figures 5D–5F). On a qualitative level, bioinformatic analysis revealed distinct pathways that were enriched in enhanced SUMOylation following individual stresses (Table S7). For example, IAV-induced SUMOylation targets were enriched in members of the human PAF1 complex (PAF1C) and several categories relating to RNA polymerase II function, while *Shigella flexneri*-induced targets were enriched in centromer chromatin complex members. Heat shock stress caused SUMO to redistribute to a wider range of targets, with the most enriched categories including the spliceosome, the polycomb repressive complex, and the DNA synthesome complex (Table S7). These comparative observations suggest that IAV infection triggers a host SUMOylation response that is phenotypically distinct from responses to canonical cellular stresses, highlighting a potentially unique stress to the cell induced by viral RNA polymerase activity in the nucleus.

shRNA Screening Identifies IAV-Triggered SUMO Targets as Pro- and Antiviral Host Factors

Consistent between our SUMO1 and SUMO2 proteomic studies, IAV infection triggered a >4-fold increase in SUMOylation of 42 host proteins. To identify functional roles for these core SUMO targets during IAV infection, we depleted A549 cells of the corresponding 42 genes one by one using small hairpin RNA (shRNA)-expressing lentiviruses (three per gene), and we determined the subsequent replication of IAV by measuring infectious virus yields at 24 and 48 hr post-infection (Figure 6A). As controls, we also assessed the impact on IAV replication of depleting IRF3, a host antiviral defense transcription factor, and ATP6V0C, a vacuolar ATPase component required for efficient IAV entry (König et al., 2010). We classified a host gene as important for IAV replication if at least two of three shRNAs increased or decreased infectious IAV yields by at least 5-fold at a minimum of one time point compared to the non-targeting shRNA.

Using these criteria, we identified ten putative host antiviral factors among the IAV-induced SUMO targets and two required factors (Figure 6B; Table S6). For several of these factors, independent experiments confirmed that the shRNAs efficiently

depleted the target host mRNA, had minimal effect on cell viability, and reproducibly impacted IAV replication (Figures 6C–6E; Table S6). Notably, our shRNA screen identified three members of the human SMC5/6 complex (SMC5, SMC6, and the SUMO E3 ligase NSMCE2) as potential IAV antiviral factors, along with three members of PAF1C (PAF1, CTR9, and CDC73) and two PAF1C-associated proteins (SSRP1 and CFDP1). These data indicate that the IAV-triggered SUMOylation response targets both pro- and antiviral host factors, and they suggest a thus far unknown role for the human SMC5/6 DNA damage repair complex in IAV restriction.

SUMOylation of CDC73 Promotes Antiviral Gene Expression

In our proteomic screen, all components of PAF1C increased highly in SUMOylation during IAV infection (Figure S5B), and PAF1C was the most highly enriched functional group among all the substrates with infection-enhanced SUMOylation (Table S7). An antiviral role for the PAF1 component of PAF1C during IAV infection has been previously attributed to its potentiation of interferon-stimulated gene (ISG) expression (Marazzi et al., 2012). However, the contribution of SUMO modification to this process is unknown. To explore mechanistically how IAV-triggered SUMOylation may impact the function of PAF1C, we focused follow-up studies on CDC73 (also known as parafibromin or HRPT2), a core component of PAF1C with SUMO modification sites recently mapped by mass spectrometry (Hendriks et al., 2014; Lamoliatte et al., 2014; Tammsalu et al., 2014), and which our shRNA screening data revealed as a potential host antiviral factor.

In agreement with the antiviral role of PAF1 in mediating RNA polymerase II transcription elongation of ISGs (Marazzi et al., 2012), we found that siRNA-mediated depletion of endogenous CDC73 resulted in defective induction of ISG15 mRNA following IFNα treatment (Figure 7A). In addition, overexpression of CDC73 alone was able to stimulate expression from a promoter containing an interferon-stimulated response element (ISRE) in a dose-dependent manner (Figure 7B). The effect of CDC73 overexpression was not limited to ISRE-containing promoters, as a similar enhancing effect was observed for an NF-κB promoter, although minimally for the IFNβ promoter reporter, indicating a degree of specificity in CDC73's capacity to regulate inducible gene expression (Figure 7C). Promoter stimulation in these assays was specific to CDC73 overexpression, as co-transfection of siRNAs targeting CDC73 mRNA ablated protein production downstream of the ISRE promoter (Figure 7D). Furthermore, consistent with a model for CDC73 acting in RNA polymerase II-mediated transcription elongation, we found that the effect of CDC73 on ISRE promoter-driven expression was insensitive to depletion of the STAT1 transcription factor, which is otherwise essential for IFNα-stimulated activation of the ISRE (Figure 7E). These data suggest that CDC73 may act as an antiviral factor by potentiating inducible antiviral gene expression at a level subsequent to transcription factor activation.

To evaluate the role of CDC73 SUMO modification in transcription of inducible genes, such as ISGs, we assessed the ability of CDC73 to stimulate the ISRE reporter in the context of co-expressed human SENP2, a deSUMOylating enzyme previously implicated in regulating host antiviral responses (Ran et al., 2011). We found that SENP2 was able to antagonize CDC73-mediated activation of the ISRE reporter, suggesting that SUMOylation of CDC73 is important for this function (Figure 7F). In addition, we screened the ISRE-stimulating capabilities of a panel of three CDC73 mutants with lysine-to-arginine substitutions at sites positively identified by mass spectrometry to be SUMO modified using high-confidence remnant immunoaffinity profiling methods (Lamoliatte et al., 2014; Tammsalu et al., 2014). Notably, the single K136R substitution abrogated CDC73-mediated ISRE-dependent expression, while arginine substitutions at lysines 301 and 385 did not abrogate this response (Figure 7G).

Recent studies have validated CDC73-K136 as a bona fide SUMO modification site in vitro using purified recombinant proteins (Lamoliatte et al., 2014). We found that K136 is a major site for CDC73 SUMOylation in transfected cells, with the K136R substitution alone leading to highly reduced levels of SUMO-modified CDC73 (Figure 7H). K136 is located within an NLS of CDC73, and the arginine substitution at this site also leads to a subtle shift in the nuclear-cytoplasmic distribution of CDC73 (Figure S6), which may be indicative of SUMO modification contributing to the nuclear retention of CDC73. We note that CDC73 SUMOylation can be enhanced by other cellular stresses, including heat shock (Figure S5C), and interestingly the CDC73-K136R SUMOylation mutant has been previously shown to be defective in localizing to PML NBs in response to proteotoxic stress (Lamoliatte et al., 2014). Based on these data, we propose a general function for stress-triggered CDC73 SUMOylation in regulating stress-inducible genes that are required for resolution of cell integrity. With regard to virus infection, IAV-induced SUMOylation of CDC73 appears to potentiate its function in transcription elongation of genes promoting antiviral immunity.

DISCUSSION

The role of SUMO modifications in resolving cellular stress conditions and responding to DNA virus infections in the nucleus is well established (Everett et al., 2013; Hay, 2013). Here we report the induction of a host SUMOylation response to nuclear-replicating RNA viruses, exemplified by the important human and animal pathogens, influenza A and B viruses. For nuclear-replicating DNA viruses, the incoming naked DNA molecule has been suggested to trigger SUMOylation in a manner analogous to damaged cellular DNA (Cuchet-Lourenço et al., 2011). In our study, we found that active viral RNA polymerase function is a major trigger for SUMO remodeling, suggesting that this foreign activity in the nucleus induces a previously unappreciated form of stress to which the host raises a SUMO response. It is currently unknown which aspect of viral RNA polymerase activity might induce SUMO remodeling, although our unspliced replicon data suggest that viral hijack of the host RNA-splicing machinery is not a major stimulus. A key question to resolve will be whether IAV-induced SUMOylation is a specific response to infection or a generalized response to nuclear stress. For example, with parallels to cytoplasmic RIG-I, a nuclear pathogen sensor might be activated by newly synthesized IAV RNA to

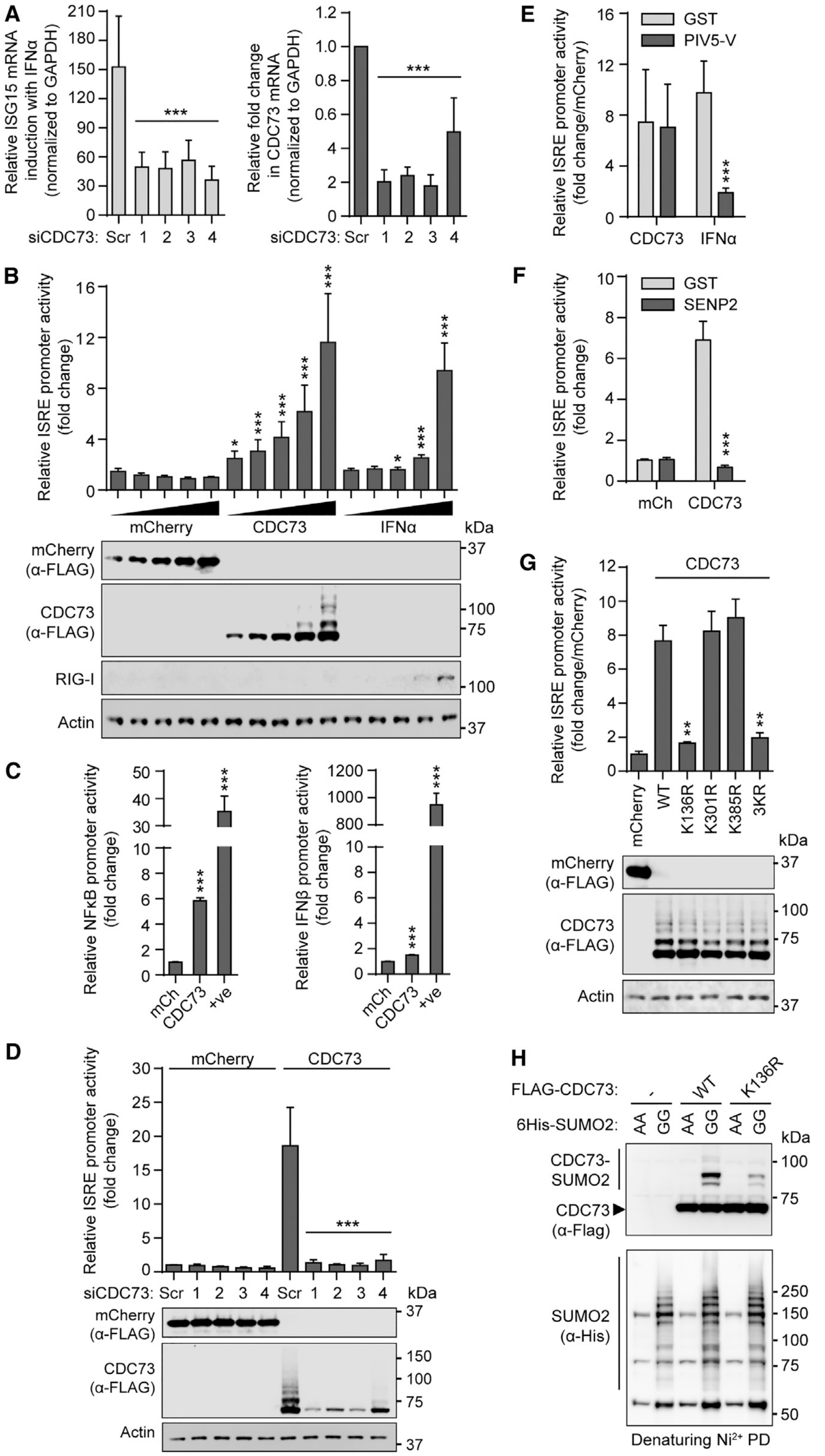

Figure 7. SUMO Modification Promotes the Function of CDC73 in Mediating an ISG Response

(A) Impact of CDC73 knockdown on ISG expression. A549s were transfected for 48 hr with four independent siRNAs targeting CDC73 (or scrambled) before stimulation with 100 IU/ml IFNα for 8 hr. The mRNA levels of CDC73, ISG15, and GAPDH were quantified. (Left) IFNα-mediated induction of ISG15 mRNA relative to mock is shown.

(B) Induction of an ISRE-containing promoter by overexpressed CDC73. 293Ts were co-transfected with expression plasmids encoding FLAG-tagged mCherry or CDC73 (12.5–200 ng) together with pGL3-Mx1P-FFluc and pRL-SV40. After 36 hr, FF luciferase activity was determined and normalized to *Renilla*. Parallel samples were harvested for western blot, probing for the indicated proteins.

(C) Impact of CDC73 overexpression on NF-κB (left) and IFNβ (right) promoters. 293Ts were co-transfected with FLAG-tagged mCherry or CDC73 (200 ng) together with pNF-κB-FFLuc (or p125-FFLuc) and pRL-SV40. Control for NF-κB promoter activation was 10 ng/ml TNF-α for 12 hr (+ve); control for IFNβ promoter activation was co-transfection of 20 ng RIG-I2CARD (+ve). After 36 hr, relative activity was determined as in (B).

(D) The siRNAs targeting CDC73 abrogate the effect of CDC73 overexpression on inducible gene expression. 293Ts were transfected/processed as in (B) except four independent siRNAs targeting CDC73 (or scrambled) also were transfected.

(E) CDC73-mediated induction of an ISRE-containing promoter is independent of STAT1 function. 293Ts were co-transfected with expression plasmids encoding FLAG-tagged mCherry or CDC73 (100 ng) together with plasmids encoding GST or PIV5-V (100 ng) and pGL3-Mx1P-FFluc and pRL-SV40. Control cells were stimulated with 100 IU/ml IFNα. Then, 36 hr post-transfection, relative activity was determined as in (B). Data represent fold induction in promoter activation relative to mCherry-expressing cells.

(F) CDC73-mediated induction of an ISRE-containing promoter is dependent on SUMOylation. Experiment was as in (E) except plasmids encoding GST or SENP2 (100 ng) were co-transfected.

(G) The K136 SUMOylation site in CDC73 is essential for stimulating inducible gene expression. Experiment was as in (B) but included a panel of FLAG-tagged CDC73 lysine mutants (wild-type [WT]; K136R; K301R; K385R; or a triple mutant, 3KR).

(H) K136 is a major SUMOylation site in CDC73. 293Ts were co-transfected with expression plasmids encoding FLAG-tagged CDC73-WT or CDC73-K136R, together with 6His-tagged SUMO2-GG or SUMO2-AA. Following denaturing Ni^{2+} pull-down, purified proteins were detected by western blot with anti-6His or anti-FLAG. For all graphs, bars represent mean values from triplicates (±SD) and are derived from three independent experiments. Statistical significance was determined using the Student's t test (*p < 0.01, **p < 0.001, and ***p < 0.0001; ns, non-significant). See also Figure S6.

stimulate SUMOylation. Alternatively, the cell could simply be reacting to infection-driven changes in the levels of specific host factors, such as ribonucleosides depleted by IAV polymerase activity or uncapped host RNAs generated by viral cap snatching. Given the close physical association of IAV RNPs with host chromosomes (Chase et al., 2011), it is possible that infection induces a non-canonical DNA damage-like SUMO response. In this regard, tethering of viral RNAs to chromatin may mimic aberrant RNA:DNA hybrids reminiscent of R loops, a situation in which SUMOylation plays an important resolving role (Richard et al., 2013).

Several studies have shown that SUMO is important for different aspects of IAV replication, predominantly by directly modifying viral proteins (Han et al., 2014; Santos et al., 2013; Wu et al., 2011; Xu et al., 2011). Experimentally assessing the global contribution of SUMOylation to virus infection is confounded by the integral nature of key SUMO components to cellular activities. For example, the sole SUMO E2 enzyme (Ubc9), several SUMO proteases, and SUMO2 itself are all essential for embryonic development (Kang et al., 2010; Nacerddine et al., 2005; Wang et al., 2014), and SUMO1-deficient mice are only viable due to functional compensation by SUMO2/3 (Evdokimov et al., 2008). Furthermore, depletion of Ubc9 is difficult to establish in tissue culture and results in extensive cell-cycle defects and loss of cell viability (Boutell et al., 2011). Thus, in this study, we rationally undertook to identify and characterize only the IAV-responsive SUMO proteome. Using quantitative proteomics, we revealed an IAV-driven reprogramming of host SUMOylation that is both quantitatively and qualitatively distinct from previously characterized SUMO stress responses. This dataset provided the framework for us to perform targeted functional analysis of a specific IAV-induced SUMO substrate, CDC73, without the need to deplete the entire SUMO system. Our data provide a resource to continue such studies with other IAV-induced SUMO substrates.

Proteins that change in SUMOylation status during IAV infection are involved in a diverse range of nuclear biological processes and regulatory pathways (Figure 3D; Table S7). Consistent with viral RNA polymerase activity triggering SUMO remodeling, we found that IAV infection retargets SUMO to many proteins involved in chromatin remodeling or RNA polymerase II transcription, including chromodomain DNA-binding helicases (CHD1, CHD2, and CHD8), the FACT complex (SSRP1 and SUPT16H), transcription initiation factors (TAF1 and TAF3), the PAF1 complex (PAF1, CTR9, RTF1, LEO1, and CDC73), and other transcription elongation factors (AFF4, EAF1, HTATSF1, IWS1, MLLT3, and SUPT5H). Several infection-induced SUMO targets also are involved in mRNA maturation events, such as 3′ end pre-mRNA processing (CPSF1, CPSF2, FIP1L1, RBBP6, and WDR33), splicing (CLASRP, SFPQ, and ZRANB2), and nuclear RNA quality control (ZC3H18, ZCCHC7, and PAPD5).

In addition, despite being unable to detect an IAV-induced canonical DNA damage response, we identified an infection-responsive increase in the SUMOylation of a remarkable number of host proteins that function in DNA damage repair, such as BLM, EME1, the SUMO E3 ligase PIAS4, the ubiquitin E3 ligases RAD18 and RNF111, and almost all members of the human SMC5/6 complex. Functional screening by ourselves and others has shown that a number of these IAV-induced SUMO targets are required for efficient IAV propagation (Karlas et al., 2010; Landeras-Bueno et al., 2011; Naito et al., 2007) or act as IAV restriction factors (Marazzi et al., 2012). Notably, we identified three members of the SMC5/6 complex (SMC5, SMC6, and the SUMO E3 ligase NSMCE2) as potential antiviral factors, indicating that DNA damage repair proteins may play additional roles in resolving IAV infection. These data suggest that nuclear RNA virus replication stress can be channelled into SUMO-dependent effector pathways shared with cellular DNA repair processes.

We identified all components of the PAF1 complex as IAV-triggered SUMO targets. PAF1 itself, as well as CTR9, recently has been implicated as a positive regulator of antiviral and pro-inflammatory gene expression (Marazzi et al., 2012; Parnas et al., 2015; Youn et al., 2007), and genetic deletion of the CDC73 component of PAF1C in mouse embryonic fibroblasts led to reduced expression levels of several known ISGs, including *Ddx58*, *Trim21*, *Mov10*, *Isg20*, *Stat2*, and *Bst2* (Wang et al., 2008). Here our mechanistic studies revealed a role for SUMOylation of CDC73 in promoting ISG expression. These data thereby directly link one consequence of the IAV-triggered SUMOylation response to antiviral defense. Given that CDC73 SUMOylation has been described to be important for its trafficking in response to proteotoxicity (Lamoliatte et al., 2014) and CDC73 SUMOylation also is enhanced by heat shock, we hypothesize a general function for stress-triggered CDC73 SUMOylation in regulating stress-inducible gene expression.

Our SUMO proteomic datasets and functional characterization now provide a platform to address the role of selected protein groups and their SUMO modification in the IAV replication cycle. This is complemented by existing proteomic studies that recently have mapped SUMO modification sites in most of the targets we identified here (Table S4). IAV infection causes a global reprogramming of the host SUMOylation landscape, the specific temporal dynamics of which have yet to be fully explored. The resources presented here will add a layer of post-translational understanding to previous transcriptomic, proteomic, and genome-wide depletion studies that have sought to gain insights into the extensive interplay between influenza viruses and their hosts (Josset et al., 2014; Karlas et al., 2010; König et al., 2010; Shapira et al., 2009).

EXPERIMENTAL PROCEDURES

Cells and Viruses

HEK 293T, A549, Vero, and MDCK cells were maintained in DMEM supplemented with 10% (v/v) fetal calf serum (FCS), 100 units/ml penicillin, and 100 μg/ml streptomycin (Life Technologies). MRC5 cells were maintained in Eagle's minimal essential medium (EMEM, Sigma) supplemented with 10% (v/v) FCS, 100 units/ml penicillin, 100 μg/ml streptomycin, 2 mM L-Glutamine, and 1% (v/v) Non-Essential Amino Acids (Life Technologies). Generation of A549 cells stably expressing TAP or TAP-SUMO proteins is described in the Supplemental Experimental Procedures. IAV (A/WSN/33) was propagated and titrated by standard plaque assay in MDCKs, while influenza B virus (B/Yamagata/88) and Sendai virus were propagated in 10-day-old embryonated chicken eggs. La Crosse encephalitis virus (LACV), vesicular stomatitis virus (VSV), and Semliki Forest virus (SFV) were propagated and titrated by plaque assay in Vero cells. UV inactivation of IAV was performed on ice with UV irradiation (254 nm) for 1 min at a distance of 7 cm. PCR-based analyses,

luciferase reporter assays, cell viability, and statistical methods are detailed in the Supplemental Experimental Procedures.

Immunodetection Analyses

For western blots, samples were lysed 1:1 in either 2× urea disruption buffer (6 M urea, 2 M β-mercaptoethanol, and 4% SDS) or 2× Laemmli's sample buffer, nucleic acids sheared by passing three times through a 29G needle, and boiled for 10 min prior to protein separation by SDS-PAGE on NuPAGE Novex 4%–12% Bis-Tris gradient gels (Life Technologies). Proteins were detected by western blotting following transfer to polyvinylidene fluoride (PVDF) membranes. Antibodies used, as well as immunofluorescence assays, are described in the Supplemental Experimental Procedures.

SILAC Cell Culture, Treatments, and TAP Purification

Proteomic experiments were performed using the SILAC technique that allows for quantitative data analysis. In brief, cells were grown in DMEM with L-lysine and L-arginine replaced with stable isotope forms (Cambridge Isotope Laboratories) in various combinations depending on treatment (see the Supplemental Experimental Procedures). SILAC DMEM was supplemented with 10% dialyzed FCS. The modified denaturing TAP procedure has been described previously (Golebiowski et al., 2010). In brief, after treatment, cells were washed with PBS and lysed with denaturing buffer containing 2% SDS. For large-scale mass spectrometry experiments with three conditions, all resulting lysates were mixed 1:1:1 (based on total protein), and a crude sample (~1% of the total) was analyzed separately from the remaining ~99% material, which was subjected to TAP purification (see the Supplemental Experimental Procedures). Both samples were resolved on NuPAGE Novex 10% Bis-Tris polyacrylamide gels prior to gel slice excision, in-gel tryptic digestion, and liquid chromatography-tandem mass spectrometry (LC-MS/MS). Detailed methods as well as information on data processing are included in the Supplemental Experimental Procedures. Small-scale purifications were done in essentially the same way, although lysates were handled separately and analyzed by SDS-PAGE and western blot.

shRNA Lentivirus Library Preparation and Functional Screening

A customized 129 component sequence-verified MISSION shRNA lentiviral plasmid (pLKO.1-puro) library targeting 44 genes of interest (as well as a negative control scramble sequence) (Table S6) was purchased from Sigma-Aldrich. Lentiviral stocks were prepared by co-transfecting each pLKO.1-puro plasmid with pMD2.G and pCMVdR8.91 into 293T cells using PEI. Lentiviral supernatants were harvested 60 hr post-transfection, aliquoted, and stored at −80°C. For screening gene depletion impact on IAV, A549 cells in 24-well plates were transduced with the appropriate lentivirus stock for 48 hr in the presence of 8 μg/ml polybrene (Millipore). Very high transduction efficiency for a subset of our lentiviral preparations was confirmed using puromycin treatment of parallel plates. Transduced cells were infected with WSN at an MOI of 0.001 plaque-forming units (PFU)/cell, and supernatants were collected and titrated by plaque assay at 24 and 48 hr.

ACCESSION NUMBERS

The mass spectrometry proteomic data have been deposited to the ProteomeXchange Consortium (Vizcaíno et al., 2014) via the PRIDE partner repository with the dataset identifier PXD002943.

SUPPLEMENTAL INFORMATION

Supplemental Information includes Supplemental Experimental Procedures, six figures, and seven tables and can be found with this article online at http://dx.doi.org/10.1016/j.celrep.2015.10.001.

AUTHOR CONTRIBUTIONS

P.D. designed, performed, and analyzed the cell biology and virology experiments. F.G. designed, performed, and analyzed the proteomic experiments. M.H.T. performed mass spectrometry and contributed to data analysis. A.M.L. generated new reagents. A.T. contributed to virology. R.T.H. designed experiments and interpreted results. B.G.H. led the project, designed experiments, interpreted results, and wrote the manuscript.

ACKNOWLEDGMENTS

We are grateful to the following for reagents and advice: R. Everett, C. Boutell, J. Pavlovic, S. Stertz, R. Randall, P. Palese, A. García-Sastre, G. Kochs, M. Schwemmle, M. Meyerson, and E. Yeh. M. Charman provided excellent technical assistance. The research leading to these results received funding from the European Research Council (ERC) under EU FP7 ERC Starting Grant Agreement 335809 (SUMOFLU), the MRC UK, and the European Commission under EU FP7 Marie Curie CIG 321703 (UBIFLU) (all to B.G.H.). B.G.H. was a Sir Henry Dale Fellow jointly funded by the Wellcome Trust and the Royal Society (100034/Z/12/Z). Work in the R.T.H. lab was supported by a Cancer Research UK programme grant (C434/A13067) and a Wellcome Trust Senior Investigator Award (098391/Z/12/Z).

Received: May 28, 2015
Revised: August 24, 2015
Accepted: September 28, 2015
Published: November 5, 2015

REFERENCES

Boutell, C., Cuchet-Lourenço, D., Vanni, E., Orr, A., Glass, M., McFarlane, S., and Everett, R.D. (2011). A viral ubiquitin ligase has substrate preferential SUMO targeted ubiquitin ligase activity that counteracts intrinsic antiviral defence. PLoS Pathog. *7*, e1002245.

Chase, G.P., Rameix-Welti, M.A., Zvirbliene, A., Zvirblis, G., Götz, V., Wolff, T., Naffakh, N., and Schwemmle, M. (2011). Influenza virus ribonucleoprotein complexes gain preferential access to cellular export machinery through chromatin targeting. PLoS Pathog. *7*, e1002187.

Chen, E.Y., Tan, C.M., Kou, Y., Duan, Q., Wang, Z., Meirelles, G.V., Clark, N.R., and Ma'ayan, A. (2013). Enrichr: interactive and collaborative HTML5 gene list enrichment analysis tool. BMC Bioinformatics *14*, 128.

Cuchet-Lourenço, D., Boutell, C., Lukashchuk, V., Grant, K., Sykes, A., Murray, J., Orr, A., and Everett, R.D. (2011). SUMO pathway dependent recruitment of cellular repressors to herpes simplex virus type 1 genomes. PLoS Pathog. *7*, e1002123.

Evdokimov, E., Sharma, P., Lockett, S.J., Lualdi, M., and Kuehn, M.R. (2008). Loss of SUMO1 in mice affects RanGAP1 localization and formation of PML nuclear bodies, but is not lethal as it can be compensated by SUMO2 or SUMO3. J. Cell Sci. *121*, 4106–4113.

Everett, R.D., Boutell, C., and Hale, B.G. (2013). Interplay between viruses and host sumoylation pathways. Nat. Rev. Microbiol. *11*, 400–411.

Fodor, E. (2013). The RNA polymerase of influenza a virus: mechanisms of viral transcription and replication. Acta Virol. *57*, 113–122.

Fritah, S., Lhocine, N., Golebiowski, F., Mounier, J., Andrieux, A., Jouvion, G., Hay, R.T., Sansonetti, P., and Dejean, A. (2014). Sumoylation controls host anti-bacterial response to the gut invasive pathogen Shigella flexneri. EMBO Rep. *15*, 965–972.

Golebiowski, F., Matic, I., Tatham, M.H., Cole, C., Yin, Y., Nakamura, A., Cox, J., Barton, G.J., Mann, M., and Hay, R.T. (2009). System-wide changes to SUMO modifications in response to heat shock. Sci. Signal. *2*, ra24.

Golebiowski, F., Tatham, M.H., Nakamura, A., and Hay, R.T. (2010). High-stringency tandem affinity purification of proteins conjugated to ubiquitin-like moieties. Nat. Protoc. *5*, 873–882.

Han, Q., Chang, C., Li, L., Klenk, C., Cheng, J., Chen, Y., Xia, N., Shu, Y., Chen, Z., Gabriel, G., et al. (2014). Sumoylation of influenza A virus nucleoprotein is essential for intracellular trafficking and virus growth. J. Virol. *88*, 9379–9390.

Hay, R.T. (2013). Decoding the SUMO signal. Biochem. Soc. Trans. *41*, 463–473.

Hendriks, I.A., D'Souza, R.C., Yang, B., Verlaan-de Vries, M., Mann, M., and Vertegaal, A.C. (2014). Uncovering global SUMOylation signaling networks in a site-specific manner. Nat. Struct. Mol. Biol. *21*, 927–936.

Hendriks, I.A., Treffers, L.W., Verlaan-de Vries, M., Olsen, J.V., and Vertegaal, A.C. (2015). SUMO-2 orchestrates chromatin modifiers in response to DNA damage. Cell Rep. *10*, 1778–1791.

Josset, L., Zeng, H., Kelly, S.M., Tumpey, T.M., and Katze, M.G. (2014). Transcriptomic characterization of the novel avian-origin influenza A (H7N9) virus: specific host response and responses intermediate between avian (H5N1 and H7N7) and human (H3N2) viruses and implications for treatment options. MBio *5*, e01102–e01113.

Kang, X., Qi, Y., Zuo, Y., Wang, Q., Zou, Y., Schwartz, R.J., Cheng, J., and Yeh, E.T. (2010). SUMO-specific protease 2 is essential for suppression of polycomb group protein-mediated gene silencing during embryonic development. Mol. Cell *38*, 191–201.

Karlas, A., Machuy, N., Shin, Y., Pleissner, K.P., Artarini, A., Heuer, D., Becker, D., Khalil, H., Ogilvie, L.A., Hess, S., et al. (2010). Genome-wide RNAi screen identifies human host factors crucial for influenza virus replication. Nature *463*, 818–822.

König, R., Stertz, S., Zhou, Y., Inoue, A., Hoffmann, H.H., Bhattacharyya, S., Alamares, J.G., Tscherne, D.M., Ortigoza, M.B., Liang, Y., et al. (2010). Human host factors required for influenza virus replication. Nature *463*, 813–817.

Lamoliatte, F., Caron, D., Durette, C., Mahrouche, L., Maroui, M.A., Caron-Lizotte, O., Bonneil, E., Chelbi-Alix, M.K., and Thibault, P. (2014). Large-scale analysis of lysine SUMOylation by SUMO remnant immunoaffinity profiling. Nat. Commun. *5*, 5409.

Landeras-Bueno, S., Jorba, N., Pérez-Cidoncha, M., and Ortín, J. (2011). The splicing factor proline-glutamine rich (SFPQ/PSF) is involved in influenza virus transcription. PLoS Pathog. *7*, e1002397.

Li, Z., Wu, S., Wang, J., Li, W., Lin, Y., Ji, C., Xue, J., and Chen, J. (2012). Evaluation of the interactions of HIV-1 integrase with small ubiquitin-like modifiers and their conjugation enzyme Ubc9. Int. J. Mol. Med. *30*, 1053–1060.

Marazzi, I., Ho, J.S., Kim, J., Manicassamy, B., Dewell, S., Albrecht, R.A., Seibert, C.W., Schaefer, U., Jeffrey, K.L., Prinjha, R.K., et al. (2012). Suppression of the antiviral response by an influenza histone mimic. Nature *483*, 428–433.

Nacerddine, K., Lehembre, F., Bhaumik, M., Artus, J., Cohen-Tannoudji, M., Babinet, C., Pandolfi, P.P., and Dejean, A. (2005). The SUMO pathway is essential for nuclear integrity and chromosome segregation in mice. Dev. Cell *9*, 769–779.

Naito, T., Kiyasu, Y., Sugiyama, K., Kimura, A., Nakano, R., Matsukage, A., and Nagata, K. (2007). An influenza virus replicon system in yeast identified Tat-SF1 as a stimulatory host factor for viral RNA synthesis. Proc. Natl. Acad. Sci. USA *104*, 18235–18240.

Nefkens, I., Negorev, D.G., Ishov, A.M., Michaelson, J.S., Yeh, E.T., Tanguay, R.M., Müller, W.E., and Maul, G.G. (2003). Heat shock and Cd2+ exposure regulate PML and Daxx release from ND10 by independent mechanisms that modify the induction of heat-shock proteins 70 and 25 differently. J. Cell Sci. *116*, 513–524.

Pal, S., Santos, A., Rosas, J.M., Ortiz-Guzman, J., and Rosas-Acosta, G. (2011). Influenza A virus interacts extensively with the cellular SUMOylation system during infection. Virus Res. *158*, 12–27.

Parnas, O., Jovanovic, M., Eisenhaure, T.M., Herbst, R.H., Dixit, A., Ye, C.J., Przybylski, D., Platt, R.J., Tirosh, I., Sanjana, N.E., et al. (2015). A genome-wide CRISPR screen in primary immune cells to dissect regulatory networks. Cell *162*, 675–686.

Ran, Y., Liu, T.T., Zhou, Q., Li, S., Mao, A.P., Li, Y., Liu, L.J., Cheng, J.K., and Shu, H.B. (2011). SENP2 negatively regulates cellular antiviral response by de-SUMOylating IRF3 and conditioning it for ubiquitination and degradation. J. Mol. Cell Biol. *3*, 283–292.

Ribet, D., Hamon, M., Gouin, E., Nahori, M.A., Impens, F., Neyret-Kahn, H., Gevaert, K., Vandekerckhove, J., Dejean, A., and Cossart, P. (2010). Listeria monocytogenes impairs SUMOylation for efficient infection. Nature *464*, 1192–1195.

Richard, P., Feng, S., and Manley, J.L. (2013). A SUMO-dependent interaction between Senataxin and the exosome, disrupted in the neurodegenerative disease AOA2, targets the exosome to sites of transcription-induced DNA damage. Genes Dev. *27*, 2227–2232.

Saitoh, H., and Hinchey, J. (2000). Functional heterogeneity of small ubiquitin-related protein modifiers SUMO-1 versus SUMO-2/3. J. Biol. Chem. *275*, 6252–6258.

Santos, A., Pal, S., Chacón, J., Meraz, K., Gonzalez, J., Prieto, K., and Rosas-Acosta, G. (2013). SUMOylation affects the interferon blocking activity of the influenza A nonstructural protein NS1 without affecting its stability or cellular localization. J. Virol. *87*, 5602–5620.

Shapira, S.D., Gat-Viks, I., Shum, B.O., Dricot, A., de Grace, M.M., Wu, L., Gupta, P.B., Hao, T., Silver, S.J., Root, D.E., et al. (2009). A physical and regulatory map of host-influenza interactions reveals pathways in H1N1 infection. Cell *139*, 1255–1267.

Tammsalu, T., Matic, I., Jaffray, E.G., Ibrahim, A.F., Tatham, M.H., and Hay, R.T. (2014). Proteome-wide identification of SUMO2 modification sites. Sci. Signal. *7*, rs2.

Tatham, M.H., Matic, I., Mann, M., and Hay, R.T. (2011). Comparative proteomic analysis identifies a role for SUMO in protein quality control. Sci. Signal. *4*, rs4.

Vizcaíno, J.A., Deutsch, E.W., Wang, R., Csordas, A., Reisinger, F., Ríos, D., Dianes, J.A., Sun, Z., Farrah, T., Bandeira, N., et al. (2014). ProteomeXchange provides globally coordinated proteomics data submission and dissemination. Nat. Biotechnol. *32*, 223–226.

Wang, P., Bowl, M.R., Bender, S., Peng, J., Farber, L., Chen, J., Ali, A., Zhang, Z., Alberts, A.S., Thakker, R.V., et al. (2008). Parafibromin, a component of the human PAF complex, regulates growth factors and is required for embryonic development and survival in adult mice. Mol. Cell. Biol. *28*, 2930–2940.

Wang, L., Wansleeben, C., Zhao, S., Miao, P., Paschen, W., and Yang, W. (2014). SUMO2 is essential while SUMO3 is dispensable for mouse embryonic development. EMBO Rep. *15*, 878–885.

Wu, C.Y., Jeng, K.S., and Lai, M.M. (2011). The SUMOylation of matrix protein M1 modulates the assembly and morphogenesis of influenza A virus. J. Virol. *85*, 6618–6628.

Xu, K., Klenk, C., Liu, B., Keiner, B., Cheng, J., Zheng, B.J., Li, L., Han, Q., Wang, C., Li, T., et al. (2011). Modification of nonstructural protein 1 of influenza A virus by SUMO1. J. Virol. *85*, 1086–1098.

Yin, Y., Seifert, A., Chua, J.S., Maure, J.F., Golebiowski, F., and Hay, R.T. (2012). SUMO-targeted ubiquitin E3 ligase RNF4 is required for the response of human cells to DNA damage. Genes Dev. *26*, 1196–1208.

Youn, M.Y., Yoo, H.S., Kim, M.J., Hwang, S.Y., Choi, Y., Desiderio, S.V., and Yoo, J.Y. (2007). hCTR9, a component of Paf1 complex, participates in the transcription of interleukin 6-responsive genes through regulation of STAT3-DNA interactions. J. Biol. Chem. *282*, 34727–34734.

Cell Reports
Supplemental Information

Global Reprogramming of Host SUMOylation during Influenza Virus Infection

Patricia Domingues, Filip Golebiowski, Michael H. Tatham, Antonio M. Lopes, Aislynn Taggart, Ronald T. Hay, and Benjamin G. Hale

Figure S1

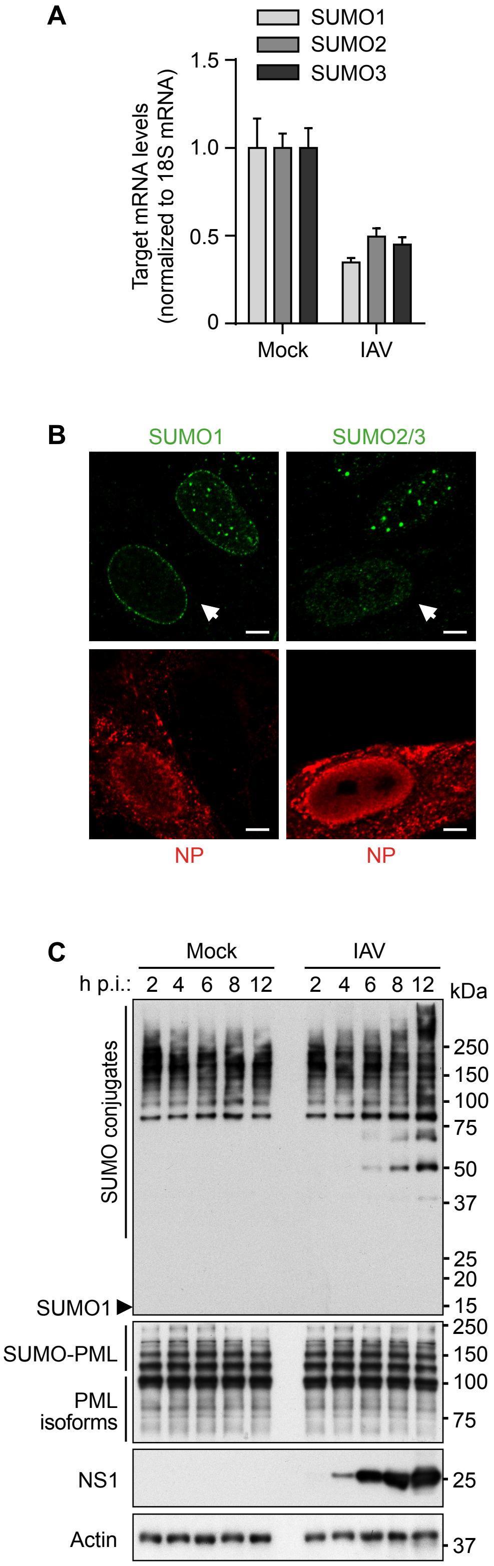
A
SUMO1
SUMO2
SUMO3
Target mRNA levels (normalized to 18S mRNA)
1.5
1.0
0.5
0
Mock
IAV
B
SUMO1
SUMO2/3
NP
NP
C
Mock
IAV
h p.i.: 2 4 6 8 12 2 4 6 8 12
kDa
SUMO conjugates
SUMO1
250
150
100
75
50
37
25
20
15
SUMO-PML
PML isoforms
250
150
100
75
NS1
25
Actin
37

Figure S1, related to Figure 1. Induction of SUMO conjugates by IAV infection does not correlate with changes in SUMO mRNA levels and shows target specificity. (**A**) RT-qPCR analysis of SUMO paralogue mRNA levels in A549 cells following infection with IAV at an MOI of 5 PFU/cell (or mock). Bars represent mean values, and error bars standard deviation of three independent experiments. (**B**) Immunofluorescent analysis of MRC5 cells infected with IAV at an MOI of 0.1 PFU/cell for 12h. SUMO1, or SUMO2/3, and NP were visualized after staining with specific antibodies. An uninfected and infected cell is shown in each panel for comparison of the 'SUMO foci' and 'SUMO diffuse' phenotypes. Scale bars represent 5µm. (**C**) Western blot analysis of whole-cell lysates from MRC5 cells infected with IAV at an MOI of 5 PFU/cell for the times indicated. SUMO1, PML, NS1, and actin were detected with specific antibodies.

Figure S2

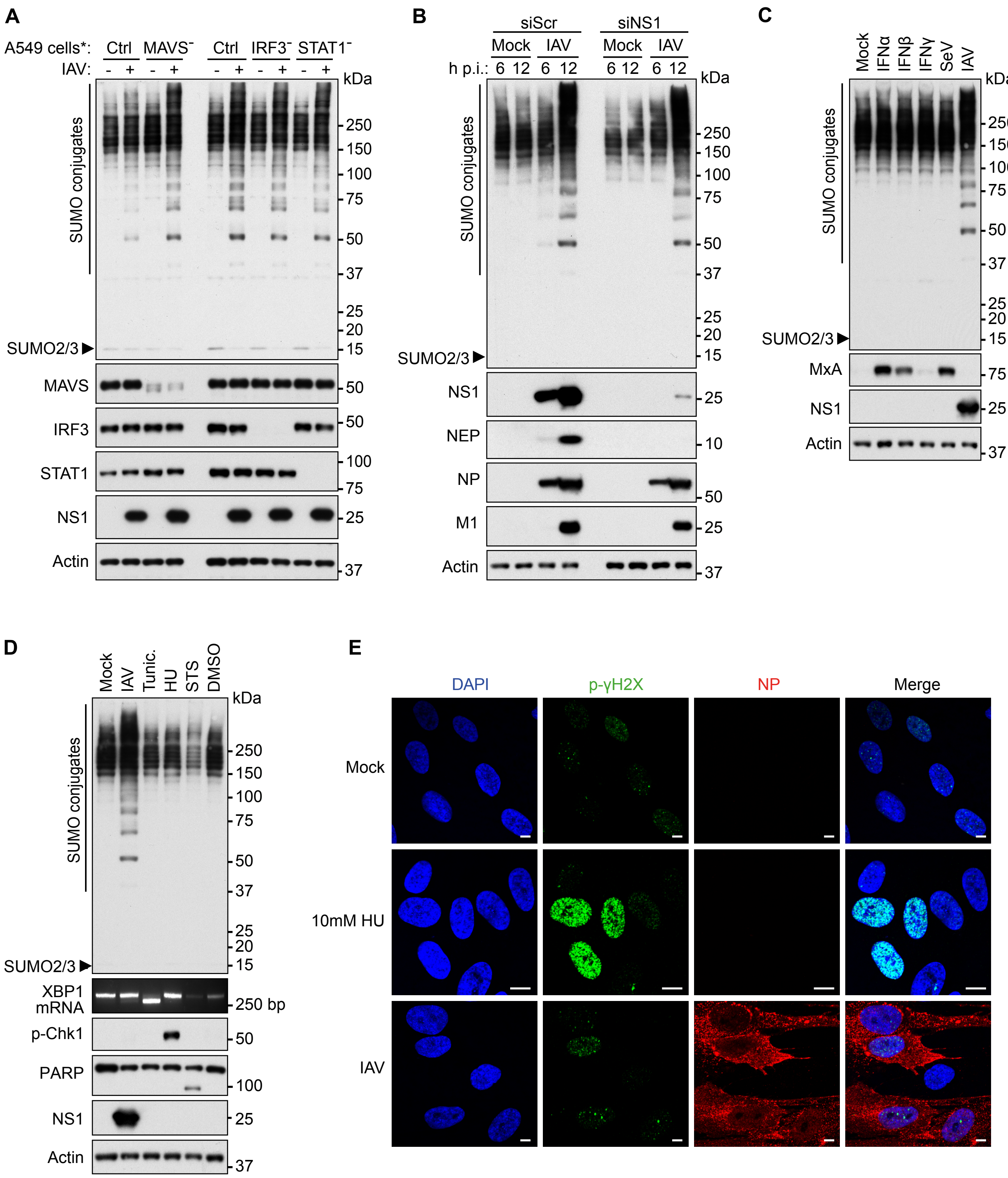
A
A549 cells*: Ctrl MAVS⁻ Ctrl IRF3⁻ STAT1⁻
IAV: - + - + - + - + - +
kDa
SUMO conjugates
250
150
100
75
50
37
25
20
15
SUMO2/3
MAVS
IRF3
STAT1
NS1
Actin
B
siScr siNS1
Mock IAV Mock IAV
h p.i.: 6 12 6 12 6 12 6 12
NEP
NP
M1
10
C
Mock IFNα IFNβ IFNγ SeV IAV
MxA
D
Mock IAV Tunic. HU STS DMSO
XBP1 mRNA
250 bp
p-Chk1
PARP
E
DAPI
p-γH2X
NP
Merge
Mock
10mM HU
IAV

Figure S2, related to Figure 2. Canonical interferon, ER stress, apoptotic and DNA-damage response pathways do not correlate with IAV-triggered SUMOylation. (**A**) A549 cell-lines stably expressing HCV NS3/4A (cleaves MAVS), BVDV-NPro (degrades IRF3) or PIV5-V (degrades STAT1) were infected with IAV at an MOI of 10 PFU/cell for 18h. Following total-cell lysis, western blot analysis was performed to detect SUMO2/3, MAVS, IRF3, STAT1, NS1 and actin. (**B**) siRNAs targeting NS1/NEP were transiently transfected into A549s 48h prior to infection with IAV at an MOI of 5 PFU/cell for the indicated time. Following total-cell lysis, western blot analysis was performed to detect SUMO2/3, NS1, NEP, NP, M1 and actin. (**C**) A549 cells were treated with 200IU/ml IFNα, IFNβ or IFNγ, or infected with SeV or IAV at an MOI of 10 PFU/ml, for 18h. Following total-cell lysis, western blot analysis was performed to detect SUMO2/3, MxA, NS1 and actin. (**D**) Impact of stimulating ER stress, apoptosis and DNA-damage on SUMOylation. A549 cells were infected with IAV at an MOI of 5 PFU/cell or treated with different drug compounds to activate ER stress (Tunicamycin, Tunic.; 1μg/ml), DNA damage (Hydroxyurea, HU; 10mM) or apoptosis (Staurosporine, STS; 1μM). At 12h post infection/treatment, total cell lysates or total RNA were analyzed by western blot to detect SUMO2/3, phospho-Chk1 (marker for DNA damage), PARP (PARP cleavage marker of apoptosis), NS1, or actin protein levels, or RT-PCR to detect XBP1 splicing (marker of ER stress). (**E**) Immunofluorescent analysis of MRC5 cells stimulated with 10mM hydroxyurea for 12h, or infected with IAV for 12h. Cells were stained for phospho-histone H2AX (marker for DNA damage), NP and DNA (DAPI). Scale bars represent 10μm.

Figure S3

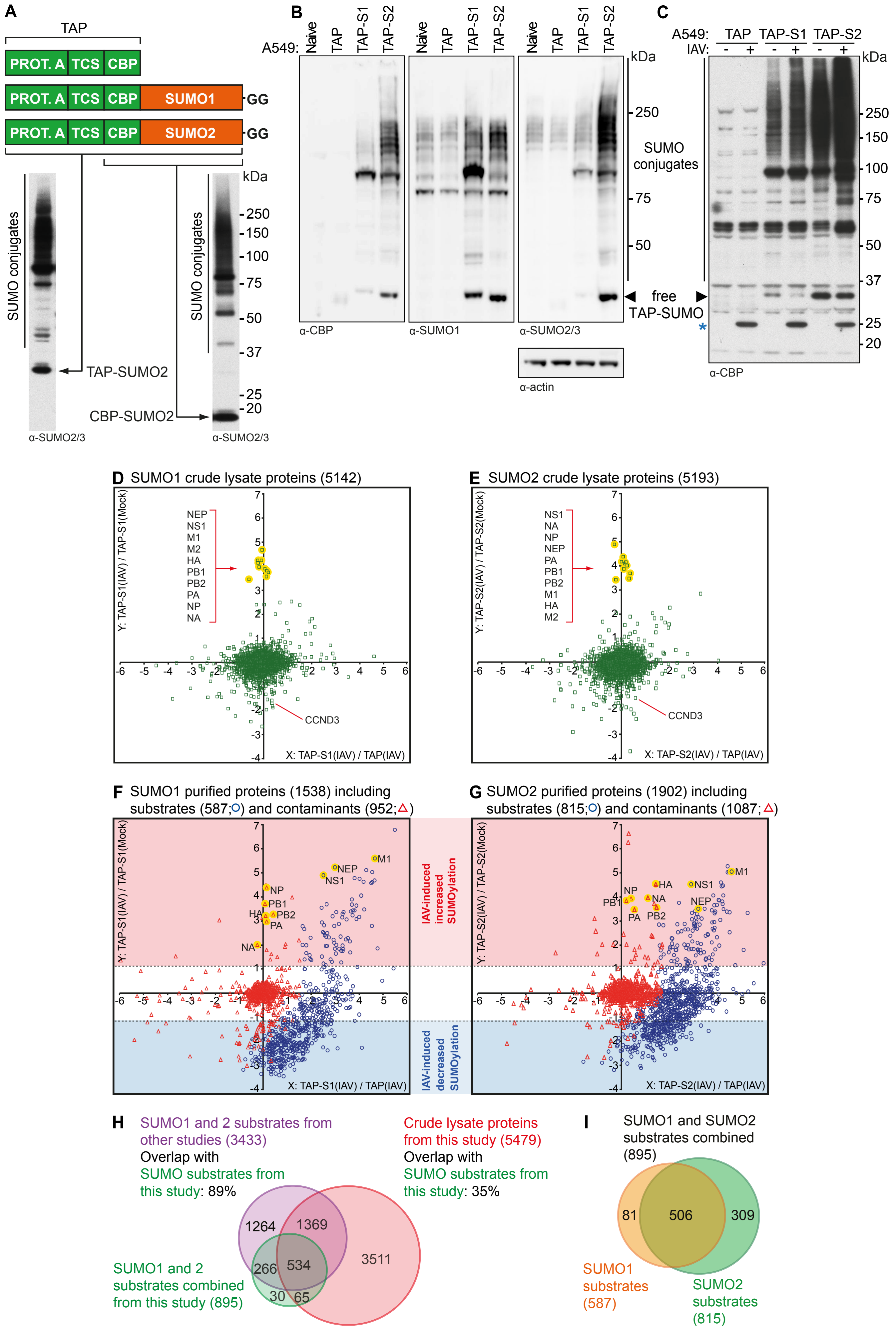

A
TAP
PROT. A TCS CBP
SUMO1
SUMO2
GG
SUMO conjugates
TAP-SUMO2
CBP-SUMO2
kDa
α-SUMO2/3
B
A549:
Naive
TAP
TAP-S1
TAP-S2
α-CBP
α-SUMO1
α-SUMO2/3
α-actin
SUMO conjugates
free TAP-SUMO
C
A549: TAP TAP-S1 TAP-S2
IAV: - + - + - +
α-CBP
D SUMO1 crude lysate proteins (5142)
Y: TAP-S1(IAV) / TAP-S1(Mock)
X: TAP-S1(IAV) / TAP(IAV)
NEP
NS1
M1
M2
HA
PB1
PB2
PA
NP
NA
CCND3
E SUMO2 crude lysate proteins (5193)
Y: TAP-S2(IAV) / TAP-S2(Mock)
X: TAP-S2(IAV) / TAP(IAV)
F SUMO1 purified proteins (1538) including substrates (587;○) and contaminants (952;△)
G SUMO2 purified proteins (1902) including substrates (815;○) and contaminants (1087;△)
IAV-induced increased SUMOylation
IAV-induced decreased SUMOylation
H
SUMO1 and 2 substrates from other studies (3433)
Overlap with SUMO substrates from this study: 89%
Crude lysate proteins from this study (5479)
Overlap with SUMO substrates from this study: 35%
SUMO1 and 2 substrates combined from this study (895)
1264
1369
3511
266
534
30
65
I
SUMO1 and SUMO2 substrates combined (895)
81
506
309
SUMO1 substrates (587)
SUMO2 substrates (815)

Figure S3, related to Figure 3. Quantitative SUMO proteomics of IAV-infected cells. (**A-C**) Generation and characterization of A549 cells stably expressing TAP, TAP-SUMO1 or TAP-SUMO2. (**A**) Schematic representation of the TAP tag and the TAP-SUMO constructs, together with representative western blots (anti-SUMO2/3) of total-cell lysate from cells stably expressing TAP-SUMO2 (left panel) and TAP-SUMO2 purified material (right panel) to indicate motility of protein constructs and conjugates before and after TEV cleavage. Abbreviations: PROT. A: protein A; TCS: TEV cleavage site; CBP: calmodulin binding protein. (**B**) Western blot analysis of SUMO1 and SUMO2/3 total protein expression levels in naïve A549 cells and A549s stably expressing TAP only, TAP-SUMO1 or TAP-SUMO2. (**C**) A549 cells stably expressing TAP, TAP-SUMO1 or TAP-SUMO2 were infected (or mock) with IAV at an MOI of 2 PFU/cell for 10h and total-cell lysates were analyzed by western blot with an anti-CBP antibody. Asterisk indicates a non-specific band that is present in infected cell lysates only. (**D-G**) **tsMAPs of all SUMO proteomics data.** (**D & E**) tsMAPs of normalized crude sample data for TAP-SUMO1 (**D**) and TAP-SUMO2 (**E**) experiments as detailed in figure 3 indicating log2-fold changes in total protein abundance following IAV infection (y-axis), or between the TAP- and TAP-SUMO A549 cell lines (x-axis). IAV proteins are highlighted with yellow background and named, as is cyclin D3 (CCND3), a host protein previously described to decrease in abundance during IAV infection (Zhang et al., 2011). (**F & G**) tsMAPs of normalized purified sample data for TAP-SUMO1 (**F**) and TAP-SUMO2 (**G**) experiments indicating log2-fold changes in protein modification following IAV infection (y-axis), or between the TAP- and TAP-SUMO A549 cell-lines (x-axis). Contaminant proteins (e.g. non-specifically bound proteins from all conditions, or external contaminants, such as keratins) are indicated with red triangles and SUMO

substrates with blue circles. IAV proteins are highlighted with yellow background and named. (**H**) Venn diagram showing high overlap in the SUMO substrates identified in this study with those identified in other studies, and low overlap with proteins identified in general crude lysates, suggesting high enrichment of SUMO conjugates. (**I**) Venn diagram showing the overlap in SUMO substrates identified in the two independent SUMO1 and SUMO2 experiments.

Figure S4

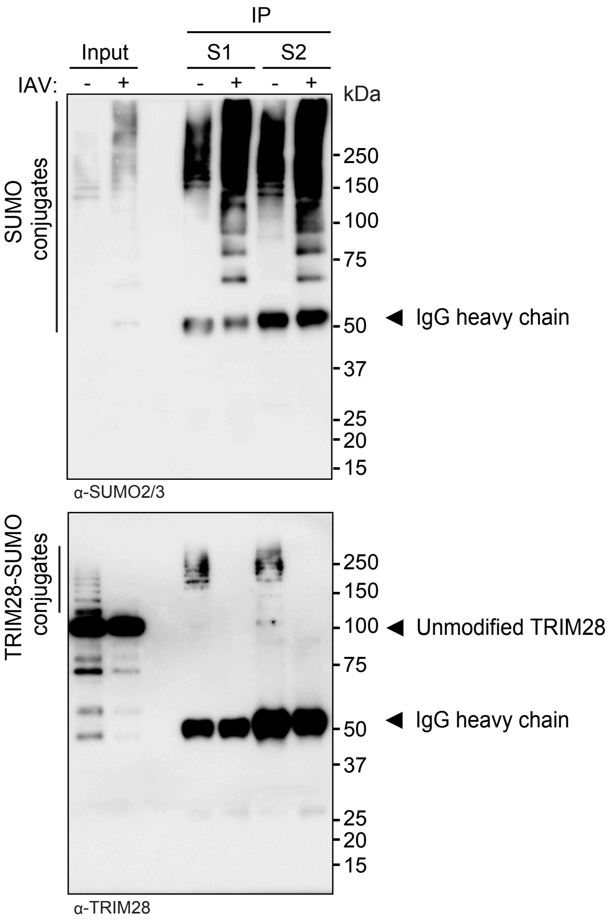
IP
Input
S1
S2
IAV: - + - + - +
kDa
SUMO conjugates
250
150
100
75
50
37
25
20
15
IgG heavy chain
α-SUMO2/3
TRIM28-SUMO conjugates
250
150
100
75
50
37
25
20
15
Unmodified TRIM28
IgG heavy chain
α-TRIM28

Figure S4, related to Figure 4. Validation of IAV-induced deSUMOylation of endogenous TRIM28. A549 cells were infected with IAV at an MOI of 5 PFU/cell for 16h. Western blot analysis was performed on both total-cell lysates and SUMO immunoprecipitates to detect SUMO2/3 and TRIM28.

Figure S5

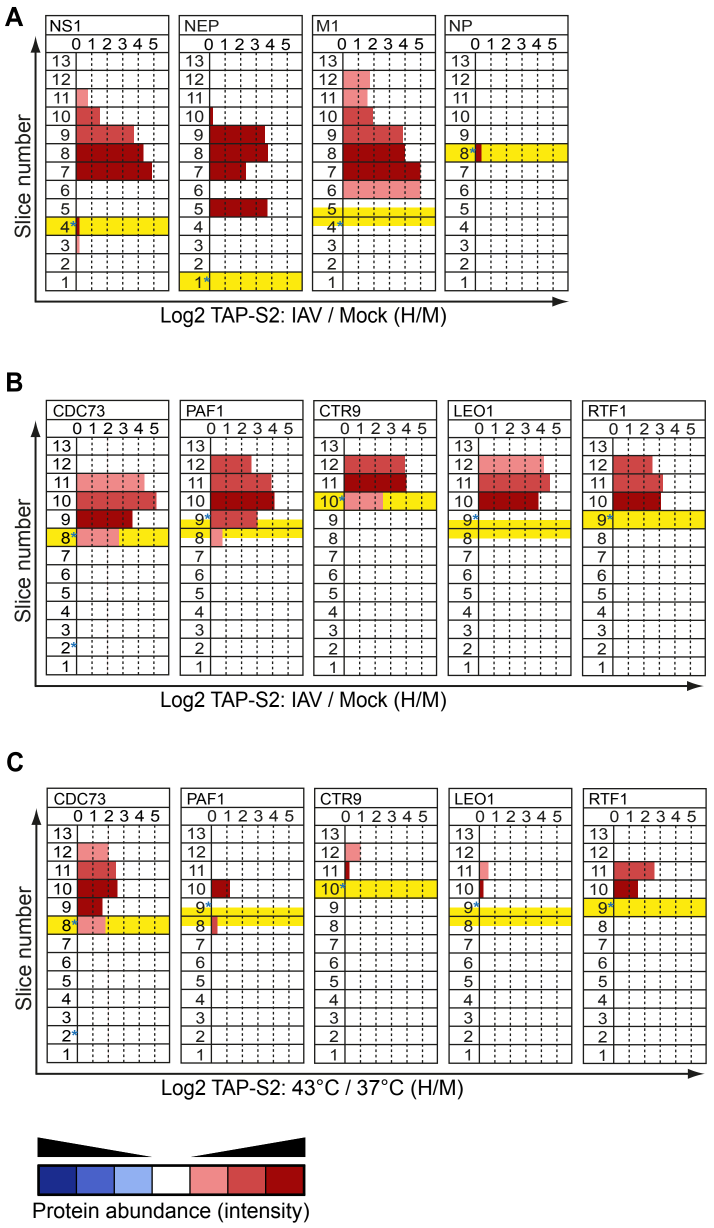
A
NS1
NEP
M1
NP
0 1 2 3 4 5
Slice number
Log2 TAP-S2: IAV / Mock (H/M)
B
CDC73
PAF1
CTR9
LEO1
RTF1
Log2 TAP-S2: IAV / Mock (H/M)
C
CDC73
PAF1
CTR9
LEO1
RTF1
Log2 TAP-S2: 43°C / 37°C (H/M)
Protein abundance (intensity)

Figure S5, related to Figures 4 & 5. Analysis of electrophoretic mobility shifts as a result of SUMO modification. (A-B) Predicted molecular weight (preMW) of substrates (unmodified) was compared to their observed electrophoretic mobility (obsEM) across all 13 slices (crude and TAP-SUMO2 purified, IAV/Mock). Yellow background indicates slice where unmodified protein would be expected based on its preMW. Bar length indicates change in protein ratio between IAV- and Mock- infected (H/M) purified TAP-SUMO2 samples. Intensity of color indicates protein abundance (intensity) in each slice: red nominally given to increased H/M ratios while blue nominally given to decreased H/M ratios. Asterisks indicate obsEM of specified proteins in crude lysate. (**A**) Analysis of NS1, NEP and M1 viral proteins confirms their annotation as SUMO substrates. NP analysis suggests it is a contaminant. (**B**) Analysis of PAF1 complex proteins in IAV-infected TAP-SUMO2 cells confirms their annotation as SUMO targets with increased modification following infection. (**C**) Analysis of PAF1 complex proteins in heat-shock stimulated TAP-SUMO2 cells – increased SUMOylation of CDC73 and RTF1 can be readily detected.

Figure S6

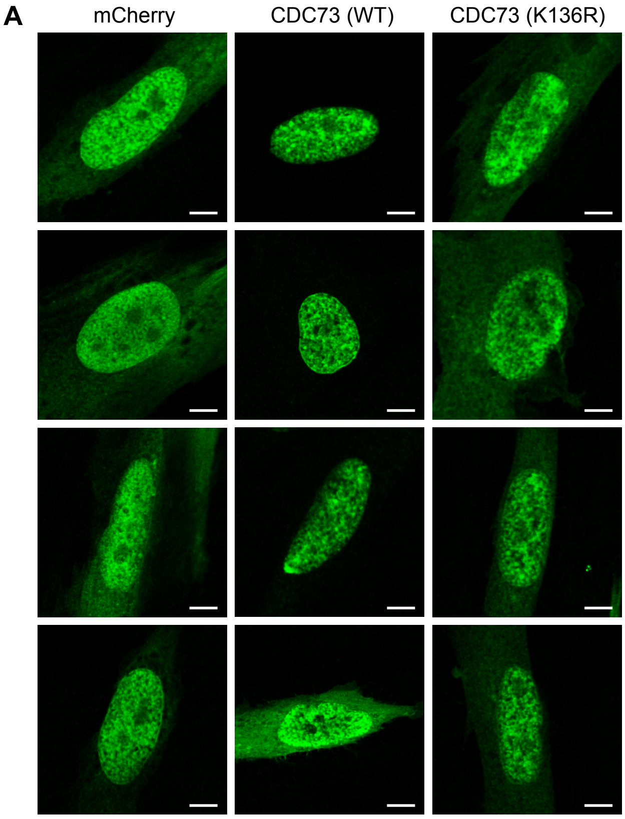
A
mCherry
CDC73 (WT)
CDC73 (K136R)

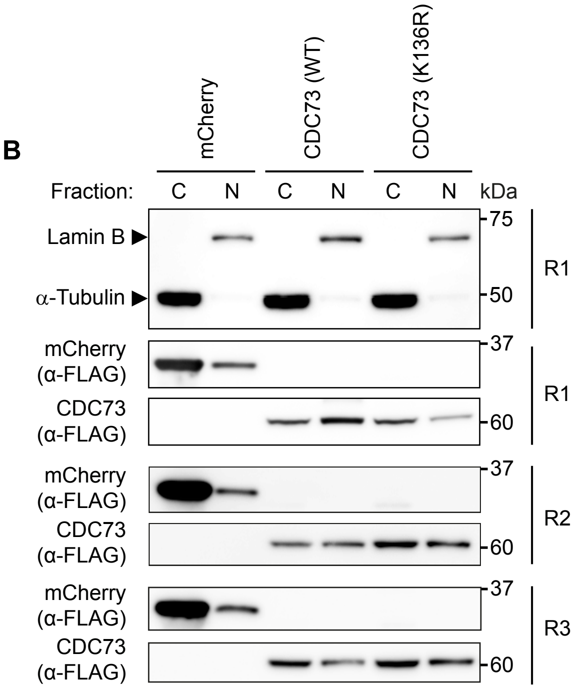
B
mCherry
CDC73 (WT)
CDC73 (K136R)
Fraction: C N C N C N
kDa
Lamin B
α-Tubulin
75
50
R1
mCherry (α-FLAG)
CDC73 (α-FLAG)
37
60
R1
mCherry (α-FLAG)
CDC73 (α-FLAG)
37
60
R2
mCherry (α-FLAG)
CDC73 (α-FLAG)
37
60
R3

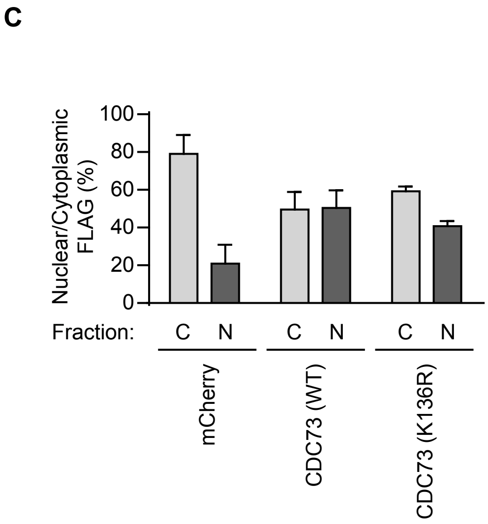
C
Nuclear/Cytoplasmic FLAG (%)
100
80
60
40
20
0
Fraction: C N C N C N
mCherry
CDC73 (WT)
CDC73 (K136R)

Figure S6, related to Figure 7. Intracellular distribution of CDC73 and CDC73-K136R. (**A**) Immunofluorescent analysis (anti-FLAG) of MRC5 cells transiently expressing FLAG-tagged mCherry, FLAG-tagged CDC73-WT or FLAG-tagged CDC73-K136R. Four representative images are shown. Scale bars represent 5μm. (**B**) Western blot analysis of nuclear (N) and cytoplasmic (C) fractions prepared from 293T cells transiently expressing FLAG-tagged mCherry, FLAG-tagged CDC73-WT or FLAG-tagged CDC73-K136R. Proteins were detected with the indicated antibodies. The experiment was performed three independent times: for the FLAG-tagged proteins of interest, each replicate is shown (R1-R3); for the Lamin B and α-Tubulin controls, a representative image (R1) is shown. (**C**) Quantification of the three independent replicates from (**B**). Bars represent mean values with standard deviations indicated.

Table S1, related to Figure 3. SUMO1 proteomic dataset in uninfected and IAV-infected A549s.

Table S2, related to Figure 3. SUMO2 proteomic dataset in uninfected and IAV-infected A549s.

Table S3, related to Figure 3. Summary of common and unique SUMO targets in A549s, and the impact of IAV infection.

Table S4, related to Figure 3. Summary of common SUMO targets in A549s and the overlap with previous SUMO site-specific proteomic mapping studies.

Table S5, related to Figure 5. SUMO2 proteomic dataset in unstimulated and heat-shocked A549s.

Table S6, related to Figure 6. Primary shRNA screening data and validations for the role of host SUMO targets during IAV infection.

Table S7, related to Figure 3. Enrichr pathway analysis for functional classification of host substrates that change in SUMOylation during IAV infection and other stresses.

SUPPLEMENTAL EXPERIMENTAL PROCEDURES

Cell-Lines and Compounds.

To generate A549 cells stably expressing TAP or TAP-SUMO proteins, sequences encoding TAP, TAP-SUMO1 or TAP-SUMO2 (ending in the C-terminal GG residues required for conjugation) were subcloned into the *Xho*I and *Xba*I sites of pEFIRES-P (Hobbs et al., 1998) to create pEFIRES-P/TAP, pEFIRES-P/TAP-SUMO1 or pEFIRES/TAP-SUMO2 expression vectors, respectively. Each construct was transfected into A549 cells, and individual clones stably expressing the desired proteins were selected using puromycin (1μg/ml) and validated by western blot. A549 cells stably expressing BVDV-NPro, PIV5-V or HCV-NS3/4A have been described previously (Chen et al., 2010; Hale et al., 2009; Killip et al., 2013), and were generously provided by Rick Randall (University of St. Andrews, UK). Compounds used include: cycloheximide (Sigma, 50μg/ml), leptomycin B (Enzo Life Sciences, 11nM), zanamivir (Redx Pharma, 10μM), tunicamycin (Sigma, 1μg/ml), hydroxyurea (Sigma, 10mM), and staurosporine (Sigma, 1μM). Interferon (IFN) alpha, beta and gamma were purchased from Merck, Calbiochem and Roche, respectively, and used as indicated. TNFα was purchased from Prepotech and used at 10ng/ml. Cell viability was assessed using CellTiter-Glo® Luminescent Cell Viability Assay (Promega), according to the manufacturer's instructions. Nuclear-cytoplasmic fractionation was performed essentially as described (Sunters et al., 2010; Trilling et al., 2014).

Immunofluorescence.

Cells on 13 mm coverslips were fixed, permeabilized and stained as described (Boutell et al., 2011). Antibodies used are detailed below. DNA was stained using DAPI. Images were visualized on a Zeiss LSM 710 confocal microscope. For the mini-replicon reporter assay, pPolI-358-FFluc and pPolI-358-mCherry were generated in a manner similar to previous descriptions (Hoffmann et al., 2008), and pCAGGS expression vectors encoding WSN PA, PB1, PB2 and NP, as well as pDZ-NP(WSN), have been described (Quinlivan et al., 2005; Zhang et al., 2012). pCAGGS expression vectors encoding KAN-1 PB1, PB1-E445A/E446A, AvianPr-PB2-E627K, PA and NP were kindly provided by Martin Schwemmle (University of Freiburg, Germany) (Manz et al., 2012). pmCherry-C1 was purchased from Clontech. Transfections were performed using FuGENE HD (Promega), with the ratio PA:PB1:PB2:NP:reporter being 1:1:1:10:10.

Antibodies.

Antibodies used for western blotting were: SUMO1 (Abcam, ab32058), SUMO2/3 (Abcam, ab53194), TRIM28 (Bethyl, A300-274A), CDC73 (Abcam, ab70533), UBTF (Santa Cruz, sc9131), ATRX (Santa Cruz, sc-15408), RanGAP1 (Life Technologies, 19C7), PML (Bethyl, A301-167A), CBP (Millipore, 07-482), actin (Sigma, A2103), IAV-NS1 (rabbit 1-73 (Solorzano et al., 2005)), IBV-NP (Abcam, ab20711), IAV-M1 (Abcam, ab22396), LACV-N (Reichelt et al., 2004), VSV-N (polyclonal anti-serum a gift from Jovan Pavlovic, University of Zurich, Switzerland), SFV-C (Landis et al., 1998), IAV-NP (Solorzano et al., 2005), FLAG (Sigma, F1804), RIG-I (Baum et al., 2010), NEP (Genescript, A01499), IRF3 (Santa Cruz, sc-9082), STAT1 (Santa Cruz, sc-417), MAVS (a gift from John McLauchlan, University of Glasgow, UK), phospho-Chk1 (Cell Signaling, 2348), PARP (Cell Signaling, 9542), MxA (Santa Cruz, sc-50509), 6His

(Abcam, ab18184), α-tubulin (Sigma, T6074) and Lamin B (Santa Cruz, sc-6216).

Antibodies used for immunofluorescence were: IAV-NP (HT103 (O'Neill et al., 1998)), IAV-PB1 (cc11, a gift from Silke Stertz), IAV-PB2 (polyclonal anti-serum a gift from Silke Stertz), IAV-PA (1J6, a gift from Silke Stertz), PML (Bethyl, A301-167A), hDaxx (Upstate, 07-471), Sp100 (a gift from Roger D. Everett, University of Glasgow, UK), SUMO1 (Enzo Lifesciences, BML-PW0505), SUMO2 (Enzo Lifesciences, BML-PW0510), FLAG (Sigma, F1804), phospho-histone H2AX (Cell Signaling, 9718).

SILAC Cell Culture.

Each experiment consisted of three SILAC conditions: L (light), where A549/TAP cells were grown in DMEM containing isotopically 'normal' amino acids (L-lysine, L-arginine); M (medium), where A549/TAP-SUMO1 (or SUMO2) cells were grown in DMEM containing 4,4,5,5-D_4 lysine and $^{13}C_6$ arginine; and H (heavy), where A549/TAP-SUMO1 (or SUMO2) cells were grown in DMEM containing $^{13}C_6$ $^{15}N_2$ lysine and $^{13}C_6$ $^{15}N_4$ arginine. Cells were grown in 15cm Petri dishes, with 20 dishes per SILAC condition. In both SUMO1 and SUMO2 IAV experiments, cells in condition L and H were infected with IAV for 10h at an MOI of 2 PFU/cell, and cells in condition M were mock-infected. In the SUMO2 heat-shock experiment, cells in condition L and M were maintained at 37°C, while cells in condition H were subjected to heat-shock (43°C) for 30 minutes.

TAP Procedure and Mass Spectrometry.

Lysates were diluted 25x in order to dilute out the denaturing 2% SDS and passed over IgG sepharose (GE Healthcare), which was followed by enzymatic removal of the Protein A portion of the TAP-tag (see **Fig. S3A**) using TEV protease (Promega). The resulting eluate was then affinity purified on calmodulin sepharose (GE Healthcare) followed by protein elution with buffer containing 10 mM EGTA, and protein recovery by precipitation with 100% TCA (w/v) and acetone washing. Purification resulted in ~25μg of protein sample that was resuspended in 30μl of 2x LDS sample buffer (Invitrogen). Crude sample (~50μg) was also mixed 1:1 with 2x LDS sample buffer. Both purified and crude samples were resolved on NuPAGE Novex 10% Bis-Tris polyacrylamide gels using MOPS buffer (Invitrogen). Gel-fractionated proteins were stained with Coommassie blue and the gel was sliced into 13 sections as outlined in **Fig. 3**. Protein slices were subjected to in-gel digestion with trypsin (Promega) essentially as described previously (Shevchenko et al., 2006). The resulting peptide mixtures were vacuum-dried and resuspended in 30μl of 1% formic acid prior to analysis by LC-MS/MS on a Q Exactive mass spectrometer (Thermo Scientific) coupled to an EASY-nLC 1000 liquid chromatography system via an EASY-Spray ion source (Thermo Scientific) running at 75 μm x 500 mm at 45ºC on an EASY-Spray column. An elution gradient duration of 240 min was used. Data were acquired in the data-dependent mode. Full scan spectra (*m/z* 300-1800) were acquired with resolution R = 70,000 at *m/z* 400 (after accumulation to a target value of 1,000,000 with maximum injection time of 20 ms). The 10 most intense ions were fragmented by HCD and measured with a target value of 500,000, maximum injection time of 60 ms and intensity threshold of 1.7e3. A 40 second dynamic exclusion list was applied.

Quantitative Mass Spectrometry Data Analyses.

MaxQuant Analyses.

All raw files generated by MS analysis were processed with MaxQuant software (version 1.3.0.5) (Cox and Mann, 2008) and searched against a FASTA database consisting of UniProtKB human and influenza A virus (A/WSN/1933(H1N1)) reference proteomes (both current as of June 2013). Two separate MaxQuant analyses were performed for each of the two IAV experiments. The first analysis aimed to determine relative changes to SUMOylation of all substrates ('global') - this included the data from all 13 TAP-purified sample slices and all 13 corresponding crude-lysate sample slices. The second analysis ('slice-by-slice') was designed to evaluate changes to SUMOylation in each individual sample slice, thus allowing definition of abundance (and thereby ratio) of every protein within the slice. The MaxQuant parameters used are below (asterisks denote non-default settings):

Parameter	Value
Version	1.3.0.5
Fixed modifications	Carbamidomethyl (C)
Randomize	FALSE
Special AAs	KR
Include contaminants	TRUE
MS/MS tol. (FTMS)	20 ppm
Top MS/MS peaks per 100 Da. (FTMS)	10
MS/MS deisotoping (FTMS)	TRUE
MS/MS tol. (ITMS)	0.5 Da
Top MS/MS peaks per 100 Da. (ITMS)	6
MS/MS deisotoping (ITMS)	FALSE
MS/MS tol. (TOF)	0.1 Da
Top MS/MS peaks per 100 Da. (TOF)	10
MS/MS deisotoping (TOF)	TRUE

MS/MS tol. (Unknown)	0.5 Da
Top MS/MS peaks per 100 Da. (Unknown)	6
MS/MS deisotoping (Unknown)	FALSE
Peptide FDR	0.01
Max. peptide PEP	1
Protein FDR	0.01
Site FDR	0.01
Use Normalized Ratios For Occupancy	TRUE
Apply site FDR separately	TRUE
Min. peptide Length	7
Min. score	0
Min. unique peptides	0
Min. razor peptides	1
Min. peptides	1
Use only unmodified peptides and	TRUE
Modifications included in protein quantification	Oxidation (M);Acetyl (N-term);GlyGly (K)
Peptides used for protein quantification	Razor
Discard unmodified counterpart peptides	TRUE
Min. ratio count	2
Lfq min. ratio count	2
Site quantification	Use least modified peptide
Re-quantify	TRUE*
Keep low-scoring versions of identified peptides	No
MS/MS recalibration	FALSE
Match between runs	TRUE*
Time window [min]	2*
Find dependent peptides	FALSE
Labeled amino acid filtering	TRUE
Site tables	GlyGly (K)Sites.txt*;Oxidation (M)Sites.txt
Cut peaks	TRUE
Randomize	FALSE
Special AAs	KR
Include contaminants	TRUE
RT shift	FALSE
Advanced ratios	FALSE
AIF correlation	0.8
First pass AIF correlation	0.8
AIF topx	50
AIF min mass	0
AIF SIL weight	4
AIF ISO weight	2

AIF iterative	FALSE
AIF threshold FDR	0.01

Manual Data Processing of 'Global' Analyses.

Unfiltered datasets resulting from MaxQuant analyses were handled in the form of Excel spreadsheets, and included three SILAC ratios (M/L, H/L and H/M) reflecting the relative abundance of proteins in the three different experimental conditions. As detailed below, data were manually filtered in order to remove contaminants, normalize SILAC ratios, and to define SUMO substrates as well as changes in substrate SUMOylation in response to treatment.

First, all MaxQuant-defined unwanted hits (e.g. 'reverse' (peptide sequences that would match other sequences if reversed), 'contaminants', and 'identified by site' (only identified by modification site)) as well as any remaining suspected internal contaminants (e.g. keratins, immunoglobulins, non-human origin proteins) were removed. All such pre-filtered content was copied into 2 identical tabs and named 'Crude' and 'Purified'. In the 'Crude' list, all hits with 0 unique crude peptides and/or no crude ratios reported were removed. In the 'Purified' list, all hits with 0 unique purified peptides and/or no purified ratios reported were removed. Although MaxQuant uses built-in normalization algorithms to account for variable isotope purity/incorporation or error in lysate mixing, it is only applicable to values distributed in a unimodal Gaussian manner. We therefore applied an alternative method of normalization for our 'purified' data, which due to their highly purified nature and substantial changes in SUMOylation means they are not unimodal. Given that the majority of proteins from our crude samples were unaffected by any

treatment (**Figs. S3D & S3E**), the median of the raw M/L, H/L, and H/M ratios for proteins was calculated and applied to normalize the raw ratios from crude samples as well as from corresponding TAP-purified samples. In order to calculate normalized values, raw ratios were divided by the normalization factors below:

Experiment	Norm. factor M/L	Norm. factor H/L	Norm. factor H/M	Filter cut-off value M/L	Filter cut-off value H/L	Filter cut-off value H/M
IAV SUMO1	1.05	0.95	0.9	1.75	1.58	1.08
IAV SUMO2	0.99	0.92	0.92	1.61	1.54	1.12
HS SUMO2	0.87	0.87	1.0	1.63	1.82	0.82

Next, log2 values of normalized ratios were calculated to facilitate further graphical representation of data in the form of tsMAPs (triple SILAC maps). We assumed that total abundance of the majority of the ~5000 proteins identified in crude lysates does not change significantly with treatment. Thus, the variable distribution of SILAC ratios in the 'crude' samples was deemed representative of the distribution of 'contaminants' in the 'purified' samples. We calculated the total abundance change of the 99% of all proteins closest to zero (log2 values) for all three SILAC ratios in our crude samples (see **Figs. S3D & S3E**), and used the maximum abundance change for these 99% of proteins as

‘cut-off values’ (table above) for filtering the data from the ‘purified’ dataset (see **Figs S3F & S3G**). Further filtering of ‘purified’ ratios was divided into two phases. Firstly, putative SUMO substrates were defined by filtering ratios of TAP-SUMO over TAP and thus applying appropriate cut-off values to M/L and H/L ratios. All ‘hits’ with ratios above certain values (see table above) for either M/L or H/L were called SUMO substrates, and the remainder classified as contaminants. Secondly, changes in substrate SUMO modification by particular treatments were defined by filtering ratios of TAP-SUMO (treatment) over TAP-SUMO (no treatment) and applying appropriate cut-off values to the H/M ratios (table above). All hits with ratios above a certain value (on the positive part of the axis) were defined as substrates with increased SUMOylation in response to treatment, while ‘hits’ below a certain value (on the negative part of the axis) were defined as substrates with decreased SUMOylation (values stated in table above).

A search for ubiquitylation sites (GlyGly) was also included in the processing of the raw mass spectrometry data from purified samples. However, very few (~20) ubiquitylation sites were identified (**Tables S1 & S2**), none of which were subsequently classified as SUMO substrates that change in abundance with IAV infection, and therefore were not followed-up in this study. Notably however, within our A549 SUMO-modified proteome, we identified ubiquitylation sites on ubiquitin itself at lysines 48 and 63. These sites may represent certain topologies of hybrid SUMO-ubiquitin chains (Praefcke et al., 2012).

Manual Data Processing of ‘Slice-by-Slice’ Analyses to Confirm SUMO Modification.

For selected putative SUMO substrates, predicted molecular weight (preMW) was compared to their observed electrophoretic mobility (obsEM) in both ‘crude’ and

‘purified’ datasets. Such a method has been developed previously to confirm ubiquitin conjugation to target proteins (Peng and Cheng, 2005). We assumed that a protein should run according mostly to its mass by SDS-PAGE, and should thus be detected within the gel slice (1-13) covering its approximate MW. For almost all proteins in crude lysates, the preMW was found to correspond to its obsEM when individual protein abundance in each gel slice was analyzed. However, *bona fide* covalent SUMO substrates would have an increased MW, and should therefore be detected in slices corresponding to larger masses. The more highly modified the substrate, the larger the obsEM/preMW ratio should be. This would be most apparent in TAP-SUMO-purified samples given that SUMO is usually only attached to a small proportion of the total population of a protein. Thus, for selected substrates, protein intensity values in individual gel slices from TAP-SUMO-purified material were compared between SILAC conditions. Raw intensity values for M and H data were normalized with the M/L and H/L normalization factors, respectively (table above). All three SILAC ratios were normalized as described for ‘global’ analyses. Among all tested proteins with sufficient data, obsEM/preMW ratios were close to 1 for >95% of crude and contaminant proteins (no SUMO modification), while ratios for putative SUMO substrates were usually infinitely high (no protein observed at its preMW) for >95% of putative SUMO substrates. Protein intensities (abundance) and SILAC ratios within each slice were taken into account when creating graphical representations of these data as shown in **Figs. 4B** and **S5**.

Data Comparisons with Other Studies.

To compare SUMOylation responses between stresses, H/M ratios (i.e. treatment vs non-treatment) for SUMO substrates common to two different datasets were visualized on

tsMAPs with Pearson's coefficient values indicated (**Fig. 5**). All datasets were generated using the same or very similar experimental and data processing methodology (Fritah et al., 2014; Golebiowski et al., 2009; Tatham et al., 2011; Yin et al., 2012). Comparison analyses were made with IPA (Ingenuity Pathway Analysis) software by matching respective gene names and ratios. To assess the reliability of our data, we also compared the substrates identified from the combined SUMO1 and SUMO2 IAV experiments in this study with either the respective crude lysate proteomes (this study) or lists of identified SUMO substrates from other studies (we first generated a database of SUMO substrates defined by publically-available proteomic studies either from our own laboratories or from independent groups (Barysch et al., 2014; Blomster et al., 2009; Fritah et al., 2014; Ganesan et al., 2007; Golebiowski et al., 2009; Hendriks et al., 2014; Matafora et al., 2009; Pungaliya et al., 2007; Rosas-Acosta et al., 2005; Schimmel et al., 2008; Tammsalu et al., 2014; Tatham et al., 2011; Vertegaal et al., 2006)). Comparisons were performed and output collated from IPA software prior to results being depicted as Venn diagrams (**Figure S3H-I**).

PCR-Based Analyses.

RT-qPCR.

Total RNA was isolated using the RNAeasy Plus Kit (Qiagen). RT-qPCRs were performed as a two-step process, and each sample was normalized to an endogenous control (18S rRNA or GAPDH). Complementary DNA (cDNA) was generated by reverse transcription of total RNA using TaqMan Reverse Transcription Reagents (Life Technologies) with an oligo dT primer. Reverse transcription reactions were as follows: (i) primer annealing at 25°C for 10 min, (ii) strand elongation at 37°C for 1h, and (iii)

reverse transcriptase inactivation at 95°C for 5 min. For the second step, sample cDNAs were analyzed in triplicate using the TaqMan Fast Universal PCR Master Mix system (Life Technologies). Each sample was run as a singleplex reaction mixture containing the appropriate TaqMan probe-primer mix: PAF1 (assay ID Hs00219496_m1); C18orf25 (assay ID Hs00973951_g1); AFF4 (assay ID Hs00232683_m1); SUMO1 (assay ID Hs00830844_g1); SUMO2 (assay ID Hs02743873_g1); SUMO3 (assay ID Hs00739248_m1). Probe-primer mixes (FAM/MGB probes) and pre-validated 18S rRNA endogenous control (accession number X03205.1) were purchased from Life Technologies. Real-time reactions were performed in a 7500 Fast Real-Time PCR machine (Life Technologies). Reactions were heated to 95°C for 20s to allow denaturation of the cDNA. PCR was performed as follows: (i) dsDNA strand-separation at 95°C for 3 s; (ii) primer annealing and strand elongation at 60°C for 30s. Steps (i) and (ii) were repeated 40 times. Data were analyzed using 7500 Fast system software (v2.0.5; Life Technologies).

CDC73 and ISG15 mRNA levels (see **Figure 7A**) were determined using the Sybr green RT-qPCR methodology as described previously (Lanz et al., 2015). CDC73-specific primers (5-ACCATTTGCCTTGAACCTTG and 5-GGCCTAAACGTTCACCAAAA) and ISG15-specific primers (5-TGTCGGTGTCAGAGCTGAAG and 5-GCCCTTGTTATTCCTCACCA) were used.

Xbp1 Splicing Assay.

XBP1 mRNA levels were determined using the One-Step AccessQuick RT-PCR system (Promega). Briefly, 1μg of total RNA was reverse-transcribed with AMV Reverse

Transcriptase (AMV RT) and the cDNA amplified with *Tfl* DNA polymerase using XBP1 specific primers (5-AGTGGCCGGGTCTGCTGAGT and 5-GGCTTCCAGCTTGGCTGATGACG). Following separation on a 1% agarose gel, the RT-PCR products corresponding to the unspliced and spliced (26 nucleotide deletion) forms of XBP1 mRNA were visualized by ethidium bromide staining.

RNA Interference.

Cells were transfected in suspension with siRNA duplexes targeting NS1/NEP (AACGGAGGACTTGAATGGAAT), CDC73 (4 individual siRNAs from FlexiTube GeneSolution GS79577, Qiagen), or a control siRNA duplex (Allstars Negative Control siRNA, SI03650318, Qiagen) at a final concentration of 30nM, using Lipofectamine RNAiMAX (Life Technologies). 48h post-transfection, cells were treated as indicated.

Luciferase Reporter Assays.

Transfections were performed in 24- or 96- well plates using Fugene HD (Promega) or Lipofectamine 3000 (Life Technologies). pGL3-Mx1P-FFluc (ISRE-containing promoter), pNFκB-FFLuc (NFκB promoter) and p125-FFLuc (IFNβ promoter) have been described previously (Kochs et al., 2007). pRL-SV40 (Promega) was used as an internal transfection/normalization control. Expression plasmids for GST and PIV5-V have been described previously (Hale et al., 2010; Kochs et al., 2007). FLAG-SENP2 was a gift from Edward Yeh (Addgene plasmid #18047)(Kang et al., 2010). The nucleotide sequences of CDC73 or mCherry were PCR amplified from existing pcDNA3-HRPT2 (gift from Matthew Meyerson (Addgene plasmid #11048)(Rozenblatt-Rosen et al., 2005)) or pmCherry-C1 vectors, respectively, and ligated in-frame into p3xFLAG-CMV-7.1

(Sigma-Aldrich) so as to express with N-terminal FLAG tags. Indicated CDC73 mutants were generated by Quikchange II Site-Directed Mutagenesis (Agilent Technologies) according to the manufacturer's instructions. Newly-generated constructs were authenticated by DNA sequencing.

Detection of Endogenous and Exogenous SUMOylated Proteins.

For detection of SUMOylated overexpressed CDC73, FLAG-tagged CDC73 constructs were co-transfected into 293T cells with pCAGGS expression vectors encoding 6His-tagged SUMO2-GG or 6His-tagged SUMO2-AA. Cells were harvested 36h post-transfection, and 6His-tagged SUMO proteins and conjugates were purified under denaturing conditions essentially as described previously (Tatham et al., 2009). To evaluate the SUMOylation status of endogenous proteins, cells were lysed in buffer containing 50mM Tris-HCl (pH 7.8), 650mM NaCl, 1% NP-40, 2% SDS, 5mM EDTA, and freshly supplemented with 20mM iodoacetamide, 10mM β-mercaptoethanol and cOmplete™, Mini, EDTA-free Protease Inhibitor Cocktail tablets (Roche). SDS levels were subsequently diluted to ~1% and β-mercaptoethanol levels to ~5mM. Lysates were then passed through a 29G needle six times. Following centrifugation at 14,000 rpm for 30 minutes at 10°C, soluble fractions were incubated with end-over-end mixing overnight at 4°C with either anti-SUMO1 (Abcam, ab32058) or anti-SUMO2/3 (Abcam, ab53194) antibodies. Immune complexes were precipitated using Protein-G beads and washed extensively in buffer containing 50mM Tris-HCl (pH 7.8), 650mM NaCl, 1% NP-40, 0.1% SDS, 5mM EDTA, and freshly supplemented with 20mM iodoacetamide and cOmplete™, Mini, EDTA-free Protease Inhibitor Cocktail tablets (Roche). Purified

proteins were eluted from the beads using 2X urea disruption buffer (6M urea, 2M β-mercaptoethanol, 4% SDS), and proteins of interest were detected by western blotting.

Statistical Analyses.

Statistical analyses were performed using an unpaired two-tailed Student's t-test. The *p* values for significance are stated in the figure legends.

Bioinformatic Pathway Analyses.

Bioinformatic pathway analyses were performed using the Enrichr platform (Chen et al., 2013).

SUPPLEMENTAL REFERENCES

Barysch, S.V., Dittner, C., Flotho, A., Becker, J., and Melchior, F. (2014). Identification and analysis of endogenous SUMO1 and SUMO2/3 targets in mammalian cells and tissues using monoclonal antibodies. Nature protocols *9*, 896-909.

Baum, A., Sachidanandam, R., and Garcia-Sastre, A. (2010). Preference of RIG-I for short viral RNA molecules in infected cells revealed by next-generation sequencing. Proceedings of the National Academy of Sciences of the United States of America *107*, 16303-16308.

Blomster, H.A., Hietakangas, V., Wu, J., Kouvonen, P., Hautaniemi, S., and Sistonen, L. (2009). Novel proteomics strategy brings insight into the prevalence of SUMO-2 target sites. Molecular & cellular proteomics : MCP *8*, 1382-1390.

Boutell, C., Cuchet-Lourenco, D., Vanni, E., Orr, A., Glass, M., McFarlane, S., and Everett, R.D. (2011). A viral ubiquitin ligase has substrate preferential SUMO targeted ubiquitin ligase activity that counteracts intrinsic antiviral defence. PLoS pathogens *7*, e1002245.

Chen, E.Y., Tan, C.M., Kou, Y., Duan, Q., Wang, Z., Meirelles, G.V., Clark, N.R., and Ma'ayan, A. (2013). Enrichr: interactive and collaborative HTML5 gene list enrichment analysis tool. BMC bioinformatics *14*, 128.

Chen, S., Short, J.A., Young, D.F., Killip, M.J., Schneider, M., Goodbourn, S., and Randall, R.E. (2010). Heterocellular induction of interferon by negative-sense RNA viruses. Virology *407*, 247-255.

Cox, J., and Mann, M. (2008). MaxQuant enables high peptide identification rates, individualized p.p.b.-range mass accuracies and proteome-wide protein quantification. Nature biotechnology *26*, 1367-1372.

Fritah, S., Lhocine, N., Golebiowski, F., Mounier, J., Andrieux, A., Jouvion, G., Hay, R.T., Sansonetti, P., and Dejean, A. (2014). Sumoylation controls host anti-bacterial response to the gut invasive pathogen Shigella flexneri. EMBO reports *15*, 965-972.

Ganesan, A.K., Kho, Y., Kim, S.C., Chen, Y., Zhao, Y., and White, M.A. (2007). Broad spectrum identification of SUMO substrates in melanoma cells. Proteomics *7*, 2216-2221.

Golebiowski, F., Matic, I., Tatham, M.H., Cole, C., Yin, Y., Nakamura, A., Cox, J., Barton, G.J., Mann, M., and Hay, R.T. (2009). System-wide changes to SUMO modifications in response to heat shock. Science signaling *2*, ra24.

Hale, B.G., Knebel, A., Botting, C.H., Galloway, C.S., Precious, B.L., Jackson, D., Elliott, R.M., and Randall, R.E. (2009). CDK/ERK-mediated phosphorylation of the human influenza A virus NS1 protein at threonine-215. Virology *383*, 6-11.

Hale, B.G., Steel, J., Medina, R.A., Manicassamy, B., Ye, J., Hickman, D., Hai, R., Schmolke, M., Lowen, A.C., Perez, D.R., *et al.* (2010). Inefficient control of host gene expression by the 2009 pandemic H1N1 influenza A virus NS1 protein. Journal of virology *84*, 6909-6922.

Hendriks, I.A., D'Souza, R.C., Yang, B., Verlaan-de Vries, M., Mann, M., and Vertegaal, A.C. (2014). Uncovering global SUMOylation signaling networks in a site-specific manner. Nature structural & molecular biology *21*, 927-936.

Hobbs, S., Jitrapakdee, S., and Wallace, J.C. (1998). Development of a bicistronic vector driven by the human polypeptide chain elongation factor 1alpha promoter for creation of stable mammalian cell lines that express very high levels of recombinant proteins. Biochemical and biophysical research communications *252*, 368-372.

Hoffmann, H.H., Palese, P., and Shaw, M.L. (2008). Modulation of influenza virus replication by alteration of sodium ion transport and protein kinase C activity. Antiviral research *80*, 124-134.

Kang, X., Qi, Y., Zuo, Y., Wang, Q., Zou, Y., Schwartz, R.J., Cheng, J., and Yeh, E.T. (2010). SUMO-specific protease 2 is essential for suppression of polycomb group protein-mediated gene silencing during embryonic development. Molecular cell *38*, 191-201.

Killip, M.J., Young, D.F., Gatherer, D., Ross, C.S., Short, J.A., Davison, A.J., Goodbourn, S., and Randall, R.E. (2013). Deep sequencing analysis of defective genomes of parainfluenza virus 5 and their role in interferon induction. Journal of virology *87*, 4798-4807.

Kochs, G., Garcia-Sastre, A., and Martinez-Sobrido, L. (2007). Multiple anti-interferon actions of the influenza A virus NS1 protein. Journal of virology *81*, 7011-7021.

Landis, H., Simon-Jodicke, A., Kloti, A., Di Paolo, C., Schnorr, J.J., Schneider-Schaulies, S., Hefti, H.P., and Pavlovic, J. (1998). Human MxA protein confers resistance to Semliki Forest virus and inhibits the amplification of a Semliki Forest virus-based replicon in the absence of viral structural proteins. Journal of virology *72*, 1516-1522.

Lanz, C., Yanguez, E., Andenmatten, D., and Stertz, S. (2015). Swine interferon-inducible transmembrane proteins potently inhibit influenza A virus replication. Journal of virology *89*, 863-869.

Manz, B., Brunotte, L., Reuther, P., and Schwemmle, M. (2012). Adaptive mutations in NEP compensate for defective H5N1 RNA replication in cultured human cells. Nature communications *3*, 802.

Matafora, V., D'Amato, A., Mori, S., Blasi, F., and Bachi, A. (2009). Proteomics analysis of nucleolar SUMO-1 target proteins upon proteasome inhibition. Molecular & cellular proteomics : MCP *8*, 2243-2255.

O'Neill, R.E., Talon, J., and Palese, P. (1998). The influenza virus NEP (NS2 protein) mediates the nuclear export of viral ribonucleoproteins. The EMBO journal *17*, 288-296.

Peng, J., and Cheng, D. (2005). Proteomic analysis of ubiquitin conjugates in yeast. Methods in enzymology *399*, 367-381.

Praefcke, G.J., Hofmann, K., and Dohmen, R.J. (2012). SUMO playing tag with ubiquitin. Trends in biochemical sciences *37*, 23-31.

Pungaliya, P., Kulkarni, D., Park, H.J., Marshall, H., Zheng, H., Lackland, H., Saleem, A., and Rubin, E.H. (2007). TOPORS functions as a SUMO-1 E3 ligase for chromatin-modifying proteins. Journal of proteome research *6*, 3918-3923.

Quinlivan, M., Zamarin, D., Garcia-Sastre, A., Cullinane, A., Chambers, T., and Palese, P. (2005). Attenuation of equine influenza viruses through truncations of the NS1 protein. Journal of virology *79*, 8431-8439.

Reichelt, M., Stertz, S., Krijnse-Locker, J., Haller, O., and Kochs, G. (2004). Missorting of LaCrosse virus nucleocapsid protein by the interferon-induced MxA GTPase involves smooth ER membranes. Traffic *5*, 772-784.

Rosas-Acosta, G., Russell, W.K., Deyrieux, A., Russell, D.H., and Wilson, V.G. (2005). A universal strategy for proteomic studies of SUMO and other ubiquitin-like modifiers. Molecular & cellular proteomics : MCP *4*, 56-72.

Rozenblatt-Rosen, O., Hughes, C.M., Nannepaga, S.J., Shanmugam, K.S., Copeland, T.D., Guszczynski, T., Resau, J.H., and Meyerson, M. (2005). The parafibromin tumor suppressor protein is part of a human Paf1 complex. Molecular and cellular biology *25*, 612-620.

Schimmel, J., Larsen, K.M., Matic, I., van Hagen, M., Cox, J., Mann, M., Andersen, J.S., and Vertegaal, A.C. (2008). The ubiquitin-proteasome system is a key component of the SUMO-2/3 cycle. Molecular & cellular proteomics : MCP *7*, 2107-2122.

Shevchenko, A., Tomas, H., Havlis, J., Olsen, J.V., and Mann, M. (2006). In-gel digestion for mass spectrometric characterization of proteins and proteomes. Nature protocols *1*, 2856-2860.

Solorzano, A., Webby, R.J., Lager, K.M., Janke, B.H., Garcia-Sastre, A., and Richt, J.A. (2005). Mutations in the NS1 protein of swine influenza virus impair anti-interferon activity and confer attenuation in pigs. Journal of virology *79*, 7535-7543.

Sunters, A., Armstrong, V.J., Zaman, G., Kypta, R.M., Kawano, Y., Lanyon, L.E., and Price, J.S. (2010). Mechano-transduction in osteoblastic cells involves strain-regulated estrogen receptor alpha-mediated control of insulin-like growth factor (IGF) I receptor sensitivity to Ambient IGF, leading to phosphatidylinositol 3-kinase/AKT-dependent Wnt/LRP5 receptor-independent activation of beta-catenin signaling. The Journal of biological chemistry *285*, 8743-8758.

Tammsalu, T., Matic, I., Jaffray, E.G., Ibrahim, A.F., Tatham, M.H., and Hay, R.T. (2014). Proteome-wide identification of SUMO2 modification sites. Science signaling *7*, rs2.

Tatham, M.H., Matic, I., Mann, M., and Hay, R.T. (2011). Comparative proteomic analysis identifies a role for SUMO in protein quality control. Science signaling *4*, rs4.

Tatham, M.H., Rodriguez, M.S., Xirodimas, D.P., and Hay, R.T. (2009). Detection of protein SUMOylation in vivo. Nature protocols *4*, 1363-1371.

Trilling, M., Le, V.T., Rashidi-Alavijeh, J., Katschinski, B., Scheller, J., Rose-John, S., Androsiac, G.E., Jonjic, S., Poli, V., Pfeffer, K.*, et al.* (2014). "Activated" STAT

proteins: a paradoxical consequence of inhibited JAK-STAT signaling in cytomegalovirus-infected cells. Journal of immunology *192*, 447-458.

Vertegaal, A.C., Andersen, J.S., Ogg, S.C., Hay, R.T., Mann, M., and Lamond, A.I. (2006). Distinct and overlapping sets of SUMO-1 and SUMO-2 target proteins revealed by quantitative proteomics. Molecular & cellular proteomics : MCP *5*, 2298-2310.

Yin, Y., Seifert, A., Chua, J.S., Maure, J.F., Golebiowski, F., and Hay, R.T. (2012). SUMO-targeted ubiquitin E3 ligase RNF4 is required for the response of human cells to DNA damage. Genes & development *26*, 1196-1208.

Zhang, L., Das, P., Schmolke, M., Manicassamy, B., Wang, Y., Deng, X., Cai, L., Tu, B.P., Forst, C.V., Roth, M.G.*, et al.* (2012). Inhibition of pyrimidine synthesis reverses viral virulence factor-mediated block of mRNA nuclear export. The Journal of cell biology *196*, 315-326.

Zhang, Y., Kien, F., Ma, H.L., Tse, J., Poon, L.L.M., and Nal, B. (2011). Identification of a novel interaction between the M2 proton channel of influenza A virus and cyclin D3: consequences for cell cycle progression. BMC Proc *5*, 70.
